# EOMES interacts with RUNX3 and BRG1 to promote innate memory cell formation through epigenetic reprogramming

**DOI:** 10.1038/s41467-019-11233-6

**Published:** 2019-07-24

**Authors:** Nicolas Istaces, Marion Splittgerber, Viviana Lima Silva, Muriel Nguyen, Séverine Thomas, Aurore Le, Younes Achouri, Emilie Calonne, Matthieu Defrance, François Fuks, Stanislas Goriely, Abdulkader Azouz

**Affiliations:** 1 Université Libre de Bruxelles, Institute for Medical Immunology (IMI), Gosselies, 6041 Belgium; 2 Université Catholique de Louvain, Institut de Duve, Brussels, 1200 Belgium; 3 Université Libre de Bruxelles, Laboratory of Cancer Epigenetics, Brussels, 1070 Belgium; 4 Université Libre de Bruxelles, Interuniversity Institute of Bioinformatics in Brussels (IB2), Brussels, 1050 Belgium

**Keywords:** Immunological memory, Epigenetics in immune cells, CD8-positive T cells, Chromatin remodelling

## Abstract

Memory CD8^+^ T cells have the ability to provide lifelong immunity against pathogens. Although memory features generally arise after challenge with a foreign antigen, naïve CD8 single positive (SP) thymocytes may acquire phenotypic and functional characteristics of memory cells in response to cytokines such as interleukin-4. This process is associated with the induction of the T-box transcription factor Eomesodermin (EOMES). However, the underlying molecular mechanisms remain ill-defined. Using epigenomic profiling, we show that these innate memory CD8SP cells acquire only a portion of the active enhancer repertoire of conventional memory cells. This reprograming is secondary to EOMES recruitment, mostly to RUNX3-bound enhancers. Furthermore, EOMES is found within chromatin-associated complexes containing BRG1 and promotes the recruitment of this chromatin remodelling factor. Also, the in vivo acquisition of EOMES-dependent program is BRG1-dependent. In conclusion, our results support a strong epigenetic basis for the EOMES-driven establishment of CD8^+^ T cell innate memory program.

## Abstract (web summary)

T box transcription factor Eomesodermin (EOMES) is induced when naïve T cells are converted to innate memory T cells in response to cytokines. Here the authors show that EOMES is recruited to RUNX3-bound enhancers and interacts with BRG1 during innate memory T cell formation.

## Introduction

CD8^+^ T lymphocytes are critical for protective immunity against intracellular pathogens and tumor cells. When naïve CD8^+^ T cells encounter their cognate antigen (Ag) in secondary lymphoid organs and expand, most daughter cells undergo terminal differentiation into effector cells while a small fraction will form long-lived memory and persist after pathogen clearance^1^. Such memory cells are epigenetically programmed through highly dynamic changes in enhancer repertoires and regulatory circuits for more rapid and effective responses upon re-stimulation with their Ag or inflammatory cytokines^2,3^. New epigenomic and bioinformatics tools have been successfully used to identify potential transcription factors regulating this process^3–5^. Furthermore, specific epigenetic mechanisms, such as H3K27me3 deposition at pro-memory genes in effector cells were shown to dictate memory cell potential^6^.

In the periphery, memory CD8^+^ T cell subpopulations can be identified and classified by the expression of surface markers, such as CD44, CD62L, Ly6C, CD103, CD122 (IL-2Rβ chain), or CXCR3. These adhesion molecules, cytokine or chemokine receptors define their functional properties and trafficking. Importantly, acquisition of these phenotypic markers can also occur in Ag-inexperienced cells^7–9^. It has long been known that naïve CD8^+^ T cells in a lymphopenic environment undergo conversion to memory-like phenotype CD8^+^ T cells independently of foreign Ag exposure and in response to homeostatic cytokines. Similar processes also occur under physiological conditions in immunocompetent hosts. In particular, naïve CD8SP αβ T cells that have undergone normal thymic selection can acquire a memory phenotype before leaving the thymus. This unique population was initially discovered in ITK-deficient mice^10,11^. Expansion of CD44^hi^CD122^hi^ CD8SP thymocytes was also observed in other strains, such as KLF2-deficient, CBP-deficient, or ID3-deficient mice^8^. Since they share functional and phenotypic features with innate T cells, such as invariant NKT or γδ T cells, they were referred to as innate memory CD8^+^ T cells (T_IM_ cells)^8^. It has been demonstrated that the expansion of T_IM_ cells is the result of IL-4 overproduction by PLZF^+^ NKT or γδ T cells^12^. Of note, T_IM_ cells represent a significant proportion of CD8SP thymocytes in wild-type (WT) Balb/c mice as thymic PLZF^+^ NKT cells producing IL-4 are physiologically abundant in this strain^13^.

When compared to naïve cells, T_IM_ cells display an increased capacity to control chronic LCMV infection through the rapid production of cytokines^14^. Similar conclusions were reached for virtual memory (T_VM_) CD8^+^ T cells that physiologically arise in the periphery under the influence of homeostatic signals^9^. Furthermore, the IL-4-driven expansion of T_VM_ cells upon injection of the Schistosoma egg antigen provides a significant protection against acute MuHV4 infection^15^. T_VM_ cells were also shown to surpass naïve T cells in their capacity to produce cytokines and protect against *L. monocytogenes* both in Ag-specific and bystander fashions^16,17^. However, when compared to true conventional memory (T_M_) cells, both T_IM_ and T_VM_ cells display reduced functional features^14,16,18^.

Conversion of naïve CD8SP thymocytes into T_IM_ cells indicates that acquisition of memory traits and T-cell receptor (TCR) triggering can be uncoupled. T_IM_ cells express high levels of the T-box transcription factor Eomesodermin (EOMES) and its loss impedes their development^19,20^. However, little is known about its specific role. Herein, we explore the molecular processes that accompany unconventional memory formation. Epigenomic profiling of naïve and T_IM_ CD8SP thymocytes reveals global modifications of the enhancer landscape that only partially recapitulate what happens in T_M_ cells. We provide evidence that EOMES contributes to this epigenetic programming, in part through the recruitment of the SWI/SNF machinery.

## Results

### Transcriptional features of T_IM_ cells

**Fig. 1:**
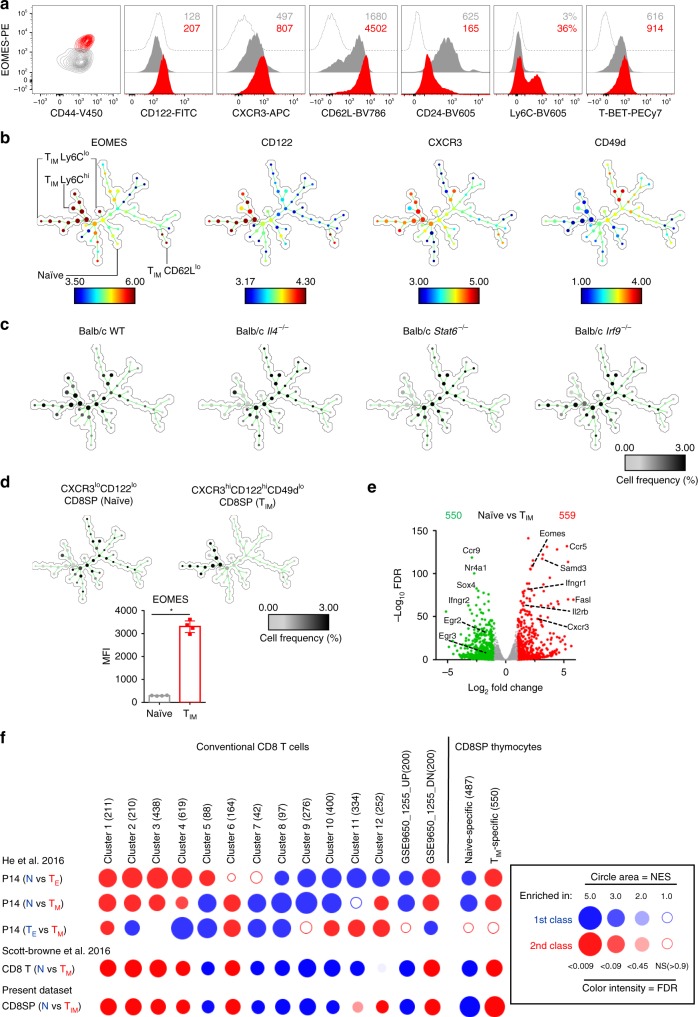
T_IM_ cells display classical features of conventional memory cells. **a** Flow cytometry of CD3^+^CD8SP thymocytes from wild-type (WT) Balb/c mice. Histograms represent the expression of the indicated protein in EOMES^hi^ (red) and EOMES^lo^ (gray) cells or the fluorescence minus one (FMO) controls (empty). Median fluorescence intensity (MFI) or the proportion of positive cells are displayed. **b** SPADE of flow cytometry data gated on CD3^+^CD8SP thymocytes from WT Balb/c mice. Circles represent cell nodes, colors indicate expression levels for the indicated marker and size is related to the number of cells within a node. Annotations indicate the identified innate memory (T_IM_) subsets and the bulk of naïve cells. Trees displaying expression levels of other markers are shown in Supplementary Fig. 1. **c** SPADE trees showing cell frequency of CD3^+^CD8SP thymocytes from the indicated strain (**b**, **c**, 4 WT Balb/c mice were used for tree construction. In **c**, one representative mouse out of four is shown for each group). **d** SPADE trees showing cell frequency in each node for naïve and T_IM_ cells sorted from WT Balb/c mice (gating, Supplementary Fig. 2). EOMES expression (horizontal bars indicate median ± interquartile range) in sorted naïve and T_IM_ cells are shown. Each point represents an individual sample. Statistics were calculated using Mann–Whitney test. *P < 0.05. **e** Volcano plot of RNA-seq data from naïve versus T_IM_ cells shows the adjusted *P*-value (–log_10_) versus fold change (log_2_) (up in T_IM_, red; up in naïve, green). The numbers of differentially expressed genes are indicated. RNA-seq was performed in triplicates (each sample was generated from a pool of at least seven mice). **f** BubbleGUM gene set enrichment analysis (GSEA) map of datasets from naïve (N), conventional effector (T_E_), and memory (T_M_) cells or from naïve and T_IM_ CD8SP thymocytes. Gene sets (with the indicated number of genes) were established by analysis of their expression patterns between N, T_E_, and T_M_, from available gene sets or from naïve and T_IM_-specific genes as defined in **e**. The panel summarizes the normalized enrichment score (NES) and false discovery rate (FDR) parameters

T_IM_ cells in ITK-deficient or KLF2-deficient mice were initially defined as CD44^+^CD122^+^EOMES^hi^ CD8SP cells^10–12^. In order to further define the phenotypic status of T_IM_ cells in WT Balb/c mice, we first looked at the expression of cell markers in EOMES^lo^ or EOMES^hi^ CD3^+^CD8SP thymocytes (Fig. 1a). Besides higher CD122 levels, EOMES^hi^ CD3^+^CD8SP thymocytes also expressed higher levels of CXCR3 and central memory cell markers (CD62L, Ly6C). T-BET expression was also slightly increased. In contrast, they expressed reduced levels of CD24, a feature of more mature CD8SP cells. Spanning-tree progression analyses of density-normalized events (SPADE)^21^ centered on CD3^+^CD8SP thymocytes revealed cell clusters sharing similar phenotypes (Fig. 1b, Supplementary Fig. 1). T_IM_ cells were distributed among subsets mainly defined by CD103, Ly6C, and CD62L expression. Cell heterogeneity within EOMES^lo^ cells showed more complex bimodal expression patterns: subsets were mainly defined by CD62L, CD49d, and CD103 expression. Several clusters (EOMES^int^CD24^int^ cells) were identified as cells that are likely to be in the active process of transitioning from EOMES^lo^ to T_IM_ cells. In order to identify the dependency of these cell subsets on IL-4/STAT6 and Type I IFNs/ISGF3 pathways shown to be required for their development^22^, we compared the cell frequencies of these cell subsets between WT, *Stat6*-deficient, *Il4*-deficient, and *Irf9*-deficient Balb/c mice (Fig. 1c). Loss of *Stat6* or *Il4* expression both resulted in the complete absence of T_IM_ cells, while *Irf9*-deficient Balb/c mice retained the presence of Ly6C^lo^ T_IM_ cells. Together, these results suggest that EOMES^hi^ T_IM_ cells arise from EOMES^lo^CD3^+^CD8SP thymocytes under the influence of IL-4 and type I IFNs in a stepwise manner, acquiring during this process a pattern of memory cell markers reminiscent of central memory cells.

In order to gain further insight into the biology of these cells and the role of EOMES in their developmental program, we sorted CD44^hi^CD122^hi^CXCR3^hi^CD49d^−^ (STAT6-dependent EOMES^hi^ T_IM_) and CD44^lo^CD122^−^CXCR3^−^ (naïve) CD8SP thymocytes (Fig. 1d, Supplementary Fig. 2). We performed global transcriptional profiling and identified statistically downregulated or upregulated genes between these two populations (Fig. 1e, Supplementary Data 1). Consistent with their innate memory phenotype, we identified cytokine/chemokine receptors, chemokines or effector molecules among genes that were upregulated in T_IM_ cells. Conversely, expression of *Ccr9, Ccr4, Ifngr2*, and *Fas* was downregulated in T_IM_ cells. In addition to *Eomes*, other genes encoding transcription factors including *Stat4, Tbx21, Mef2a*, and *Pou6f1*, were upregulated in T_IM_ cells. Differentiation into T_IM_ cells was also accompanied by a decreased expression of genes encoding *Sox4, Ikzf2, Egr2, Egr3, Id2, Nr4a1, or Bcl6*. These results suggest that an important transcriptional reprogramming accompanies the acquisition of a memory phenotype by CD8SP thymocytes. To define the relationship between T_IM_ cells, conventional effector (T_E_), and central memory (T_CM_) CD8^+^ T cells, we performed gene set enrichment analysis (GSEA) of 12 gene clusters that were established based on their expression patterns between naïve (N), T_E_ (KLRG1^hi^IL7R^lo^, day 8), and T_CM_ (CD62L^+^, >day 60) P14 transgenic T cells in the context of lymphocytic choriomeningitis virus (LCMV) infection (Fig. 1f)^3^. As additional comparators, we used gene sets from another source (naïve vs. memory [T_M_] CD8^+^ T cells^23^). We first tested these clusters in an independent dataset from LCMV-infected mice^4^. These clusters were enriched in naïve vs. endogenous gp33-specific T_M_ cells with a pattern very similar to P14 T_CM_ cells. We observed that these clusters of genes also behaved in a similar fashion in terms of hierarchy when our datasets from naïve and T_IM_ CD8SP cells were compared using the same approach, i.e. T_CM_ signature genes were strongly enriched in T_IM_ cells irrespective of their behavior during the effector phase. All genes in clusters 5 and 7–10 were downregulated in T_CM_ or T_M_ cells compared to naïve CD8^+^ T cells. These expression clusters were also significantly enriched in naïve CD8SP cells when compared to T_IM_ cells. Taken together, these data indicate that there is a strong degree of convergence at the transcriptomic level during conventional and unconventional memory formation. They suggest that part of the underlying molecular processes could be shared.

### The epigenetic landscape of T_IM_ cells

**Fig. 2:**
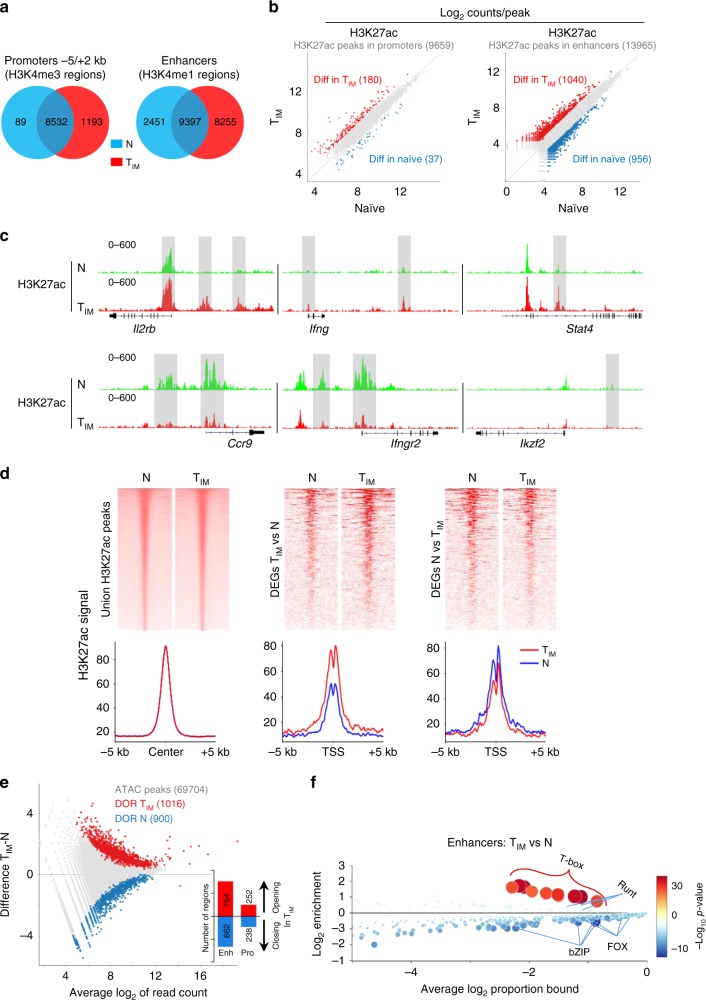
Epigenetic landscape of T_IM_ cells. **a** Venn diagram illustrating the intersection of promoter (left) and enhancer (right) regions in naïve (N) and innate memory (T_IM_ CD8SP cells). **b** Scatter plots presenting counts of reads per H3K27ac peaks at promoters (left) and enhancers (right) with the indicated number of regions in naïve and T_IM_ samples. Differentially active regions in T_IM_ or naïve cells are shown in red and blue, respectively. **c** Representative tracks of differentially active H3K27ac peaks on IGV genome browser (highlighted in grey). **d** H3K27ac read densities on the union of all peaks (left) or centered on the TSS (±5 kb) of differentially expressed genes (DEG) in T_IM_ (middle) and naïve cells (right). Each heatmap is accompanied by a plot showing the normalized cumulative coverage around the centre of the regions. **e** MA plot of mean log_2_ ATAC-seq peak atlas showing the differentially open regions (DOR) of T_IM_ (red) and naïve cells (blue) with the indicated number of regions. Histograms indicate the distribution and numbers of DOR at promoters and enhancers. **f** CiiiDER analysis of putative transcription factors motifs in enhancer-bound DOR. Transcription factors are coloured according to the *P*-value of their gene coverage and whether they are over- (red) or under- (blue) represented in T_IM_ cells. The size of each point is also proportional to log_10_ *P*-value. ChIP-seq was performed on three independent IPs (*n* = 5 mice per sample). ATAC-seq was performed on 1–3 independent samples (*n* = 2 mice per sample) from each group

Next, we mapped genome-wide promoters and enhancers using H3K4me3 and H3K4me1 profiles in naïve CD8SP and T_IM_ cells. Among the 9814 active promoter elements, 1193 were found to be specific to T_IM_ cells. Conversely, very few (89) were found to be specific to naïve CD8SP thymocytes (Fig. 2a). A high proportion of enhancers (8255/20103) was found to be specific to T_IM_ cells, suggesting that an important epigenetic reprogramming occurs in these cells (Fig. 2a). We then assessed H3K27ac levels in these regions as a surrogate for their activity^24^. We called 9659 and 13965 individual regions in promoter and enhancer regions, respectively, and quantified their read intensity. Using this approach, we identified 37 and 180 differentially active regions within promoters of naïve or T_IM_ cells, respectively (Fig. 2b, Supplementary Data 2). Globally, H3K27ac levels in promoters of T_IM_ signature genes, such as *Il2rb*, *Ifng*, or *Eomes* were found to be strongly increased in T_IM_ cells (Fig. 2c, d, Supplementary Fig. 3). Conversely, H3K27ac deposition in promoters of downregulated (naïve signature) genes, such as *Ccr9* or *Ifngr2* tended to decrease in T_IM_ cells (Fig. 2c, d). Nevertheless, the most important modifications that occur during the shift between naïve and T_IM_ cells were observed in enhancer regions. Indeed, we identified 956 and 1040 differentially active regions within enhancers of naïve or T_IM_ cells, respectively (Fig. 2b, Supplementary Data 2). In parallel, we assessed chromatin accessibility by performing Assay of Transposase-Accessible Chromatin with high throughput sequencing (ATAC-seq). We confirmed that major changes occur in enhancer regions, where we identified 1426 Differentially Open Regions (DOR) in T_IM_ cells, compared to 490 DOR around promoters (Fig. 2e, Supplementary Data 3). We combined H3K27ac data with ATAC-seq profiles to restrict the analysis of transcription factors’ binding motifs to accessible sites within these enhancers (Fig. 2f). We observed a strong and significant enrichment for T-box motifs, consistent with the potential role of EOMES in driving T_IM_ cell development.

**Fig. 3:**
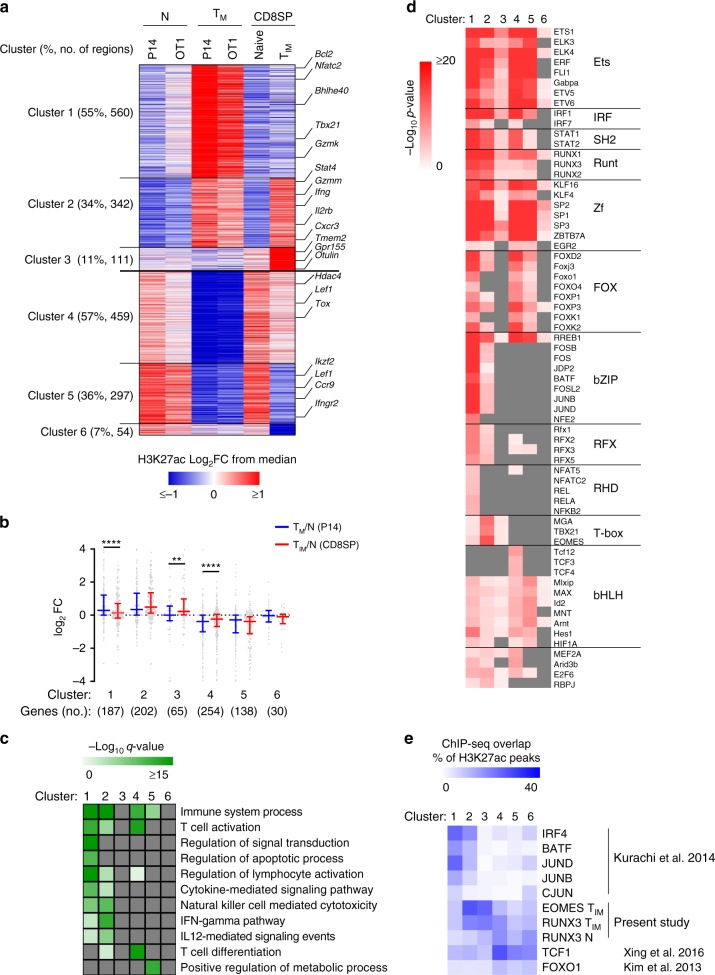
Active enhancers shared by T_IM_ and T_M_ cells are enriched with T-box motifs. **a** Clustering of H3K27ac peaks within enhancers based on their activity in conventional memory cells (naïve/T_CM_ P14 cells-LCMV infection and naïve/T_M_ OT1 cells-Listeria-OVA infection) and in innate memory CD8SP thymocytes (naïve/T_IM_). Enhancers in clusters 1–3 and 4–6 are more or less active in memory cells, respectively. Their relative frequency and the number of regions are shown in the left margin. Selected genes associated with each cluster are displayed in the right margin. Values are represented as Log_2_ fold-change obtained from median of each single region. **b** Relative expressions of cluster-associated genes between conventional memory (T_M_/N) and innate memory CD8SP thymocytes (T_IM_/N). Horizontal lines indicate median ± interquartile range. **c** Selected Gene Ontology pathways enriched in genes associated with clusters 1, 2, 4, and 5 shown in **a** using GREAT with default parameters and presented as −log_10_ of binomial FDR *q*-value. **d** Motif enrichment analysis of differentially active enhancers clusters shown in **a** at the centre of overlapping ATAC-seq peaks using AME and presented as −log_10_ of *P-*values. Transcription factors families are shown in the right margin. **e** Percentage of regions within each enhancer cluster that overlap with ChIP-seq peaks for the indicated transcription factor. ChIP-seq was performed on three independent immunoprecipitations (*n* = 5 mice per sample). Source data are provided as a Source Data file. Statistics for **b** were calculated using Wilcoxon matched-pairs signed rank test. **P < 0.01, ****P < 0.0001. Source Data file

Our transcriptomic data indicated that T_IM_ cells share common features with T_M_ cells. In order to determine whether this is also the case at the epigenetic level, we analyzed H3K27ac ChIP-seq data from naïve/T_CM_ P14 cells (LCMV model) and naïve/T_M_ OT1 cells (Listeria-OVA model)^3,5^. For both models, we performed differential analysis for H3K27Ac peaks that were located in enhancer regions to define consensus sets of enhancers that are more (1105 regions) or less (1241 regions) active in T_M_ cells compared to their naïve counterparts. We then combined these regions with the sets of enhancers that we previously identified in naïve and T_IM_ cells (total of 1996 regions) and looked for common or divergent behaviors in T_M_ vs. T_IM_ cells (Fig. 3a, Supplementary Data 4). For regions that are more active in either T_M_ or T_IM_ cells compared to all their naïve counterparts, 55% were only active in T_M_ cells (cluster 1), 34% were common to T_M_ and T_IM_ (cluster 2), and only 11% were specific to T_IM_ cells (cluster 3). We observed similar trends for regions that were less active in memory subsets: 57% were less active in T_M_ cells only (cluster 4), 36% were common to T_M_ and T_IM_ (cluster 5), and 7% were specific to T_IM_ cells (cluster 6). These results suggest that a major part of the epigenetic reprogramming observed in T_IM_ cells is also encountered in T_M_ cells (i.e. 75% of enhancer regions that are more active and 84% of enhancer regions that are less active in T_IM_ compared to naïve CD8SP thymocytes follow the same differential pattern between T_M_ and their naïve counterparts). However, a large proportion of the events that take place in T_M_ cells (clusters 1 and 4) does not occur during T_IM_ formation. We evaluated the expression of genes associated with these six clusters of enhancers in T_M_ and T_IM_ cells. As shown in Fig. 3b, genes associated with clusters 1–3 and 4–6 were globally upregulated and downregulated in memory cells as compared to their naïve counterparts. Importantly, genes associated with T_M_-specific cluster 1 were significantly more upregulated in T_M_ than in T_IM_ cells. The reverse was true for T_IM_-specific cluster 3, indicating that differences in enhancer landscapes between T_M_ and T_IM_ cells influence the transcriptional activities of associated genes. A gene-ontology analysis^25^ showed a specific enrichment for enhancers of genes encoding components of signal transduction and apoptosis pathways in cluster 1, and of IFNγ, IL-12 and cytotoxic pathways in cluster 2 (Fig. 3c). We also identified different pathways associated with enhancers in clusters 4 and 5, such as T cell differentiation and metabolic processes, respectively. This analysis did not reveal biologically relevant pathways for the limited numbers of genes associated with T_IM_-specific clusters 3 and 6. These observations suggest important functional differences between the enhancers that are specific to T_M_ cells and those that are shared with T_IM_ cells.

To narrow our search for binding motifs, we focused our analysis on the centre of ATAC peaks located in these sets of enhancer regions (Fig. 3d). Runx motifs were found preferentially in enhancers that are more active in memory subsets. We observed striking differences between clusters 1 and 2: T_M_-specific enhancers (cluster 1) were strongly enriched with motifs for bZIP-, Xbox, and RHD-containing proteins while cluster 2 (common to T_M_ and T_IM_) contained T-box motifs. The smaller set of T_IM_-specific enhancers (cluster 3) also harboured T-box motifs. A substantial fraction of ChIP-seq peaks identified for IRF4, BATF, JUNB, JUND, and CJUN in effector CD8^+^ T cells^26^ overlapped cluster 1 regions (Fig. 3d). This is consistent with the fact that activation of BATF/IRF4, AP-1, NF-AT, or NF-κB complexes occurs upon antigenic stimulation. In contrast, EOMES and RUNX3 peaks in T_IM_ cells (see next section) overlapped to a greater extent with clusters 2 and 3 regions (Fig. 3e). There were also some notable differences in the enrichment for Fox and bHLH motifs in clusters 4 and 5. This is correlated with a more important overlap of TCF1 and FOXO1 peaks with cluster 4 than with cluster 5 regions. Globally, these analyses highlight distinct epigenetic programs during conventional and unconventional memory CD8^+^ T cell formation. They reveal that although T_IM_ cells acquire classical features of memory cells at the phenotypic and transcriptomic levels, they are only partially programmed toward memory fate at the epigenetic level.

### EOMES is recruited to RUNX3-bound regions in T_IM_ cells

**Fig. 4:**
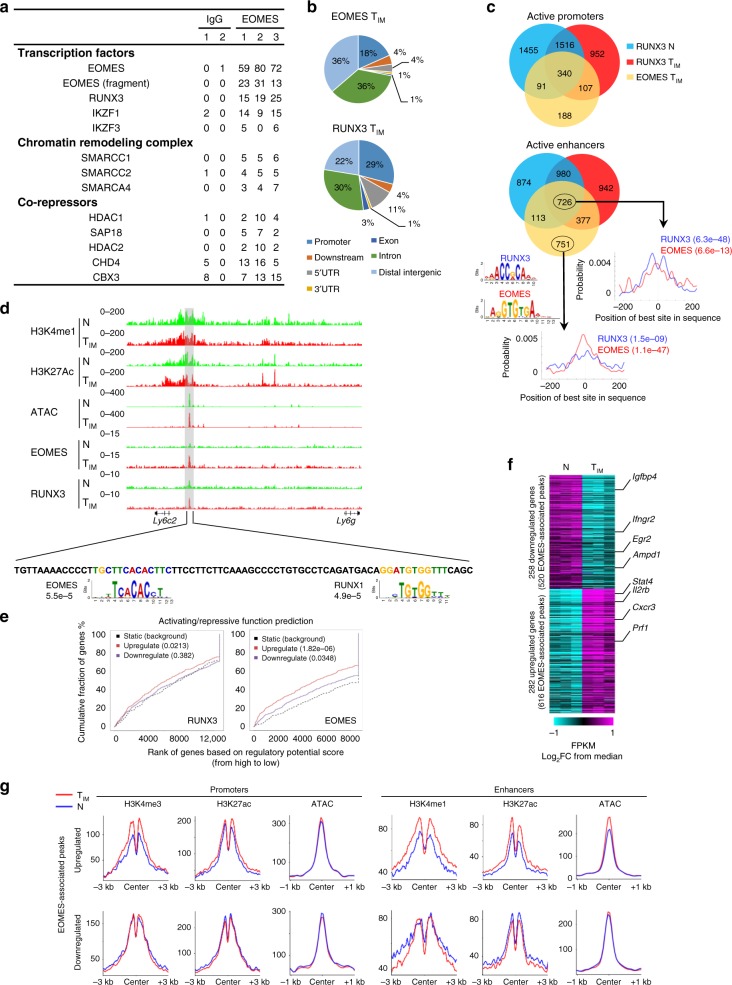
EOMES and RUNX3 interact within chromatin-associated complexes. **a** Selected EOMES-interacting proteins identified by RIME in activated CD8^+^ T cells. Total spectral counts for each replicate (anti-EOMES or control IgG) are shown. **b** Genomic distribution of EOMES (4306) and RUNX3 (6741)-binding sites in innate memory (T_IM_) cells. **c** Venn diagram illustrating the intersection between EOMES (in T_IM_) and RUNX3 (in naïve [N] and T_IM_ cells) peaks at active promoters and enhancers. Density plots centered on common EOMES/RUNX3 or EOMES-specific peaks (±250 bp) represent the distribution of the best predicted sites of the JASPAR RUNX3 (MA0684.1) and EOMES (MA0800.1) motifs. **d** Representative EOMES, RUNX3, H3K4me1, H3K27Ac ChIP-seq, and ATAC-seq tracks at the *Ly6c2* locus showing the co-localisation of EOMES and RUNX3 highlighted in grey (top). Sequence of the highlighted region and the location of EOMES and RUNX motifs with their position *P*-value are indicated (down). **e** Cumulative distribution plot generated by BETA algorithm showing the predicted activating/repressive functions of RUNX3 and EOMES with the indicated *P*-values determined by the Kolmogorov–Smirnov test. **f** Heatmap showing expression of genes from **e** that are predicted to be targets of EOMES. Selected genes are shown in the right margin. **g** Normalized coverage plot of histones modifications (H3K4me1, H3K4me3, and H3K27ac) and chromatin accessibility (ATAC-seq) centered on EOMES-binding sites at promoters and enhancers annotated to predicted genes from **e**. ChIP-seq was performed on three independent IPs (*n* = 7 mice per sample)

Our results strongly suggest that EOMES contributes to epigenetic reprogramming in T_IM_ cells, and that similar processes occur during conventional memory formation. In order to understand the underlying mechanisms, we first explored the interactome of EOMES. For this purpose, we performed RIME (Rapid Immunoprecipitation mass spectrometry of endogenous protein) experiments in activated primary CD8^+^ T cells. We identified several potentially relevant partners of EOMES (Fig. 4a). These include transcription factors (RUNX3, IKZF1, IKZF3), chromatin remodeling complexes (members of the SWI/SNF machinery) and co-repressors.

As Runt motifs were enriched in enhancer regions that were more active in T_IM_ cells (Fig. 2f), we focused on the potential interaction between EOMES and RUNX3. For this purpose, we performed ChIP-seq experiments against EOMES and RUNX3 in naïve and T_IM_ CD8SP cells to determine their genomic targets. A few EOMES peaks (265) were identified in naïve CD8SP thymocytes. In contrast, we detected 4306 regions bound by EOMES in T_IM_ cells. For RUNX3, we identified 6345 and 6741 peaks in naïve and T_IM_ cells, respectively. Although RUNX3 was expressed at comparable levels in naïve and T_IM_ CD8SP thymocytes, we also observed an important proportion of cell stage-specific genomic targets, 2329 and 1894 in naïve and T_IM_ cells, respectively. The proportion of RUNX3 peaks that overlap with promoters or 5′UTR (40%) was more important than for EOMES (22%). The majority of EOMES targets in T_IM_ cells were found in distal intergenic regions and introns (Fig. 4b). Indeed, we identified 726 and 1967 EOMES peaks within active promoter and enhancer regions, respectively. Up to 65% of these EOMES peaks overlapped with RUNX3 peaks obtained from naïve and/or T_IM_ cells (Fig. 4c). Within regions that were common for EOMES and RUNX3, binding sites for these two transcription factors were enriched near the centre of the region and identified in close proximity, but did not generally overlap, as shown for an enhancer region in the vicinity of the *Ly6c2* gene (Fig. 4d). Importantly, for almost 43% of EOMES-bound regions, we identified RUNX3 peaks in naïve CD8SP thymocytes, supporting the notion that EOMES is preferentially recruited to regions that were associated beforehand with RUNX3. Along with our proteomic data, these results indicate that EOMES and RUNX3 closely interact within chromatin-associated complexes.

We infered genes that are directly activated or repressed by EOMES^27^ (Fig. 4e). In comparison to downregulated targets, genes that were upregulated in T_IM_ cells had a more significant enrichment for EOMES-binding sites. Of note, we also observed a significant regulatory potential for RUNX3 on T_IM_-specific genes. Using this approach, we identified 540 genes that were potentially directly modulated by EOMES (Fig. 4f). Among the upregulated genes, we found known EOMES targets such as *Il2rb* or *Prf1*^28,29^. EOMES-binding sites were located both in promoter (299 sites) and enhancer (677 sites) regions. In enhancers and to a lesser extent in promoter regions of upregulated genes, we observed an increase in histone marks and ATAC signals around EOMES-binding sites in T_IM_ cells compared to their naïve counterparts (Fig. 4g). In contrast, in regulatory regions associated with downregulated EOMES target genes, the same parameters were not modulated except for a minor decrease in H3K27ac levels around EOMES peaks in enhancer regions. These data indicate that binding of EOMES in T_IM_ cells is associated with a local chromatin remodeling of regulatory elements that supports transcriptional activity.

### EOMES overexpression partially recapitulates the T_IM_ program

**Fig. 5:**
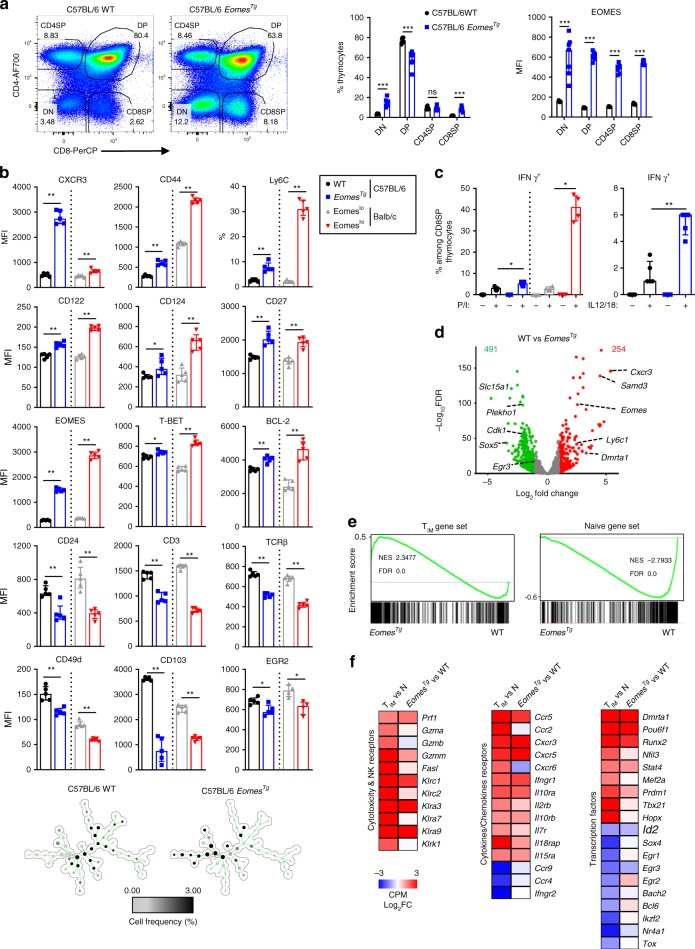
EOMES expression is sufficient to drive T_IM_ transcriptional program. **a** Representative flow cytometry plots of total thymocytes from WT and *Eomes*^*Tg*^ mice showing DN, DP, CD4SP, and CD8SP proportions. Histograms indicate the frequency of each population and their EOMES expression (MFI). **b** Expression of the indicated markers in WT and *Eomes*^*Tg*^ CD3^+^CD8SP thymocytes and in EOMES^lo^ and EOMES^hi^ CD3^+^CD8SP thymocytes from Balb/c mice, shown as a reference. SPADE trees indicate cell frequency of CD3^+^CD8SP thymocytes from WT and *Eomes*^*Tg*^ mice using the tree constructed from WT Balb/c CD3^+^CD8SP thymocytes as shown in Fig. 1. **c** IFNγ expression measured by flow cytometry in CD3^+^CD8SP thymocytes from WT and *Eomes*^*Tg*^ mice cultured with IL-12+IL-18 for 16 h or with phorbol-myristate-acetate and ionomycin (P/I) for 4 h. For P/I, EOMES^lo^ and EOMES^hi^ CD3^+^CD8SP thymocytes from Balb/c mice are shown. **d** Volcano plot of RNA-seq data of CD3^+^CD8SP thymocytes from WT versus *Eomes*^*Tg*^ mice show the adjusted *P*-value versus fold-change (up in *Eomes*^*Tg*^, red; up in WT, green). The numbers of differentially expressed genes are indicated. **e** GSEA plots of innate memory (T_IM_)-specific (left) or naïve (N)-specific (right) gene sets in CD3^+^CD8SP thymocytes from *Eomes*^*Tg*^ mice. NES normalized enrichment score. **f** Expression heatmaps of RNA-seq data comparing mean fold-change (FC) of T_IM_ and *Eomes*^*Tg*^ CD3^+^CD8SP thymocytes in comparison to their respective naïve and WT controls, respectively. CPM counts per million. In **a**–**c** horizontal bars indicate median ± interquartile range and are representative of more than three experiments. Each point represents an individual mouse. Statistics were calculated using Mann–Whitney test. ns not significant, *P < 0.05; **P < 0.01; and ***P < 0.001

These analyses strongly support the role of EOMES in driving the epigenetic reprogramming that underlies the differentiation of CD8SP thymocytes toward a memory phenotype. In order to formally assess the direct role of EOMES in these processes, we developed a transgenic mouse line that overexpresses this transcription factor in developing thymocytes under the control of hCD2 regulatory elements^30^. These mice were backcrossed on C57Bl/6 background as T_IM_ cells represent a minor population in the thymus of this strain. EOMES levels were uniformly upregulated in DN, DP, CD4SP, and CD8SP thymocytes (Fig. 5a). Apart from higher CD8SP/CD4SP and DN/DP ratios, we did not observe major perturbations of T cell development in these *Eomes*^*Tg*^ mice. We then assessed the phenotype and activation status of CD3^+^CD8SP thymocytes from these mice under steady-state conditions. We also displayed EOMES^lo^ and EOMES^hi^ CD3^+^CD8SP thymocytes from Balb/c mice as references (Fig. 5b). We observed an upregulation of memory markers (CD44, CXCR3, Ly6C, CD27, BCL2), cytokine receptors (CD122, CD124) and T-BET, as well as a downregulation of cell-adhesion molecules (CD24, CD49d), resident memory cell marker (CD103), components of the TCR complex (TCRβ chain, CD3ε), and the transcription factor EGR2. Unsupervised analysis of these data showed that CD3^+^CD8SP thymocytes from WT and *Eomes*^*Tg*^ mice phenotypically resembled naïve and T_IM_ cells, respectively. Notably, ectopic expression of EOMES in CD4SP thymocytes did not induce CD44, Ly6C, or CD124 expression, suggesting that these effects are dependent on the cellular context (Supplementary Fig. 4). Functionally, the capacity of CD3^+^CD8SP thymocytes from *Eomes*^*Tg*^ mice to produce IFNγ in response to phorbol-myristate-acetate and ionomycin (P/I) as well as IL-12+IL-18 stimulations was significantly increased (Fig. 5c). They also showed a heightened responsiveness to IL-4 ex vivo stimulation despite similar pSTAT6 levels (Supplementary Fig. 5).

Next, we performed RNA-seq analysis of CD8SP thymocytes from *Eomes*^*Tg*^ and C57BL/6 control mice (sorting strategy, Supplementary Fig. 6). We identified 254 upregulated and 491 downregulated genes upon ectopic expression of EOMES (Fig. 5d, Supplementary Data 1). Globally, naïve-specific and T_IM_-specific genesets were significantly enriched in WT and *Eomes*^*Tg*^ CD8SP thymocytes, respectively (Fig. 5e). It encompassed genes involved in cytotoxicity, NK, or cytokines/chemokines receptors, and transcription factors. However, several of these signature genes (e.g. *Fas*, *Il18rap*, *GzmB*, *Klra7, Ccr2, Mef2a, Sox4*) were not differentially expressed in *Eomes*^*Tg*^ cells. Interestingly, *Runx3* and especially *Runx2* expressions were upregulated in *Eomes*^*Tg*^ mice, whereas only *Runx2* was upregulated in T_IM_. These discrepancies could be related to the fact that EOMES expression in *Eomes*^*Tg*^ mice did not reach the levels observed in T_IM_ cells from Balb/c mice (Fig. 5b) as we observed dose-dependent effects of EOMES on the expression of several markers when comparing WT, *Eomes*^*WT/Tg*^, and *Eomes*^*Tg/Tg*^ C57BL/6 mice (Supplementary Fig. 7). Despite these limitations, these data clearly indicate that ectopic EOMES expression in developing CD8SP thymocytes is sufficient to drive most phenotypic, functional, and transcriptional features of T_IM_ cells.

### EOMES induces epigenetic changes in enhancer regions

**Fig. 6:**
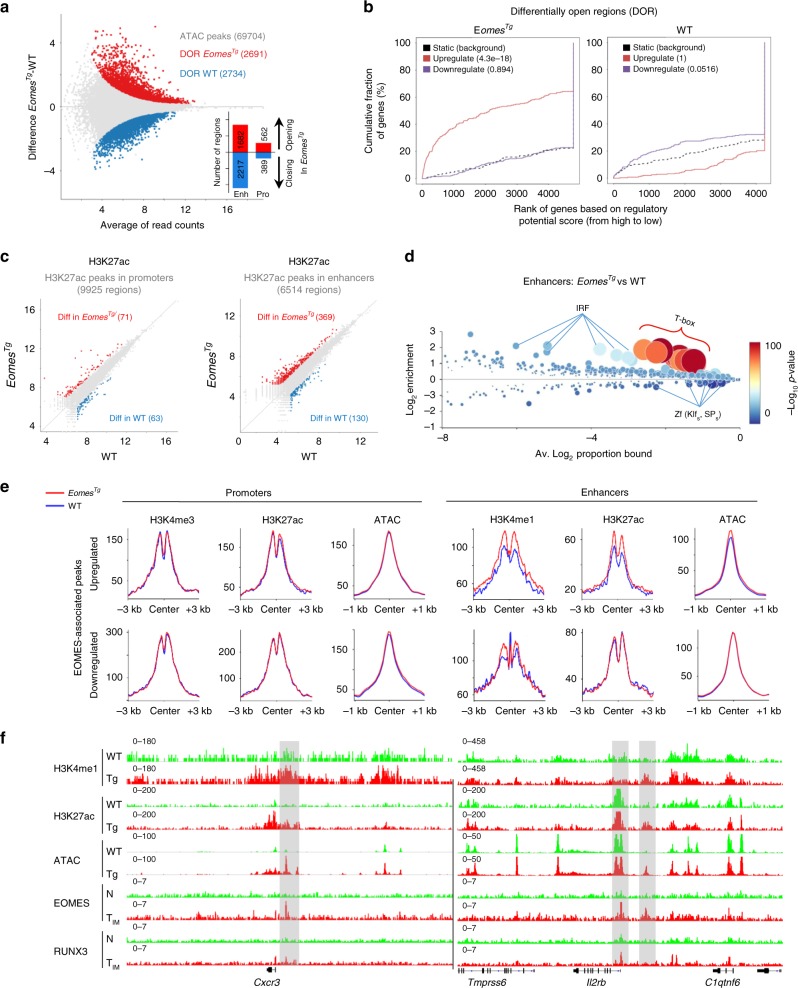
EOMES induces epigenetic changes in enhancer regions. **a** MA plot of mean ATAC-seq counts per peaks showing the differentially open regions (DOR) of CD8SP WT (blue) and *Eomes*^*Tg*^ (red) cells with the indicated number of regions. Histograms indicate the number of opening or closing regions in *Eomes*^*Tg*^ in comparison to WT cells at promoters and enhancers. **b** Cumulative distribution plot generated by BETA algorithm showing the predicted activating/repressive functions of DOR in CD8SP WT and *Eomes*^*Tg*^ with the indicated *P*-values determined by the Kolmogorov–Smirnov test. **c** Scatter plots display differentially active H3K27ac peaks at promoters (left) and enhancers (right) in CD8SP WT (blue) and *Eomes*^*Tg*^ (red) cells. **d** CiiiDER analysis for putative transcription factors motifs from DOR of CD8SP *Eomes*^*Tg*^ and WT at enhancers. Transcription factors are coloured according to their gene coverage *P*-value and whether they are over- (red) or under- (blue) represented. The size of each point is also proportional to log_10_ *P*-value. **e** Normalized coverage plot of histone modifications (H3K4me1, H3K4me3 and H3K27ac) and chromatin accessibility (ATAC-seq) centred on EOMES-binding sites at promoters and enhancers annotated to genes from Fig. 5e. **f** Representative ChIP-seq tracks of H3K4me1, H3K27ac, ATAC-seq, EOMES, and RUNX3 from the indicated population at the enhancers of *Cxcr3* and *Il2rb* loci highlighted in grey. ChIP-seq was performed on three independent IPs (*n* = 3 mice per sample). ATAC-seq was performed on two independent samples (*n* = 2 mice per sample) from each group

In order to define whether the acquisition of this EOMES-dependent transcriptional program was accompanied by epigenetic modifications, we first performed ATAC-seq profiling of WT or *Eomes*^*Tg*^ CD8SP thymocytes. Under the influence of EOMES, 5425 regions were found to be differentially accessible (Fig. 6a, Supplementary Data 3). Regulatory regions that were more accessible were clearly associated with genes that were induced in *Eomes*^*Tg*^ cells (Fig. 6b, Supplementary Data 3). In sharp contrast, peaks that were less accessible were not associated with downregulated genes (Fig. 6b), suggesting that distinct mechanisms are at play. In parallel, we observed that EOMES ectopic expression was also associated with the modulation of H3K27ac levels in regulatory regions (Fig. 6c, Supplementary Data 2). This was largely reflected by increased H3K27ac levels within enhancers rather than promoters of *Eomes*^*Tg*^ cells. More active enhancers were strongly enriched for T-box motifs (Fig. 6d). We compared the changes in chromatin accessibility that occur in enhancers of T_IM_ and *Eomes*^*Tg*^ CD8SP cells (Supplementary Fig. 8). We observed that about half of the DOR of T_IM_ cells displayed the same behavior in *Eomes*^*Tg*^ cells. A motif analysis in T_IM_-specific cluster 1 did not reveal enrichment for a unique transcription factor that could contribute to T_IM_ development independently of EOMES. Furthermore, the proportion of regions from clusters 1 and 2 that overlap with EOMES and RUNX3 ChIP-seq peaks was found to be comparable. Next, we assessed histone marks and chromatin accessibility in WT and *Eomes*^*Tg*^ cells around EOMES-binding sites located near the target genes that we identified in T_IM_ cells (as depicted in Fig. 4f, g). We observed clear changes for histone marks and ATAC signals in enhancer rather than promoter regions associated with upregulated EOMES target genes (Fig. 6e, f). Taken together, these data indicate that the recruitment of EOMES to distal regulatory elements drives local epigenetic changes that promote transcriptional activity.

### BRG1 is critically involved in EOMES-dependent program

**Fig. 7:**
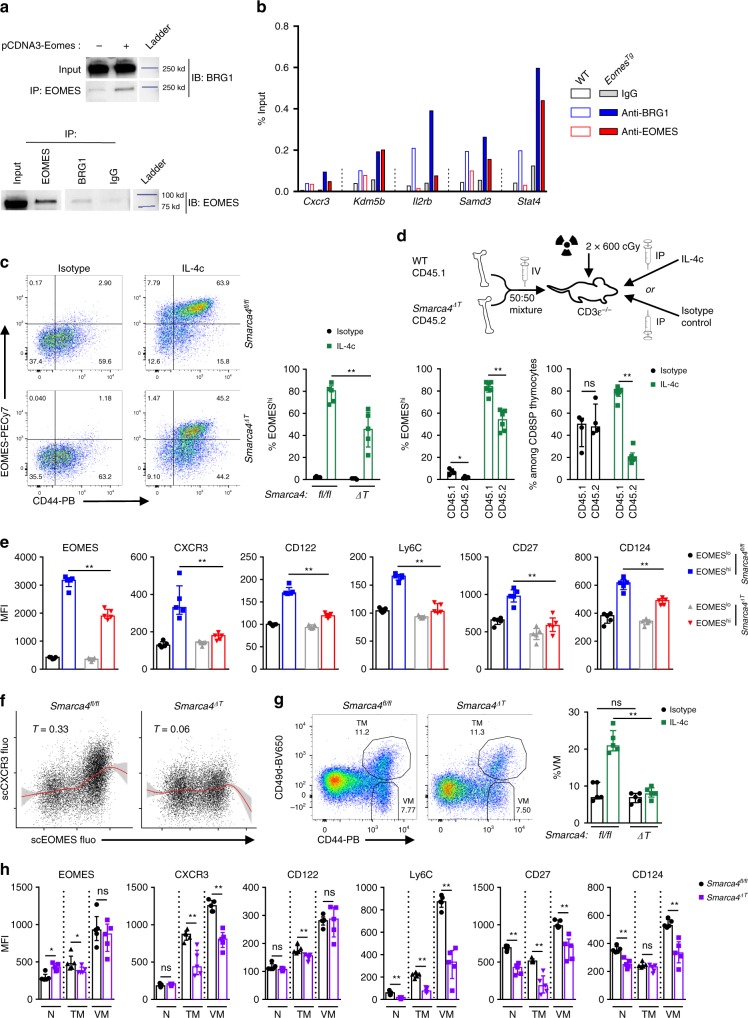
BRG1 is critical to induce EOMES-dependent program. **a** Immunoblot (IB) of lysates from 293 cells transfected with an Eomes vector and immunoprecipitated (IP) with an anti-EOMES (up) or anti-BRG1 (down) antibody. **b** ChIP experiment for EOMES and BRG1 from WT and *Eomes*^*Tg*^ CD3^+^CD8SP thymocytes followed by qPCR at the indicated loci. Each horizontal bar represents an individual data point from a single experiment. **c** Representative flow cytometry plots gated on CD3^+^CD8SP thymocytes from mice lacking *Smarca4* expression in T cells (*Smarca4*^*fl/fl*^xCD4Cre*: Smarca4*^*ΔT*^, down) and their littermate controls (*Smarca4*^*fl/fl*^, up) after 4 days of daily intra-peritoneal injection of anti-IL-4/rIL-4 (IL-4c) or an isotype control (left panel). Percentage of EOMES^hi^ CD3^+^CD8SP thymocytes after isotype control or IL-4c injections (right panel). **d** Mixed bone marrow chimera experimental scheme (upper panel). cGy centigrays. Percentages of EOMES^hi^ cells among WT vs. *Smarca4*^*ΔT*^ CD3^+^CD8SP thymocytes (lower left panel), and proportions of WT vs. *Smarca4*^*ΔT*^ cells among CD3^+^CD8SP thymocytes (lower right panel) after isotype control or IL-4c injections. **e** Expression (MFI) of cell markers in EOMES^lo^ and EOMES^hi^ CD3^+^CD8SP thymocytes from *Smarca4*^*fl/fl*^ vs. *Smarca4*^*ΔT*^ mice after IL-4c injections. **f** Representative correlation plots of EOMES and CXCR3 single-cell (sc) fluorescences in CD3^+^CD8SP thymocytes from *Smarca4*^*fl/fl*^ and *Smarca4*^*ΔT*^ mice after IL-4c injections. Kendall’s Tau correlation coefficients and Loess fitting curves (red lines) and their corresponding confidence interval (gray area) are shown. **g** Representative flow cytometry plots gated on CD8^+^ splenocytes from *Smarca4*^*fl/fl*^ and *Smarca4*^*ΔT*^ mice at steady-state, showing the proportions of CD44^hi^CD49d^lo^ (virtual memory cells, VM) and CD44^hi^CD49d^hi^ (true memory cells, TM) (left panel). VM proportions in CD8^+^ splenocytes from *Smarca4*^*ΔT*^ and *Smarca4*^*fl/fl*^ mice after isotype control or IL-4c injections are shown (right panel). **h** Expression (MFI) of cell markers in naïve (N), TM, and VM subpopulations of CD8^+^ splenocytes from *Smarca4*^*fl/fl*^ and *Smarca4*^*ΔT*^mice. In **c**, **d**, **e**, **g**, and **h**, horizontal bars indicate median ± interquartile range (each point represents a single mouse, *n* = 5 mice per group). Statistics were calculated using Mann–Whitney test. ns not significant, *P < 0.05, **P < 0.01. Source data are provided as a Source Data file. Source Data file

As we had identified members of the SWI/SNF family as potential partners of EOMES, we reasoned that this remodeling complex could participate to the EOMES-driven differentiation program. Previous work indicated that T-BET is able to interact with several epigenetic regulators, including JMJD3, UTX, and BRG1^31^. Furthermore, BRG1 was found to be required for optimal T-BET-induced and EOMES-induced *Ifng* expression in transient transfection experiments^31^. We therefore hypothesized that the SWI/SNF machinery could be involved in EOMES-induced T_IM_ development. We performed co-immunoprecipitation experiments in 293 cells transfected with an *Eomes* expression vector. As shown in Fig. 7a, BRG1 and EOMES co-precipitated, suggesting that these proteins can be found in the same molecular complexes. We further showed that EOMES binding to regulatory regions associated with *Il2rb*, *Cxcr3, Kdm5b, Samd3*, and *Stat4* loci in *Eomes*^*Tg*^ CD8SP thymocytes was accompanied by increased BRG1 recruitment (Fig. 7b). This result suggests that recruitment of BRG1-containing complexes along with EOMES could contribute to CD8SP thymocytes differentiation into T_IM_ cells. We therefore generated CD4Cre^+^ *Smarca4*^fl/fl^ (*Smarca4*^ΔT^) mice to assess the role of BRG1 in CD8SP thymocytes. As previously shown^32^, using this strategy, we did not observe a major perturbation of T cell development. We injected rIL-4/anti-IL-4 mAb complexes (IL-4c) to induce the development of EOMES^hi^ CD8SP thymocytes^33^. We performed this in vivo experiment in *Smarca4*^fl/fl^ (control) and *Smarca4*^ΔT^ mice (Fig. 7c), as well as in a mixed bone marrow chimera setting, where irradiated CD3ε^−/−^ mice received a 50:50 bone marrow mixture from WT CD45.1 and *Smarca4*^ΔT^ CD45.2 mice (Fig. 7d). This approach allowed us to mitigate any potential cell-extrinsic effect related to the impact of *Smarca4* loss in regulatory T cells^23^. In both experimental settings, upon IL-4c injection, we observed a strong increase in the proportion of CD44^hi^EOMES^hi^ CD8SP thymocytes in *Smarca4*^fl/fl^ controls and to a lesser extent in *Smarca4*^ΔT^ cells (Fig. 7c, d). Notably, we observed a disequilibrium between WT and *Smarca4*^ΔT^ cells in mixed BM chimeras upon IL-4c injection but not in control conditions (Fig. 7d). We looked at the expression of EOMES-dependent markers in EOMES^hi^ cells from both groups (Fig. 7e, Supplementary Fig. 9a). In both experimental settings, upregulation of CXCR3, CD122, Ly6C, and CD27 was found to be strongly BRG1-dependent. Induction of CD124 was partially inhibited in the absence of BRG1. As EOMES levels were significantly decreased among *Smarca4*^ΔT^ EOMES^hi^ cells from IL-4c-injected mice compared to controls, we exctracted the single-cell (sc) fluorescence intensities of EOMES and its targets. The sc levels of EOMES and these targets were significantly less correlated in *Smarca4*^ΔT^ cells compared to *Smarca4*^fl/fl^ controls (Fig. 7f, Supplementary Fig. 9b). In order to define whether the absence of BRG1 could have an impact on memory cells outside the thymus, we phenotyped the CD8^+^ T cell compartment in the spleen (Fig. 7g). The proportion of CD44^hi^CD49d^lo^ (virtual memory; T_VM_)^22^ and CD44^hi^CD49^hi^ (true conventional memory; T_M_) among CD8^+^ T cells was comparable in both groups under steady-state conditions. We observed a clear expansion of T_VM_ cells upon injection of IL-4c that was entirely dependent on BRG1. Finally, we assessed the expression of several memory markers among naïve, T_M_ and T_VM_ cells under steady-state conditions. Although T_VM_ cells in both groups expressed comparable levels of EOMES, the levels of CXCR3, Ly6C, CD27, and CD124 were found to be decreased in BRG1-deficient cells (Fig. 7h). Notably, expression levels of CXCR3, Ly6C, and CD27 were also decreased in BRG1-deficient T_M_ cells, suggesting that its role is not restricted to unconventional memory formation. Taken together, these results clearly indicate that BRG1 contributes to the acquisition of classical memory features by CD8^+^ T cells under steady-state conditions and upon in vivo injection of IL-4c.

## Discussion

Memory is a central paradigm of immunology. Because of the dynamic nature of Ag-driven T cell expansion/contraction and the different fate of each single cell, it is extremely hard to distinguish the processes that are only involved in the initial activation and differentiation of naïve cells into effector cells, from those that are responsible for the survival of a minor fraction of activated cells that subsequently acquire memory functions. Dozens of transcription factors and epigenetic modifiers potentially contribute to fate decision and programming during this process^34,35^. Herein, we show that the ectopic expression of a single transcription factor, EOMES, is sufficient to drive the acquisition of memory-associated phenotypical, transcriptional, and epigenetic profiles in developing CD8SP thymocytes. However, not every aspects of the process that takes place during the physiological development of cytokine-driven innate memory (T_IM_) cells were recapitulated in *Eomes*^*Tg*^ cells. We further show that the overexpression of EOMES increases the responsiveness to IL-4, thereby initiating a feed-forward loop. It would be important to formally examine the impact of *Eomes* deficiency on these different features.

We show that differentiation into T_IM_ cells is accompanied by extensive epigenetic modifications. Increased enhancer activity in T_IM_ cells was found to be the consequence of EOMES recruitment. A comparable process also takes place to a large extent in conventional memory (T_M_) cells. However, a significant proportion of T_M_-specific active enhancers, enriched for binding sites of TCR-induced transcription factors, such as IRF4, BATF, and NFATc1, was not observed in T_IM_ cells. Thus, despite the acquisition of memory markers and a transcriptomic profile closely related to T_M_ cells, the EOMES-driven differentiation into T_IM_ cells only partially recapitulates what occurs at the epigenetic level during Ag-dependent memory formation.

Among partners that potentially interact with EOMES, we identified transcription factors involved in T cell development, such as RUNX3. As recently suggested for conventional memory formation^36^, we show that in T_IM_ cells, EOMES was preferentially recruited to enhancer regions that were already bound by RUNX3 in naïve CD8SP cells. In addition, we observed a T_IM_-specific recruitment of RUNX3 at several locations around the *Eomes* locus (Supplementary Fig. 3), suggesting that RUNX3 could participate to EOMES upregulation in this setting as described in polyclonally activated CD8^+^ T cells^19^. Two recent reports indicate that RUNX3 might act as a pioneer factor very early during memory commitment^36,37^. Our results indicate that this could be the case even in the absence of TCR stimulation. RUNX3 deposition in naïve cells could establish a favourable chromatin environment for the subsequent recruitment of EOMES. Along this line, we observed that the ectopic expression of EOMES in CD4SP thymocytes, that express low levels of RUNX3, had different functional consequences than in CD8SP thymocytes (Supplementary Fig. 4). Furthermore, context-dependent interactions with other transcription factors could underlie the distinct roles of EOMES in memory vs. exhausted T cells^38^.

T-Box factors play a key role in early embryonic cell fate decisions and many aspects of organogenesis^39^. It is therefore not surprising that the interaction with histone-modifying enzymes represents a key feature of this family^40^. We show that EOMES shapes the enhancer landscape of T_IM_ cells and co-immunoprecipitates with members of the SWI/SNF machinery, histone deacetylases and their associated DNA-binding ATPase CHD4. In addition, the acquisition of several EOMES-dependent memory features was found to be strongly dependent on BRG1. This chromatin-remodeling factor is essential for modulating H3K27ac levels at distal enhancers^41,42^. Also, BRG1 could act upstream of EOMES by regulating its expression and/or recruitment. Our results strongly suggest that EOMES facilitates the recruitment of the SWI/SNF machinery to specific cis-regulatory elements that control the long-term commitment toward the T_IM_ cellular identity. Notably, similar molecular processes seem to be operational in T_M_ cells.

In summary, we provide evidence that although T_IM_ cells display typical characteristics of memory cells, they represent a distinct lineage that only partially recapitulates the conventional memory differentiation. The ectopic expression of EOMES in developing CD8SP thymocytes is sufficient to drive this unconventional memory program and acts in a BRG1-dependent fashion. This work broadens our view of the mechanisms that dictate CD8^+^ T cell fate by establishing the basis of the epigenetic reprogramming that underlie unconventional memory formation.

## Methods

### Mice

All experiments were performed on age-matched (from 8 to 12 weeks of age) female mice. Wild-type Balb/c and C57Bl/6 mice were purchased from Envigo. *Il4*^*−/−*^ and *Stat6*^*−/−*^ mice under Balb/c background were purchased from the Jackson Lab. *Irf9*^*−/−*^ mice under Balb/c background were purchased from Riken BioResearch Center. CD3ε^−/−^ and CD45.1 mice under C57Bl/6 background were obtained from the Jackson Lab. *Smarca4*^fl/fl^ mice under C57BL/6 background were kindly provided by Professor Pierre Chambon, GIE-CERBM (IGBMC)^43^ and were crossed to CD4Cre^+^ mice obtained from the Jackson Lab (Tg(Cd4-cre)1Cwi/BfluJ, Stock 017336) to generate *Smarca4*^ΔT^ mice. In order to generate *Eomes* transgenic mice, a 2.1 kB full-length cDNA encoding mouse EOMES was inserted into the VA-CD2 cassette containing the upstream gene regulatory region and locus control region of the hCD2 gene^30^. This construct was linearized and injected into BDF1 fertilized eggs to generate *Eomes*^*Tg*^ mice. Mice were backcrossed for six generations onto C57Bl/6 strain and then intercrossed in order to obtain homozygote transgenic mice (i.e. the de facto *Eomes*^*Tg*^ mice). These mice are available from the authors upon request. All animal work was carried out in compliance with and after approval by the institutional Animal Care and local committee for Animal Welfare from the Biopark ULB Charleroi (BUC).

### Cell preparation

For all experiments, thymi and spleens were dissected and further processed under sterile conditions. Single-cell suspensions were obtained in RPMI 1640 with 10% (vol/vol) fetal calf serum (FCS), 2 mM l-glutamine, 1 mM sodium pyruvate, 0.1 mM non-essential amino acids, 40 mM β-mercaptoethanol, 100 U ml^−1^ of penicillin, and 100 U ml^−1^ of streptomycin (all reagents from Lonza; solution hereafter referred to as complete medium).

### Cell sorting

CD8^+^ T cells were purified by negative selection (Dynabeads Untouched Mouse CD8 Cells Kit, Life Technologies) from thymocytes resuspended in phosphate buffered saline (PBS, Lonza) with 2% (vol/vol) FCS (Lonza) and 2 mM ethylenediaminetetraacetic acid (EDTA, Sigma). Untouched cells were stained to exclude dead cells and incubated with Fc receptor-blocking antibodies and a surface staining antibody mix (see the section “Flow cytometry”). Cells were sorted on a BD FACSAria™ III using fluorescence minus one (FMO) controls (see Supplementary Figs. 2 and 6 for gating strategies).

### Cell cultures and **ex vivo** stimulations

All cell cultures were performed in complete medium (see the section “Cell preparation”) at 37 °C and 5% CO_2_. Thymocytes were cultured for 4 h in the presence or absence of phorbol-myristate-acetate (50 ng ml^−1^, Sigma) and ionomycin calcium salt (1 µg ml^−1^, Sigma), or for 16 h in the presence or absence of recombinant murine (rm)IL-12 p70 (5 ng ml^−1^, Peprotech) and rmIL-18 (10 ng ml^−1^, MBL), brefeldin A (10 µg ml^−1^, Sigma) being added for the last 3 h before staining. In other experiments, thymocytes were cultured in the presence or absence of rmIL-4 (Peprotech) for 72 h at 20 ng ml^−1^ before staining, or for 30 min at 30 ng ml^−1^ before phospho-STAT6 staining.

### Administration of IL-4 complex **in vivo**

Carrier-free rmIL-4 (1.5 µg/mouse, Peprotech) was mixed in PBS (200 µl/mouse) with neutralizing anti-mouse IL-4 antibodies (50 µg/mouse, Bio × Cell, clone 11B11, catalog number BE0045). Control mice received only an anti-trinitrophenol Rat IgG1 isotype control (50 µg/mouse, Bio × Cell, clone TNP6A7, catalog number BE0290). Mice were injected daily for 4 days intraperitoneally before harvesting thymi and spleens.

### Mixed bone marrow chimera

CD3ε^−/−^ mice were irradiated twice at 600 centigrays (cGy) before being injected intravenously with a 50:50 bone marrow mixture from WT CD45.1 and *Smarca4*^ΔT^ CD45.2 mice (Fig. 7d). Reconstitution was achieved at 6 weeks post-irradiation based on peripheral blood examination and IL-4c administration was started 10 weeks post-irradiation.

### Flow cytometry

Single-cell suspensions were stained to exclude dead cells (LIVE/DEAD™ Fixable Aqua Dead Cell Stain Kit, for 405 nm excitation, Life Technologies), and incubated with rat anti-mouse CD16/CD32 (BD, clone 2.4G2, dilution 1:100, catalog number 553141) and a surface antibody mix prepared in brilliant stain buffer (BD) for 20 min at 4 °C in the dark. Except for sorting, cells were fixed and permeabilized for 30 min at 4 °C in the dark (eBioscience™ Foxp3/Transcription Factor Staining Buffer Set, Life Technologies) before intranuclear/intracytoplasmic staining (30 min at 4 °C in the dark). For phospho-STAT6 staining, we adapted the harsh alcohol method (BD Phosflow protocol III). Flow cytometry data were acquired on a BD LSRFortessa™ cell analyzer after successfully running Cytometer Setup and Tracking beads (BD) and using the application settings featured on FACSDiva™ software (v 6.2). Data were analyzed using FlowJo v10 software (Tree Star) and the spanning-tree analysis tool SPADE V3.0 (http://pengqiu.gatech.edu/software/SPADE/)^21^.

Cells were labeled with antibodies against the following molecules (fluorochrome[s]; clone; dilution[s]; catalog number[s]): BCL2 (PE; 3F11; 1:10; 556537), CD3 (BV711; 145-2C11; 1:100; 563123), CD4 (AF700, APC-Cy7, PE-Cy7; RM4-5; 1:100; 561025, 565650, 561099), CD5 (BV421; 53-7.3; 1:100; 562739), CD8 (PerCP, PB; 53-6.7; 1:50; 561092, 558106), CD11c (APC-Cy7; HL3; 1:100; 561241); CD19 (APC-Cy7; 1D3; 1:100; 561737), CD24 (BV605, PE; M1/69; 1:100; 563060, 553262), CD25 (APC; PC61; 1:50; 561048), CD27 (BV786; LG.3A10; 1:100; 740890), CD44 (APC, FITC, V450; IM7; 1:50; 561862, 561859, 560451), CD49d (BV650, PE; R1-2; 1:200, 1:50; 740458, 553157), CD62L (BV786; MEL-14; 1:100; 564109), CD69 (BV786; H1.2F3; 1:100; 564683), CD103 (BV421; M290; 1:100; 562771), CD122 (FITC; TM-β1; 1:50; 553361), CD124 (BV421, PE; mIL4R-M1; 1:100; 564086, 552509), CD127 (BV786, PE-Cy7; SB/199; 1:100, 1:50; 563748, 560733), CXCR3 (APC; CXCR3-173; 1:50; 562266), GZMB (AF647; GB11; 1:100; 560212), IFNγ (BV786; XMG1.2; 1:200; 563773), Ki67 (AF700; B56; 1:50; 561277), Ly6C (BV605, FITC; AL-21; 1:50; 563011, 553104), Ly6C and Ly6G (APC-Cy7; RB6-8C5; 1:100; 557661), NK1.1 (APC-Cy7; PK136; 1:100; 560618), STAT6 (pY641) (AF488; J71-773.58.11; 2:3; 558243), TCRβ (BV605, FITC; H57-597; 1:100; 562840, 553170), all from BD; EGR2 (PE; erongr2; 1:50; 12-6691-82), EOMES (eFluor 660, PE, PE-Cy7; Dan11mag; 1:100; 50-4875-82, 12-4875-82, 25-4875-82), T-BET (eFluor 660, PE, PE-Cy7; eBio4B10; 1:100; 50-5825-82, 12-5825-82, 25-5825-82), all from eBioscience; CD69 (BV605; H1.2F3; 1:50; 104530), from Biolegend.

Sequential gating strategies for FACS data are provided in Supplementary Figs. 10 and 11.

### RNA purification and RNA-seq

RNA from 10^6^ sorted CD8SP populations (in triplicates) was extracted using RNeasy Plus Mini kit according to manufacturer’s instructions (Qiagen), and sample quality was tested on a 2100 Bioanalyzer (Agilent). Total RNA from naïve and T_IM_ population was subject to single-end sequencing (30 × 10^6^ reads/sample, NextSeq platform) performed by Nucleomics Core, Belgium (www.nucleomics.be), whereas CD8SP population mRNA from WT and *Eomes*^*Tg*^ mice underwent paired-end sequencing (25 × 10^6^ reads/sample, Novaseq platform) performed by BRIGHTcore ULB-VUB, Belgium (http://www.brightcore.be). For single-end sequencing, obtained reads were filtered using FastX 0.0.13 and ShortRead 1.16.3, polyA-reads (more than 90% of the bases equal A), ambiguous reads (containing N), low-quality reads (more than 50% of the bases < Q25), and artifact reads (all but three bases in the read equal one base type) were removed. Reads are then aligned to the reference genome mm10 using Tophat v2.0.13 with the following parameters: –library-type fr-firststrand –min-intron-length 50 –max-intron-length 500000 –no-coverage-search –no-mixed –read-realign-edit-dist 3. We removed reads from the alignment that are non-primary mappings or have a mapping quality ≤ 20. We sorted the reads from the alignment according to chromosomes and indexed the resulting BAM-files. Read counts in the alignment BAM-files that overlapped with the gene features were obtained using featureCounts v1.4.6 with the following parameters: -Q 0 -s 2 -t exon -g gene_id and genes that have less than 1 counts-per-million were removed. Raw counts were normalised with the EDASeq package and FPKM values were retreived. We used edgeR 3.4.0 package to perform differential expression analysis by applying a corrected *P*-value FDR < 0.05 and absolute log_2_-ratio larger than 1. These lists were used as gene sets to test the statistical enrichment in the different datasets mentioned in Fig. 1f, using the BubbleGUM software^44^.

### ATAC-seq

For library preparation^45^, nuclei from 20,000 (Naïve/T_IM_) or 50,000 sorted cells (WT/*Eomes*^*Tg*^) were subject to transposition reaction in 1x TD buffer containing 2.5 µl transposase Nextera enzyme (Nextera DNA sample prep kit, Illumina) and DNA was purified using MinElute PCR Purification Kit (Qiagen). Purified DNA was amplified by PCR using NEBNext High-Fidelity 2 × PCR Master Mix (New England Biolabs) with 10–12 cycles. Amplified libraries were purified using MinElute PCR Purification Kit (Qiagen) and quality controlled using a Bioanalyzer High-Sensitivity DNA Analysis kit (Agilent). Paired-end sequencing was performed on NovaSeq or NextSeq 500 platforms (Illumina).

Paired-end reads were mapped to mouse genome mm10 with Bowtie2^46,47^ using default parameters for paired-end reads. Reads that mapped several regions, or with insufficient mapping quality, were removed with samtools view. We also removed reads located within the blacklist of the ENCODE project^48^. Duplicate reads were removed with MarkDuplicates tools (Picard suite). When required, we generated pseudoreplicates as described in the ATAC-seq pipeline ENCODE document. Peaks were called with MACS2^49^ using the following parameters: -f BAMPE -g mm -q 0.05 –nomodel –call-summits -B –SPMR.

Regions obtained from naïve, T_IM_ and CD8SP WT and *Eomes*^*Tg*^ populations by MACS2 were subjected to differential analysis using SeqMonk 1.43.0 (Mapped Sequence Analysis Tool, Babraham Bioinformatics, http://www.bioinformatics.babraham.ac.uk/projects/seqmonk/). First, we created an atlas containing all obtained peaks for all the populations using bedtools^50^ with a minimum overlap of 1 bp. We applied two filters: DESeq2^51^ with a *p*-adjusted cutoff of 0.05, and the proportion of library filter with a *p*-adjusted cutoff of 0.05 for naïve and T_IM_ populations, and 0.001 for WT and *Eomes*^*Tg*^ populations. Resulting peaks were separated into two categories: peaks located in promoters (located within 2 kb around TSS) and peaks located in enhancers (not located in the defined promoter regions). For CD8SP WT and *Eomes*^*Tg*^ populations, we submitted the differentially accessible regions to the BETA package^27^ using default parameters. For downstream visualisation, a scaling factor was calculated using deepTools package^52^ to normalize peak intensity to FRiP (fraction of reads in peaks) and generate bigwig files.

For Gene ontology analysis, we introduced BED files resulting from the overlap between H3K27ac clusters and ATAC-seq peaks atlas to the GREAT tool with default parameters^25^. Ciiider algorithm (http://ciiider.com/) was used to perform motif enrichment in the differentially accessible regions of naïve, T_IM_, CD8SP WT, and *Eomes*^*Tg*^ populations in enhancers. An atlas containing accessible regions obtained from Scott-Brown et al. (GSE88987)^4^ and naïve and T_IM_ population was intersected with H3K27ac clusters in Fig. 3a to perform motif-enrichment analyses using the AME tool from meme-suit^53,54^ with default parameters. To analyze the positional distribution and the best combined match of EOMES and RUNX3 in a set of sequences, we used CentriMo and MAST^55,56^ with default parameters.

### ChIP-seq

For sample preparation, CD8SP from pooled thymus were isolated and crosslinked with formaldehyde 1% for 10 min at room temperature before staining. Crosslinking was stopped with 0.125 M glycine. To immunoprecipitate BRG1, disuccinimidyl glutarate (DSG) (ThermoFisher scientific) at 2 µM was used for 40 min followed by formaldehyde 1% for 10 min at room temperature. Cells were then washed twice with ice-cold PBS and sorted. Each sorted cell population was resuspended with ChIP-lysis buffer (10 mM Tris pH8, 140 mM NaCl, 1 mM EDTA, 1% Triton x-100, 0.5% SDS, 0.1% Na-Deoxycholate) supplemented with protease inhibitor cocktail (PIC) for 10 min on ice, then sheared using a bioruptor device (Diagenode) to obtain a fragments size range between 200 and 600 bp. After clearance by centrifugation at 4 °C, sheared chromatin resulting from 3 × 10^5^ to 5 × 10^5^ cells were used for each immunoprecipitation of histone marks H3K4me3 (1 µg, Merck, catalog number 17-614), H3K27ac (1 µg, Abcam, catalog number ab4729), H3K4me1 (1 µg, Abcam, catalog number ab8895) or normal rabbit IgG control (1 µg, Merck, catalog number 12-370), and 2 × 10^6^ cells were used for the transcription factors EOMES (4 µg, Abcam, catalog number ab23345), RUNX3 (dilution 1:250, provided by Prof. Yoram Groner), and BRG1 (2–4 µg, Abcam, catalog number ab110641). Protein G magnetic-activated beads (Active Motif, catalog number 53034), and target antibody together with dilution buffer (20 mM Tris pH 8, 2 mM EDTA, 150 mM NaCl, 1% Triton x-100) were added to the chromatin and incubated overnight at 4 °C. Each IP was washed five times using the following buffers:

Once with washing buffer 1 (20 mM Tris pH 8, 2 mM EDTA, 1% Triton x-100, 0.1% SDS, 150 mM Nacl), once with washing buffer 2 (20 mM Tris pH 8, 2 mM EDTA, 1% Triton x-100, 0.1% SDS, 500 mM Nacl), once with washing buffer 3 (20 mM Tris pH 8, 1 mM EDTA, 0.5% NP40, 0.5% Na-Deoxycholate, 250 mM LiCl), and twice with washing buffer 4 (10 mM Tris pH8, 1 mM EDTA). Chromatin–antibody complexes were eluted with freshly prepared elution buffer (1% SDS, 100 mM NaHCO_3_). Eluted chromatin was incubated with NaCl (at a final concentration of 200 mM) for 4 h at 65 °C for reverse crosslinking and treated with RNAse and Proteinase K for 1 h at 45 °C. DNA was then purified using the MinElute PCR Purification Kit according to the manufacturer’s instructions (Qiagen). We isolated 1% of total sheared chromatin which underwent all steps of ChIP-seq protocol, except IP and washing steps, and used it as a control for background noise. Before sequencing, each individual ChIP was tested for positive and negative controls by qPCR, and we pooled three ChIPs for the same histone mark of the same sorted population. Primers used for qPCR are provided in Supplementary Data 5. Library preparation was performed for histone modifications (3–6 ng) and transcription factors (0.5 ng) using TruSeq ChIP library Preparation kit (Illumina IP-202-1012) and MicroPlex Library Preparation kit (Diagenode C05010013), respectively, following manufacturer’s instructions. Paired-end sequencing was performed on NextSeq 500 (Illumina) at the EPICS platform (http://epics.ulb.be/).

For mapping and peak-calling, single-end reads were mapped to mouse genome mm10 with Bowtie2^46,47^ using default parameters for single-end reads. Reads that mapped several regions, or with insufficient mapping quality, were removed with samtools view. We also removed reads located within the blacklist of the ENCODE project^48^. Duplicate reads were removed with MarkDuplicates tools (Picard suite). Peaks were then called with the callpeak tool from MACS2^49^ with a *q*-value of 0.01 for H3K4me3, H3K4me1, and H3K27ac, a *P*-value of 0.01 for RUNX3, and with a *P*-value of 0.001 for EOMES. BATF, IRF4, JUNB, JUND, and cJUN (GSE54191)^26^, TCF1 (GSM1889262)^57^, and FOXO1 (GSM1141667)^58^ ChIP-seq data were downloaded from NCBI SRA database as fastq files and mapped to the mouse genome mm10 using Bowtie2 offered by the Galaxy platform^59^ in a sensitive local preset parameters. Peaks were called using MACS2 with the following parameters: –format = BAM –gsize 2730871774 –bw 300 –mfold 5 50 –qvalue 0.05. Resulting peaks were intersected with H3K27ac clusters to calculate the percentage of overlap.

We performed differential analyses on histone marks’ regions with custom scripts. First, we created an atlas of promoters or enhancers containing all obtained peaks for all the populations using bedtools^50^ with a minimum overlap of 1 bp. Read counts were calculated for each region with featureCount tool^60^. We then performed a normalization of reads to lowest total library size and calculated a log_2_FoldChange and a *P*-value according to Poisson distribution. Finally, *P*-values were adjusted using built-in R “FDR” method (https://www.R-project.org/). Promoters were defined as H3K4me3^+^ regions located between −5 and +2 kb from the transcription start sites (TSS). Enhancers were defined as H3K4me1^+^ regions located at least 2 kb away from the TSS and that do not overlap with H3K4me3^+^ promoters. To assess the activity of these regions, we generated a H3K27ac peak atlas (29,414 regions) which was then overlapped with enhancer or promoter regions atlases.

### RIME

For Rapid Immunoprecipitation Mass spectrometry of Endogenous proteins (RIME), CD8+ T cells were isolated from spleens of WT Balb/c mice and stimulated for 3 days by plate-bound anti-CD3 (BD, clone 145-2C11, 5 mg ml^−1^, catalog number 557306) and soluble anti-CD28 (BD, clone 37.51, 1 mg ml^−1^, catalog number 553294) in the presence of recombinant human (r)IL-2 (10 ng ml^−1^, R&D). Cells were washed and further expanded with rIL-2 for 3 days. 5 × 10^7^ cells were fixed with 1% methanol-free formaldehyde for 8 min and quenched with 0.125 M glycine. Chromatin was isolated by the addition of lysis buffer, followed by disruption with a Dounce homogenizer. Lysates were sonicated and the DNA sheared to an average length of 300–500 bp. An aliquot of chromatin (150 µg) was precleared with protein G agarose beads (Invitrogen). Proteins of interest were immunoprecipitated using 15 µg of anti-EOMES or control IgG (same as ChIP experiments) and protein G magnetic beads. Protein complexes were washed and treated with trypsin. Protein digests were separated from the beads and purified using a C18 spin column (Harvard Apparatus). The peptides were vacuum dried and analyzed by LC–MS/MS on a Thermo Scientific Q Exactive Orbitrap Mass spectrometer in conjunction with a Proxeon Easy-nLC II HPLC (Thermo Scientific) and Proxeon nanospray source. The digested peptides were loaded on a 100 micron × 25 mm Magic C18 100 Å 5U reverse phase trap where they were desalted online before being separated using a 75 micron × 150 mm Magic C18 200 Å 3 U reverse phase column. Peptides were eluted using a 90 min gradient with a flow rate of 300 nl/min. An MS survey scan was obtained for the *m*/*z* range 300–1600, MS/MS spectra were acquired using a top 15 method, where the top 15 ions in the MS spectra were subjected to high-energy collisional dissociation (HCD). An isolation mass window of 1.6*m*/*z* was for the precursor ion selection, and normalized collision energy of 27% was used for fragmentation. A 5 s duration was used for the dynamic exclusion.

All MS/MS samples were analyzed using X! Tandem (The GPM, thegpm.org; version CYCLONE (2013.02.01.1)). X! Tandem was set up to search the mouse_pr_20160108_Zf0QxA database (unknown version, 99854 entries), the cRAP database of common laboratory contaminants (www.thegpm.org/crap; 114 entries) plus an equal number of reverse protein sequences assuming the digestion enzyme trypsin. X! Tandem was searched with a fragment ion mass tolerance of 20 ppm and a parent ion tolerance of 20 ppm. Carbamidomethyl of cysteine was specified in X! Tandem as a fixed modification. Glu→pyro-Glu of the n-terminus, ammonia-loss of the n-terminus, gln→pyro-Glu of the n-terminus, deamidated of asparagine and glutamine, oxidation of methionine and tryptophan, dioxidation of methionine and tryptophan and acetyl of the n-terminus were specified in X! Tandem as variable modifications. Scaffold (version Scaffold_4.4.8, Proteome Software Inc., Portland, OR) was used to validate MS/MS-based peptide and protein identifications. Peptide identifications were accepted if they exceeded −log (Expect Scores) scores of >1.5 and if they contained at least one identified peptide. Proteins that contained similar peptides and could not be differentiated based on MS/MS analysis alone were grouped to satisfy the principles of parsimony.

### Co-immunoprecipitation experiments

Two-hundred and ninety-three cells (ATCC Cat#CRL-1573) were transiently transfected with pcDNA3-mEOMES-V5 expression plasmids and cultured in DMEM media. Cells were lysed in cold lysis buffer (50 mM Tris–HCl pH 7.4, 1% NP-40, 0.25% Na-deoxycholate, 120 mM NaCl, 1 mM EDTA, 1X Protease inhibitor cocktail). Protein G magnetic-activated beads (Active Motif) were added to the cell lysate to preclear and reduce non-specific interactions between proteins and antibodies. The cleared cell lysate together with dilution buffer (50 mM Tris–HCl pH 7.4, 120 mM NaCl, 1 mM EDTA) and of primary antibodies for detection of BRG1 or EOMES (same as ChIP experiment) were incubated overnight at 4 °C. Protein G magnetic-activated beads (Active Motif) were added to the protein–antibody complex and incubated for 1 h at room temperature. Beads were then washed three times with precooled dilution buffer and the lysate was eluted using the elution buffer (130 mM Tris pH 6.8, 4% SDS, 0.02% bromophenol blue, 100 mM DTT, 20% glycerol). The resulting eluate was boiled at 100 °C for 5 min before being loaded into 4–15% precast polyacrylamide gel to separate proteins, and wet-transferred onto polyvinylidene fluoride (PVDF) membranes. After being blocked in 5% nonfat milk for 1 h at room temperature, the membrane was incubated at 4 °C overnight with the addition of the following primary antibodies of BRG1 (dilution 1/3000) or EOMES (dilution 1/1500). After washing, the membrane was incubated with the secondary antibody for 1 h at room temperature followed by extensive washing. The protein signal was detected by chemiluminescence (Lumigen ECL Ultra, TMA-6). Uncropped/unprocessed scans corresponding to the blots shown in Fig. 7a are available in Source Data file.

### Statistical analyses

Prism 6.0 was used for statistical analysis. Mann–Whitney test was used to compare two data sets. Wilcoxon matched-pairs signed rank test was used to compare the relative expressions of cluster-associated genes between conventional memory (T_M_/N) and innate memory CD8SP thymocytes (T_IM_/N). For correlation between single-cell fluorescence of EOMES and innate memory markers, R and Rkward (https://rkward.kde.org) were used to compute Kendall’s tau (single nonlinear correlation) and Fisher *Z* transformation (comparison of two independent correlations). For all analyses, no data points were excluded.

### Reporting summary

Further information on research design is available in the Nature Research Reporting Summary linked to this article.

## Supplementary information

Supplementary Information

Peer Review

Reporting Summary

Description of Additional Supplementary Files

Supplementary Data 1

Supplementary Data 2

Supplementary Data 3

Supplementary Data 4

Supplementary Data 5

source data

## Data Availability

A reporting summary for this Article is available as a Supplementary Information file. RNA-seq, ChIP-seq and ATAC-seq data that support the findings reported in this study have been deposited in the GEO Repository with the accession code GSE124914. The mass spectrometry proteomics data corresponding to Fig. 4a have been deposited to the ProteomeXchange Consortium via the PRIDE^61^ partner repository with the dataset identifier PXD014142 and 10.6019/PXD014142. The source data underlying Figs. 3b and 7a are provided as a Source Data file. All data is available from the corresponding author upon reasonable request.
