# Supplementary material for: EOMES interacts with RUNX3 and BRG1 to promote innate memory cell formation through epigenetic reprogramming
Source: Nat Commun. 2019 Jul 24;10:3306. doi: 10.1038/s41467-019-11233-6 (PMC6656725; doi:10.1038/s41467-019-11233-6)
Supplement: Supplementary file 1 — Supplementary Information [file 41467_2019_11233_MOESM1_ESM.pdf]

Supplementary information

EOMES interacts with RUNX3 and BRG1 to promote innate memory cell formation through epigenetic reprogramming

Istaces et al.

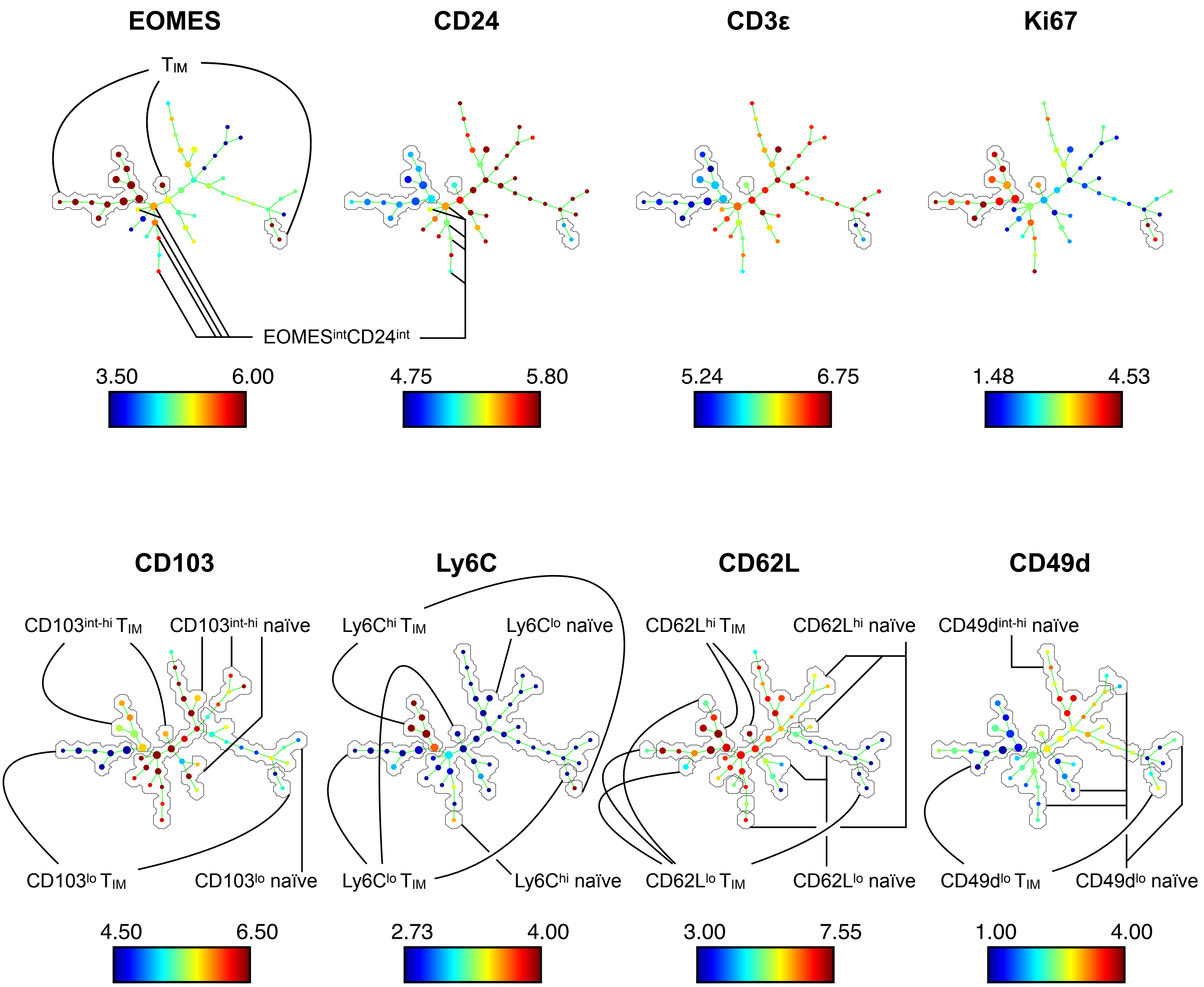

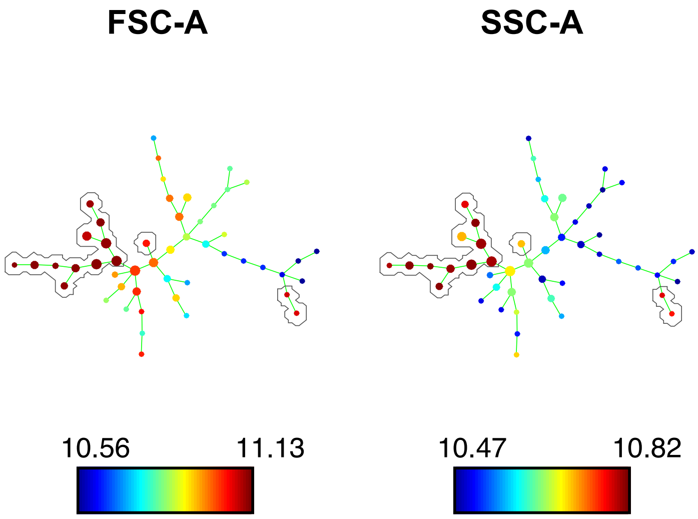

Supplementary Figure 1. SPADE of flow cytometry data gated on CD3$^+$CD8SP thymocytes from WT Balb/c mice. SPADE trees similar to the ones displayed in **Fig. 1b** showing the expression of other markers within cell nodes. Annotations for T_{IM} and for naïve cells are indicated using curved or straight lines, respectively. **a**, Some markers show a pattern of expression matching closely EOMES levels: CD24 and CD3ε levels decrease whereas Ki67 levels increase as EOMES levels increase. EOMES^{int}CD24^{int} clusters are likely to be cells actively transitioning from naïve (EOMES^{lo}) to T_{IM} (EOMES^{hi}) cells. **b**, Some markers show bimodal expression in naïve and/or T_{IM} cells. **c**, T_{IM} cells appear larger and show a more complex granularity than naïve cells.

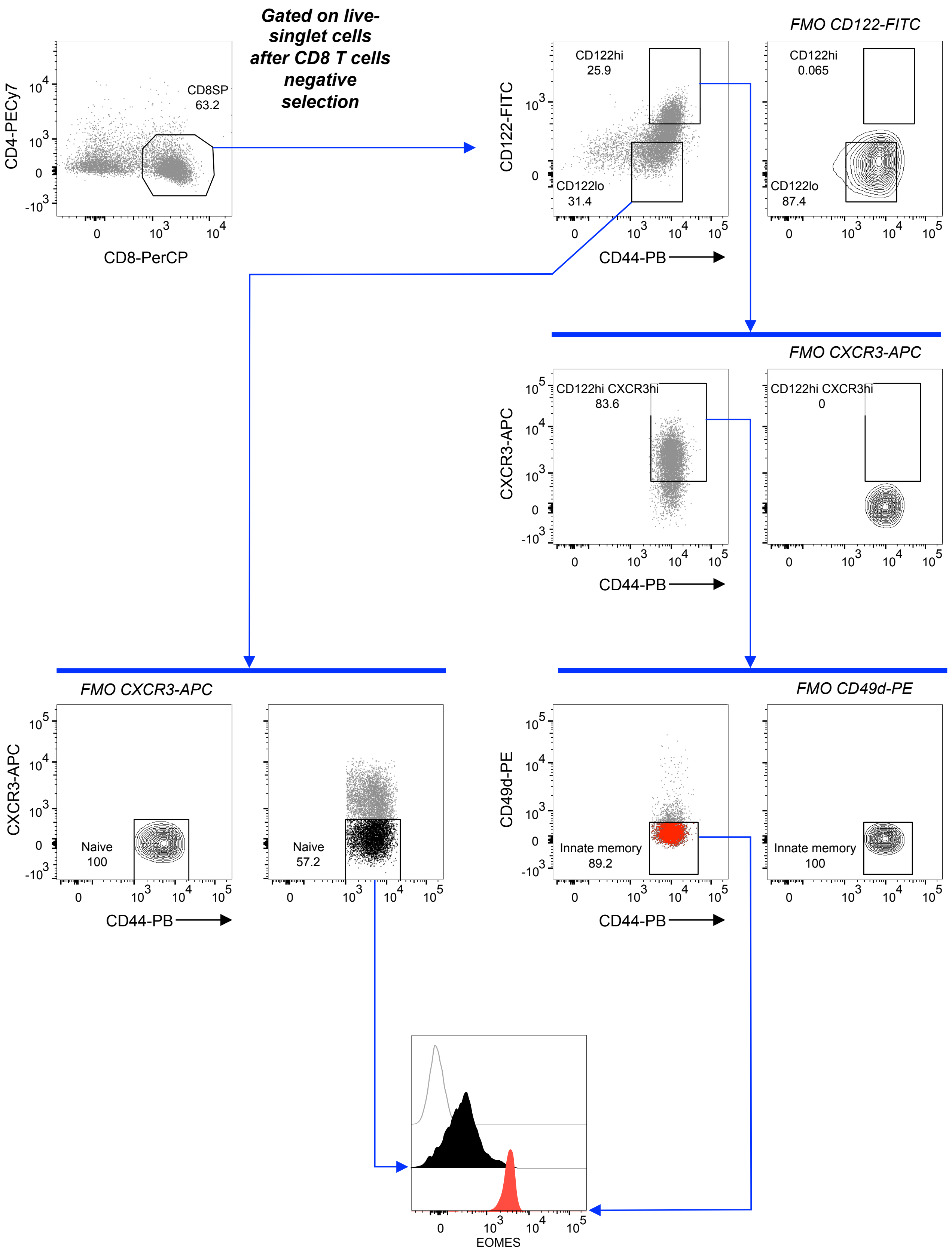

Supplementary Figure 2. Sorting strategy of naïve and T_{IM} CD8SP thymocytes from WT Balb/c mice. After negative selection of $CD8^+$ cells, untouched cells were stained to exclude dead cells and incubated with Fc receptor-blocking antibodies and a surface staining antibody mix to be sorted as T_{IM} ($CD8^+CD44^{hi}CXCR3^+CD122^+CD49d^{lo}$) or naïve ($CD8^+CD44^{lo}CXCR3^-CD122^-$) cells. The obtained T_{IM} cells are $EOMES^{hi}$, whereas naïve cells are $EOMES^{lo\text{-}int}$.

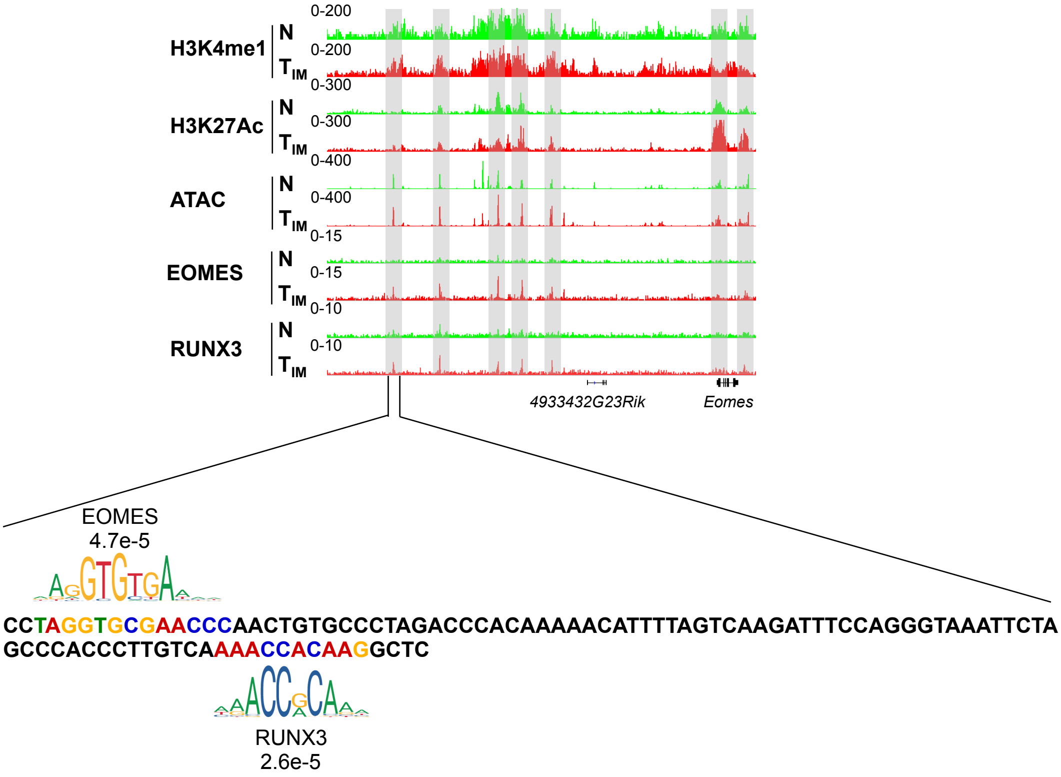

Supplementary Figure 3. Representative EOMES, RUNX3, H3K4me1, H3K27Ac ChIP-seq and ATAC-seq tracks at the *Eomes* locus in naïve and T_{IM} CD8SP thymocytes showing the co-localisation of EOMES and RUNX3 (highlighted in grey, top). Sequence of the indicated region and the location of EOMES and RUNX motifs with their position p-value are shown (down).

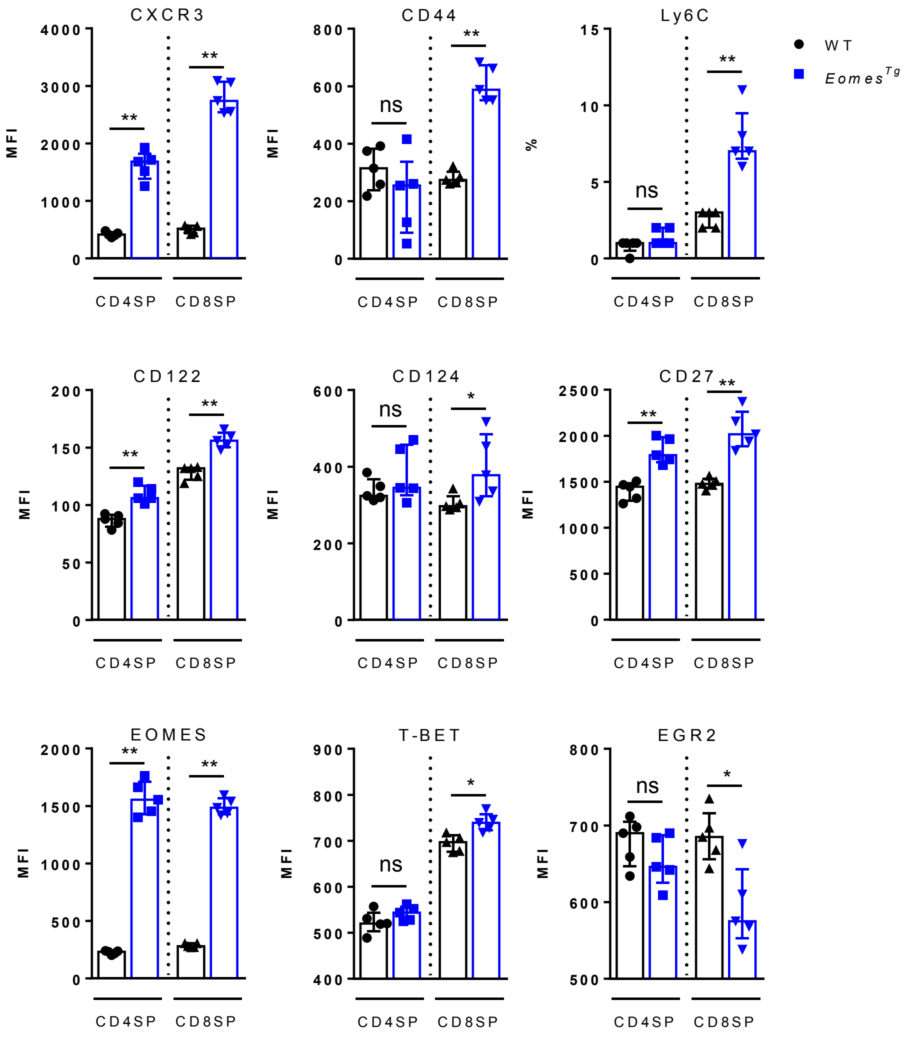

Supplementary Figure 4. Distinct effects of EOMES ectopic expression in CD4SP and CD8SP thymocytes. Thymocytes from WT or *Eomes*^{Tg} mice were analyzed by flow cytometry for the indicated protein. Although some markers follow similar patterns between CD3^{+}CD4SP and CD3^{+}CD8SP thymocytes upon EOMES overexpression (CXCR3, CD122, CD27), other markers are only impacted in CD3^{+}CD8SP (CD44, Ly6C, CD124, T-BET, EGR2), despite similar EOMES levels. Horizontal bars indicate median ± interquartile range (MFI or frequency). Each point represents a single mouse, n=5 mice per group). Statistics were calculated using Mann-Whitney test. ns not significant, * $P < 0.05$, ** $P < 0.01$.

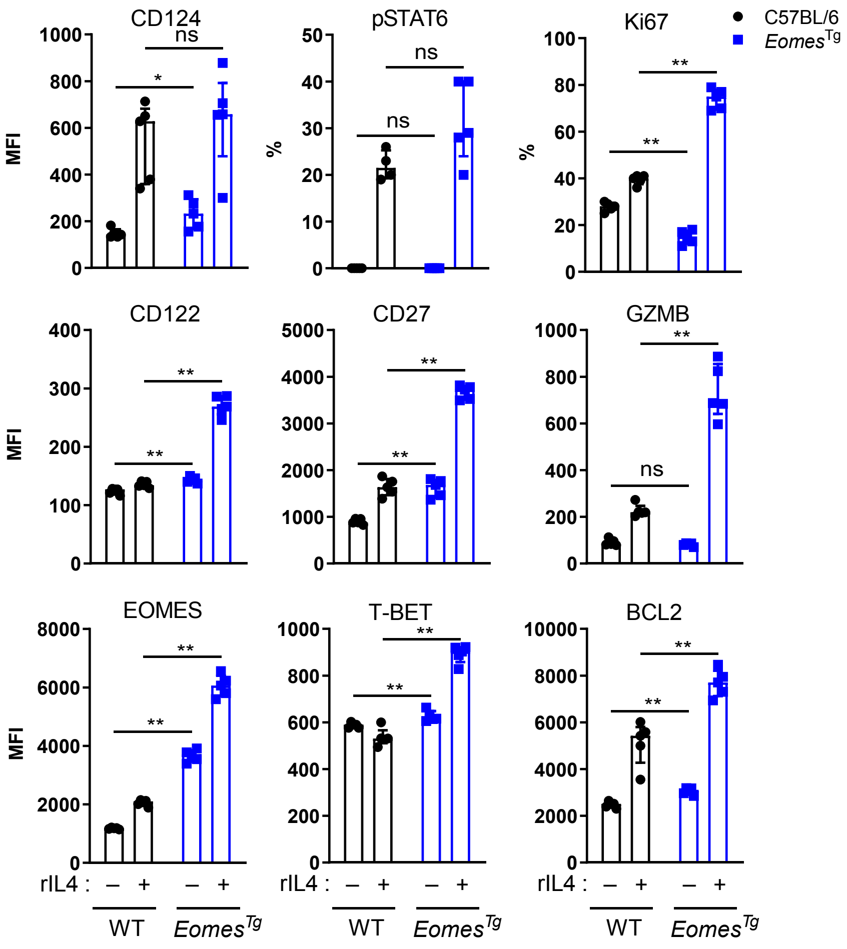

Supplementary Figure 5. CD8SP thymocytes from *Eomes*^{Tg} mice display heightened responsiveness upon *ex vivo* stimulation with rIL-4. Thymocytes from WT and *Eomes*^{Tg} mice were cultured in complete medium with or without rmIL-4 (20 ng ml^{-1}) for 72h, except for phospho-STAT6 staining (30 min culture with or without rmIL-4 at 30 ng ml^{-1}). Although CD124 basal levels are slightly higher in CD3^{+}CD8SP thymocytes from *Eomes*^{Tg} mice, early signalling (STAT6 phosphorylation) and latter CD124 upregulation (72h *ex vivo* IL-4 stimulation) are not different as compared to WT cells. Nonetheless, CD3^{+}CD8SP thymocytes from *Eomes*^{Tg} mice show stronger responses to IL-4 than WT mice, as they more readily proliferate (Ki67) and upregulate surface receptors (CD122, CD27) along with T-box transcription factors (EOMES, T-BET), effector (GZMB) and anti-apoptotic (BCL2) molecules. Horizontal bars indicate median ± interquartile range (MFI or frequency). Each point represents a single mouse, n=5 mice per group. Statistics were calculated using Mann-Whitney test. ns not significant, * $P < 0.05$, ** $P < 0.01$

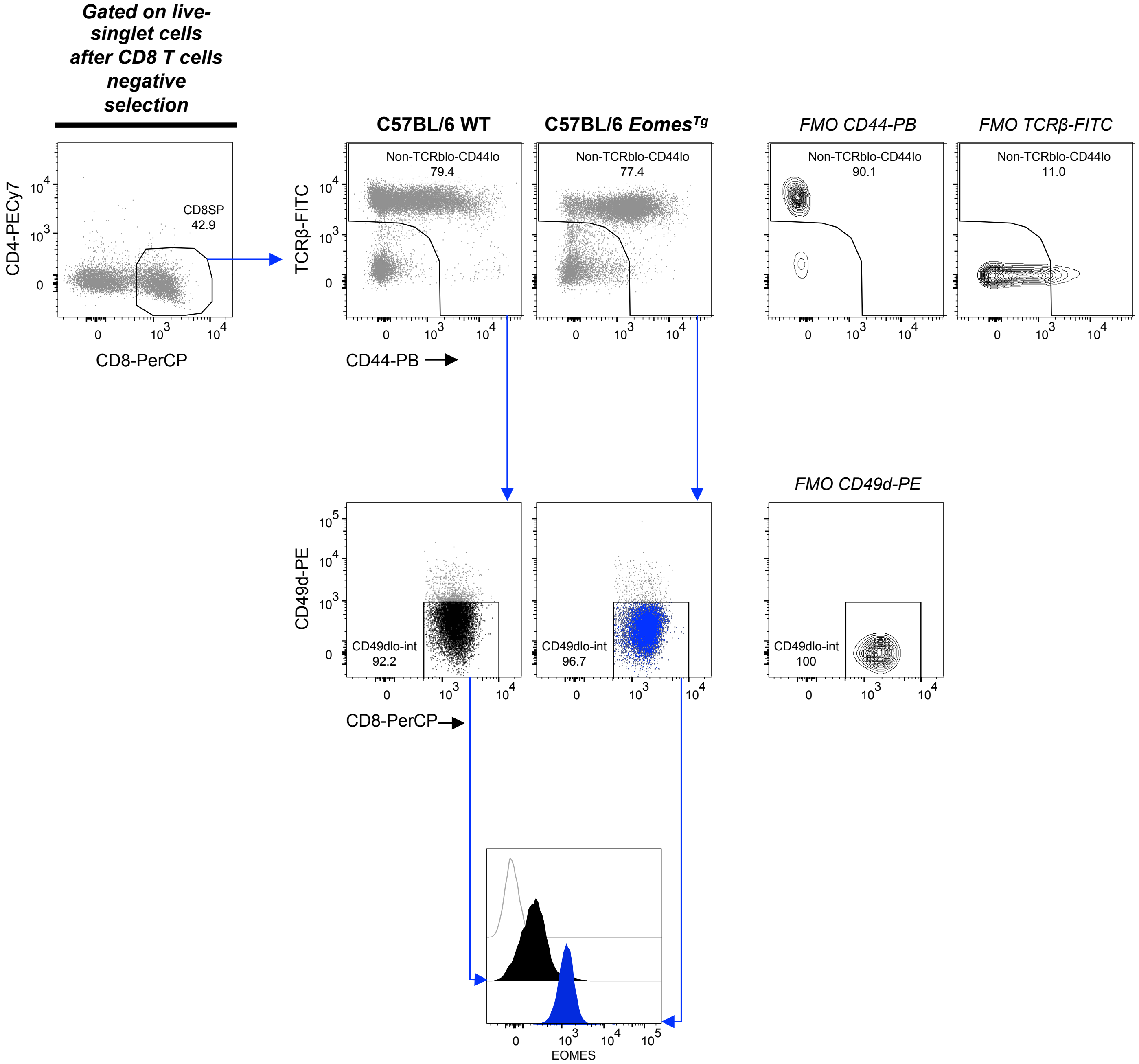

Supplementary Figure 6. Sorting strategy of CD8SP thymocytes from WT and *Eomes*^{Tg} C57Bl/6 mice. After CD8^{+} negative selection, untouched cells were stained to exclude dead cells and incubated with Fc receptor-blocking antibodies and a surface staining antibody mix to be sorted as WT or *Eomes*^{Tg} CD8SP thymocytes. In both cases, a TCRβ^{lo}CD44^{lo} population was removed from CD8^{+} cells, as were CD49d^{hi} cells.

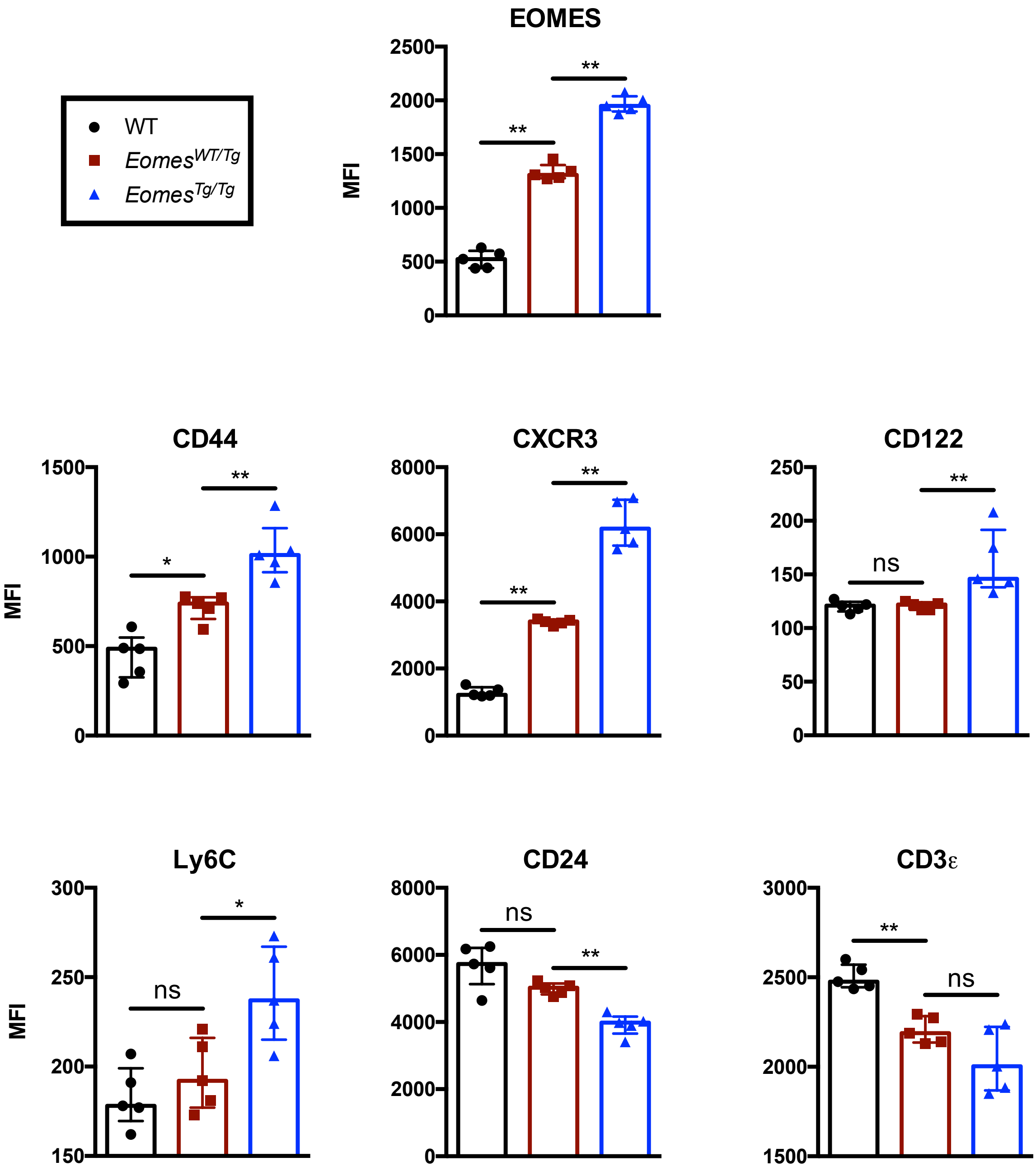

Supplementary Figure 7. EOMES ectopic expression in CD3⁺CD8SP thymocytes impacts the expression of several surface markers in a dose-dependent fashion. CD3⁺CD8SP thymocytes from WT, heterozygote (*Eomes*$^{WT/Tg}$) or homozygote transgenic (*Eomes*$^{Tg/Tg}$) mice expressing different levels of EOMES were analysed by flow cytometry for the indicated protein. Horizontal bars indicate median ± interquartile range (MFI). Each point represents a single mouse, n=5 mice per group. Statistics were calculated using Mann-Whitney test. ns not significant, * $P < 0.05$, ** $P < 0.01$

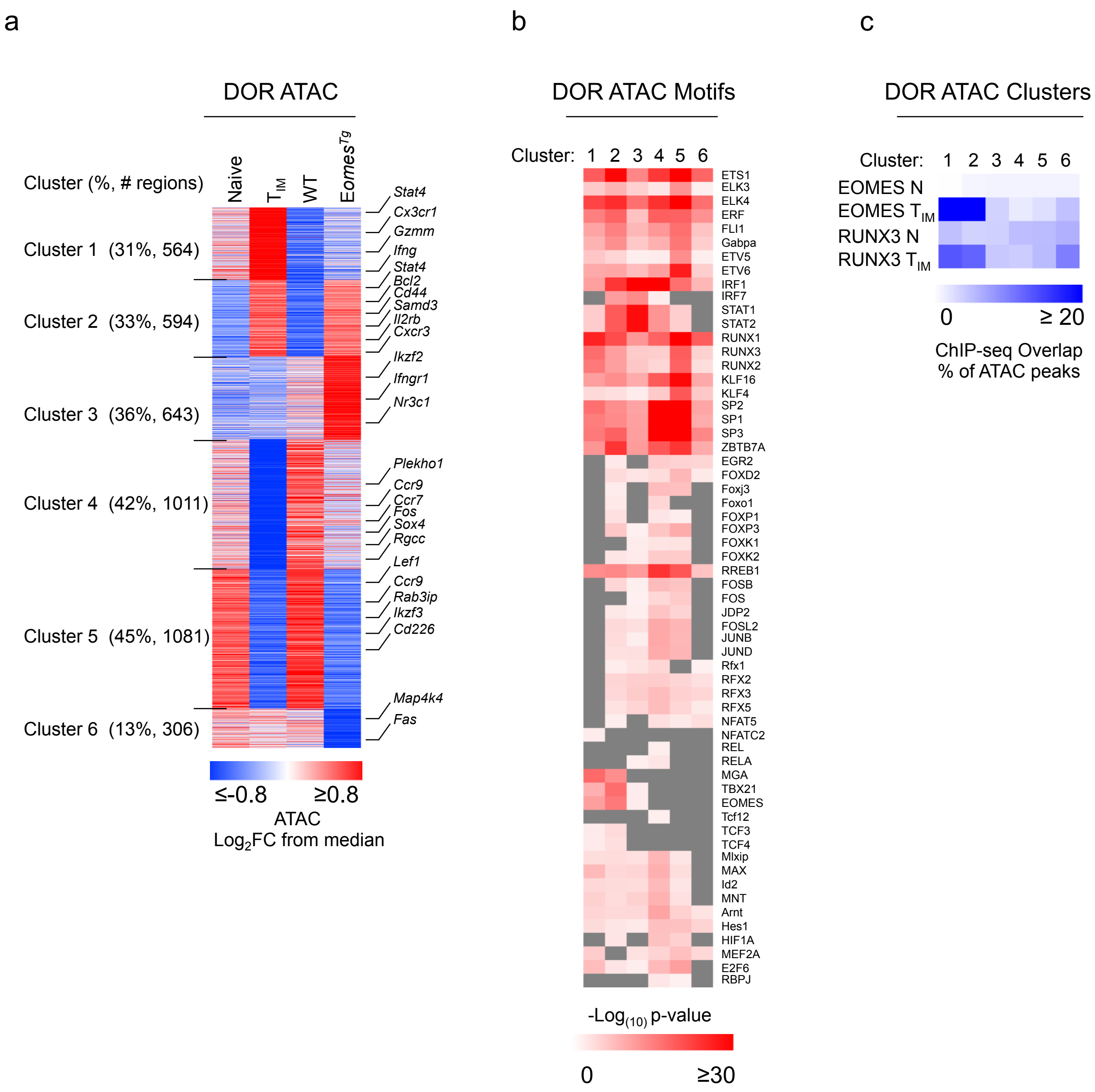

Supplementary Figure 8. T_{IM} and *Eomes*^{Tg} CD8SP thymocytes share differentially open regions in enhancers characterised by T-box motifs enrichment. a, Differentially open regions clustering within enhancers based on ATAC-seq signal in CD8SP thymocytes (Naïve, T_{IM}, WT and *Eomes*^{Tg}). Clusters in 1-3 and 4-6 are more or less open, respectively. Selected genes associated with each cluster are displayed in the right margin. **b**, Motif enrichment analysis of differentially active enhancers for clusters shown in **a** using AME and presented as -Log10 of p values. **c**, Percentage of regions within each enhancers' cluster that overlaps with ChIP-seq peaks for EOMES and RUNX3. ChIP-seq was performed on 3 independent immunoprecipitations (n=5 mice per sample).

a

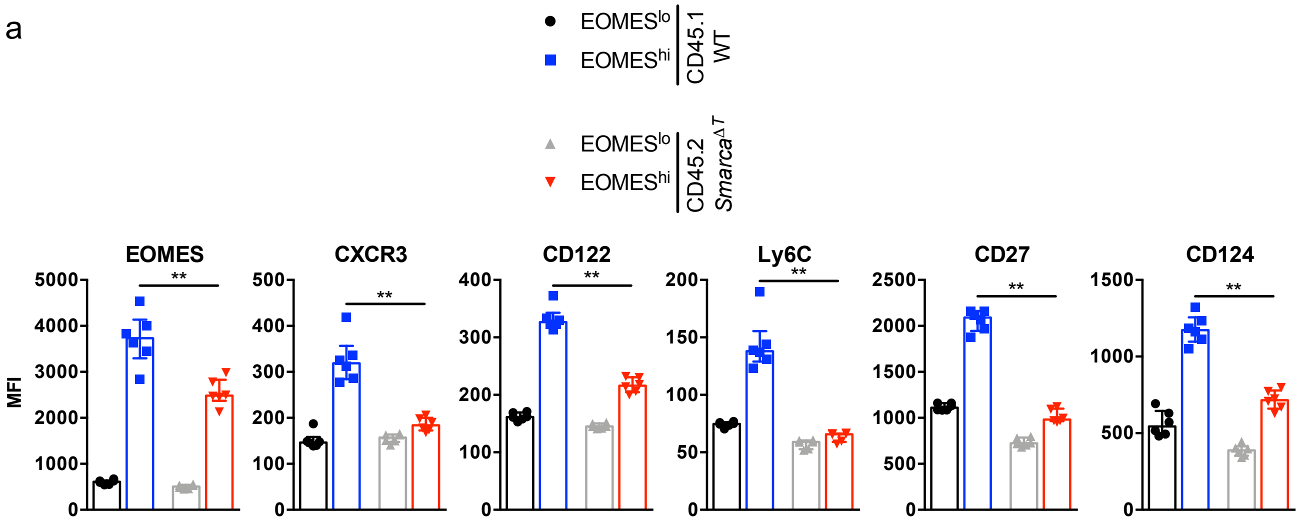

b

scEOMES (*x*)		scCXCR3 (*y*)	scCD122 (*y*)	scLy6C (*y*)	scCD27 (*y*)	scCD124 (*y*)
**Kendall's Tau**	***Smarca4****$^{fl/fl}$*	0.33	0.43	0.26	0.32	0.30
	Smarca4*$^{\Delta T}$*	0.06	0.19	0.08	0.13	0.24
**Fisher's Z transformation (p-value)**	***Are correlations different?***	*Yes* Z=28.94 (p<0.001)	*Yes* Z=27.23 (p<0.001)	*Yes* Z=19.33 (p<0.001)	*Yes* Z=9.67 (p<0.001)	*Yes* Z=8.07 (p<0.001)

Supplementary Figure 9. a, Data from the mixed bone marrow chimera experiment showing the expression (MFI) of cell markers in WT (CD45.1) or *Smarca4*$^{\Delta T}$ (CD45.2) EOMES^{lo} and EOMES^{hi} CD3^{+}CD8SP thymocytes after IL-4c injections. Horizontal bars indicate median ± interquartile range (MFI). Each point represents a single mouse, n=6 mixed bone marrow chimera (CD3ε$^{-/-}$ mice previously irradiated and injected with a mix of 50% bone marrow from CD45.1 WT mice and 50% bone marrow from CD45.2 *Smarca4*$^{\Delta T}$ mice) that all received 4 daily intraperitoneal IL-4c injections (see **Fig. 7d** experimental scheme). Statistics were calculated using Mann-Whitney test. ** P < 0.01. **b**, Kendall's Tau from correlations between single-cell (sc) fluorescences for EOMES and scCXCR3, scCD122, scLy6C, scCD27, and scCD124 are shown for the two representative IL-4c-injected mice (*Smarca4*$^{fl/fl}$ and *Smarca4*$^{\Delta T}$) displayed in **Fig. 7c** and compared using Fisher's Z transformation.

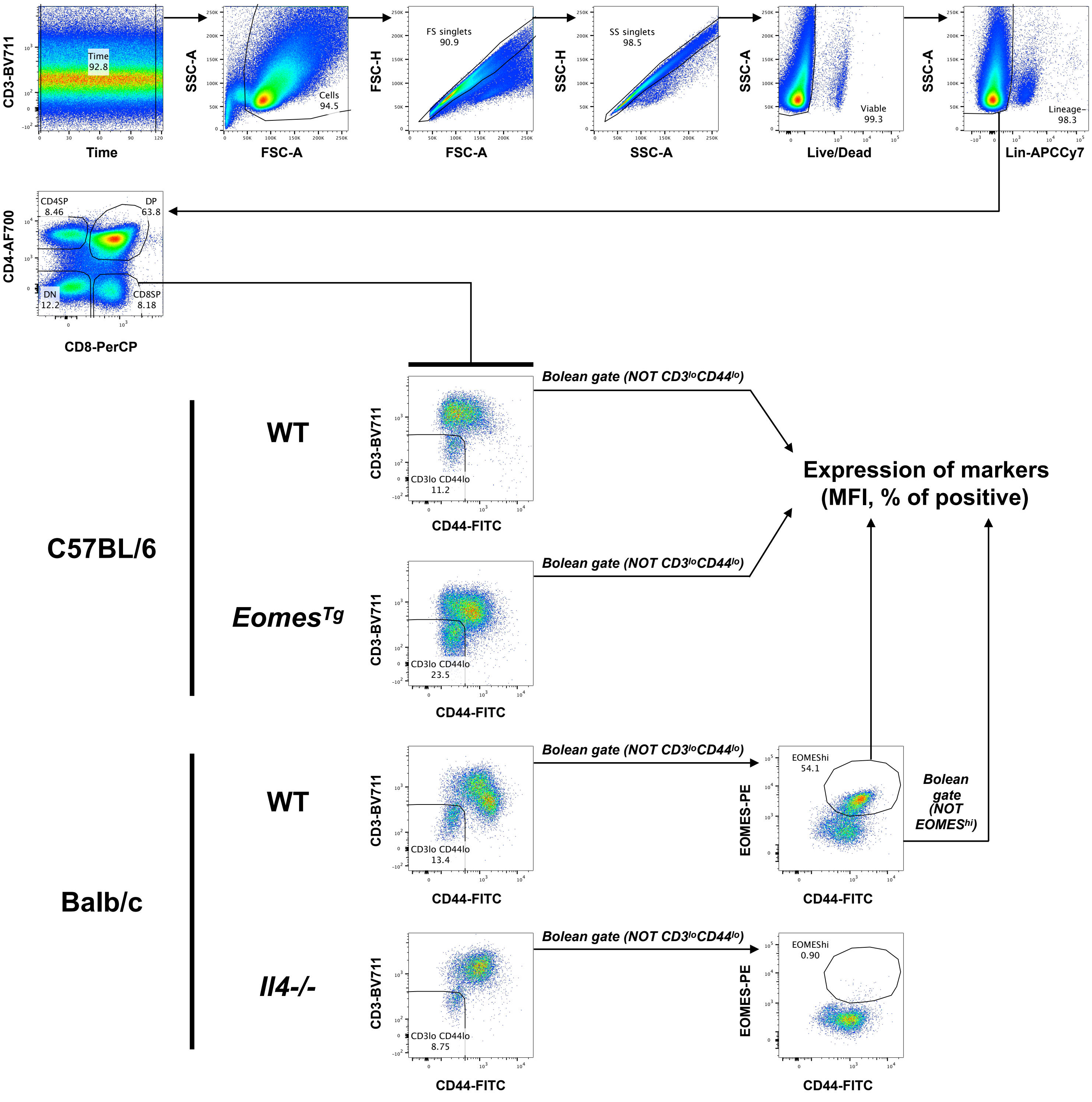

Supplementary Figure 10. Sequential gating strategy used to select, within a stable acquisition time frame, live-singlet non-CD3^{lo}CD44^{lo} CD8SP thymocytes (more generally referred to throughout the manuscript as CD3^{+}CD8SP thymocytes) and Balb/c EOMES^{hi} and EOMES^{lo} (Bolean gate NOT EOMES^{hi}) CD3^{+}CD8SP thymocytes. This strategy was universally applied to select CD3^{+}CD8SP thymocytes (**Fig. 1a-c**, **Fig. 5a-c**, **Fig. 7c-f**, **Supplementary Figures 1**, **4**, **5**, **7**, **9** and **11**), with the particularity that the selection of Lineage-negative cells (Lin: CD11b-CD11c-CD19-Gr1-NK1.1) was only applied in **Fig. 5a** to assess the frequencies of double negative-positive (DN-DP) and CD4-CD8 single positive (SP) thymocytes. CD8^{+} splenocytes (**Fig. 7g**,**h**) were selected as shown here for CD8SP thymocytes (without the Lineage gate) and naïve (N) CD8^{+} splenocytes were defined with a boolean gate NOT-TM AND NOT-VM (gating of VM and TM cells is shown in **Fig. 7g**).

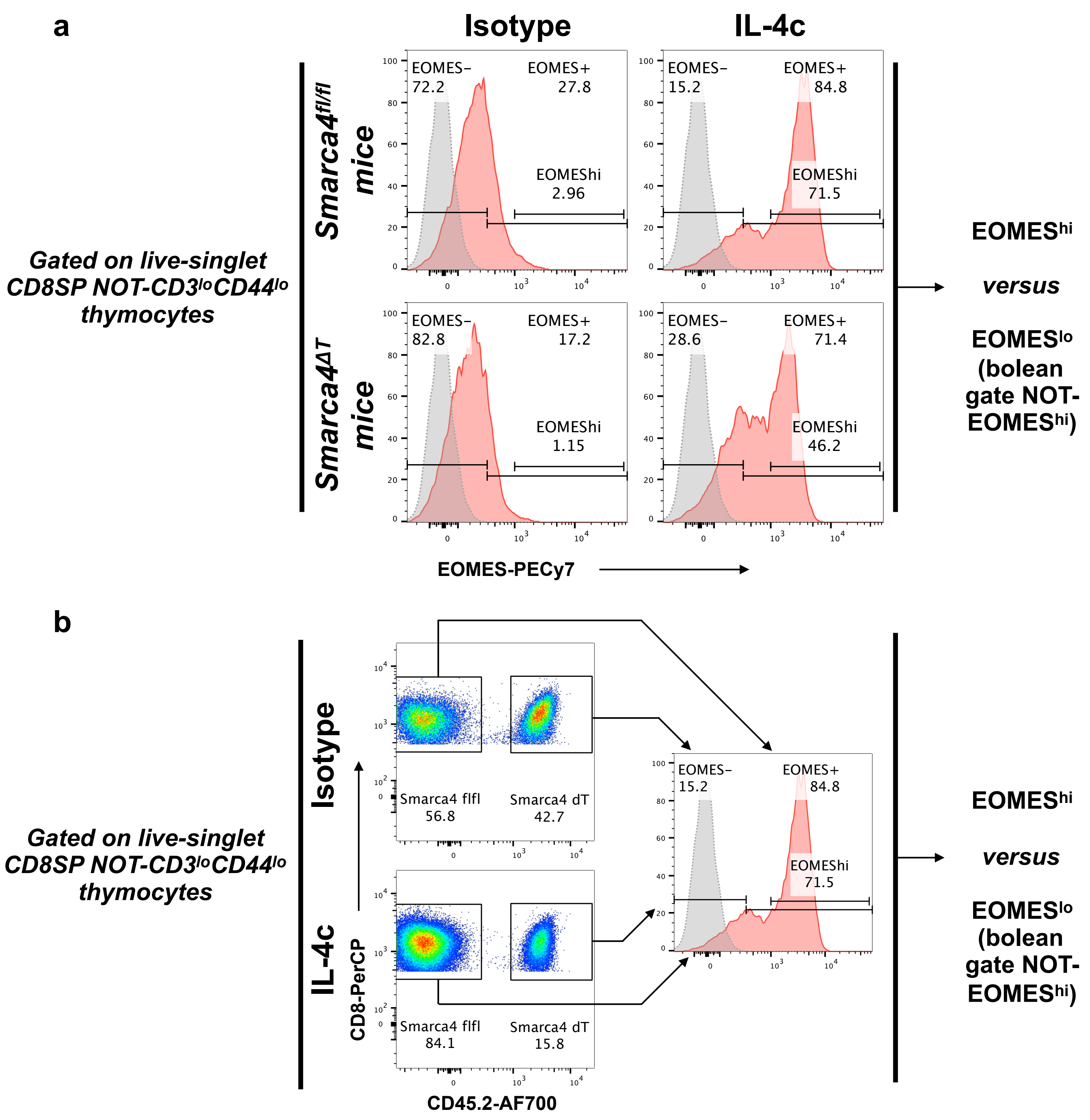

Supplementary Figure 11. a, Gating strategy used for the comparison of $EOMES^{lo}$ and $EOMES^{hi}$ $CD3^{+}$CD8SP thymocytes from *Smarca4$^{fl/fl}$* and *Smarca4$^{\Delta T}$* mice after isotype control or IL-4 complex (IL-4c) injection (this gating strategy was used for **Fig. 7c**,**e**,**f** and **Supplementary Figure 9b**). **b**, Gating strategy used for the comparison of $EOMES^{lo}$ and $EOMES^{hi}$ cells among *Smarca4$^{fl/fl}$* ($CD45.1^{+}CD45.2^{-}$) or *Smarca4$^{\Delta T}$* ($CD45.1^{-}CD45.2^{+}$) $CD3^{+}$CD8SP thymocytes from mixed bone marrow chimeras (see experimental scheme, **Fig. 7d**) after isotype control or IL-4 complex (IL-4c) injection (this gating strategy was used for **Fig. 7d** and **Supplementary Figure 9a**). Gray histograms represent fluorescence minus one controls, red histograms represent EOMES expression on gated cells. Percentages of cell populations are shown.
