# Supplementary material for: EOMES interacts with RUNX3 and BRG1 to promote innate memory cell formation through epigenetic reprogramming
Source: Nat Commun. 2019 Jul 24;10:3306. doi: 10.1038/s41467-019-11233-6 (PMC6656725; doi:10.1038/s41467-019-11233-6)
Supplement: Supplementary file 3 — Reporting Summary [file 41467_2019_11233_MOESM3_ESM.pdf]

nature research

Corresponding author(s): Stanislas GORIELY

Last updated by author(s): Jun 5, 2019

Reporting Summary

Nature Research wishes to improve the reproducibility of the work that we publish. This form provides structure for consistency and transparency in reporting. For further information on Nature Research policies, see Authors & Referees and the Editorial Policy Checklist.

Statistics

For all statistical analyses, confirm that the following items are present in the figure legend, table legend, main text, or Methods section.

n/a	Confirmed	
☐	☒	The exact sample size (*n*) for each experimental group/condition, given as a discrete number and unit of measurement
☐	☒	A statement on whether measurements were taken from distinct samples or whether the same sample was measured repeatedly
☐	☒	The statistical test(s) used AND whether they are one- or two-sided<br>*Only common tests should be described solely by name; describe more complex techniques in the Methods section.*
☒	☐	A description of all covariates tested
☒	☐	A description of any assumptions or corrections, such as tests of normality and adjustment for multiple comparisons
☐	☒	A full description of the statistical parameters including central tendency (e.g. means) or other basic estimates (e.g. regression coefficient) AND variation (e.g. standard deviation) or associated estimates of uncertainty (e.g. confidence intervals)
☐	☒	For null hypothesis testing, the test statistic (e.g. *F*, *t*, *r*) with confidence intervals, effect sizes, degrees of freedom and *P* value noted<br>*Give P values as exact values whenever suitable.*
☒	☐	For Bayesian analysis, information on the choice of priors and Markov chain Monte Carlo settings
☒	☐	For hierarchical and complex designs, identification of the appropriate level for tests and full reporting of outcomes
☐	☒	Estimates of effect sizes (e.g. Cohen's *d*, Pearson's *r*), indicating how they were calculated

Our web collection on statistics for biologists contains articles on many of the points above.

Software and code

Policy information about availability of computer code

Data collection	Flow cytometry data were acquired on a BD LSRFortessa™ cell analyzer after successfully running Cytometer Setup and Tracking beads (BD) and using the application settings featured on FACSDiva™ software (v 6.2).<br>Cells were sorted on a BD FACSAria™ III.<br>Immunoblot images were obtained on a Biorad Universal Hood II Gel Doc System.
Data analysis	Flow cytometry data were analyzed using the FlowJo v10 software (Tree Star) and the spanning-tree analysis tool SPADE V3.0 (http://pengqiu.gatech.edu/software/SPADE/).<br>Immunoblot images were analyzed using the Quantity One analysis software.<br>Prism 6.0 was used for statistical analysis. Mann-Whitney test was used to compare two data sets. For correlation between single-cell fluorescence of EOMES and innate memory markers, R and Rkward (https://rkward.kde.org) were used to compute Kendall's tau (single nonlinear correlation) and Fisher Z transformation (comparison of two independent correlations). For all analyses, no data points were excluded.<br>Seqmonk was used to analyse ChIP-seq and ATAC-seq data.<br>usegalaxy.org/deeptool suit was used to generate FrIP normalized bigwig files.<br>Genomic Regions Enrichment of Annotations Tool (GREAT) was used to analyse the functional significance of ATAC-seq differential regions.<br>Ciiider and meme-suit were used for motif analysis.<br>Bubblegum and GSEA (Broad institute) were used for gene set enrichment analysis.<br>cistrome.org/ap/ and BETA were used for binding and expression target analysis.<br>MeV (Multiple Experiment Viewer) was used to generate the heatmaps.<br>IGV browser (Broad institute) was used to visualise the normalised bigwig files.

For manuscripts utilizing custom algorithms or software that are central to the research but not yet described in published literature, software must be made available to editors/reviewers. We strongly encourage code deposition in a community repository (e.g. GitHub). See the Nature Research guidelines for submitting code & software for further information.

Data

Policy information about availability of data

All manuscripts must include a data availability statement. This statement should provide the following information, where applicable:

- Accession codes, unique identifiers, or web links for publicly available datasets
- A list of figures that have associated raw data
- A description of any restrictions on data availability

RNA-Seq, ChIP-Seq and ATAC-Seq data that support the findings reported in this study have been deposited in the GEO Repository with the accession code GSE124914

Field-specific reporting

Please select the one below that is the best fit for your research. If you are not sure, read the appropriate sections before making your selection.

☒ Life sciences ☐ Behavioural & social sciences ☐ Ecological, evolutionary & environmental sciences

For a reference copy of the document with all sections, see nature.com/documents/nr-reporting-summary-flat.pdf

Life sciences study design

All studies must disclose on these points even when the disclosure is negative.

Sample size

Sample size was determined according to standard practice in the field. For FACS data, 4 to 7 mice per group were included. ChIP-seq data were obtained from at least three independent experiments and then pooled together. ATAC-seq data were obtained in duplicates (for each group). RNA-seq data were obtained in triplicates (from 8-10 pooled thymus). No statistical methods were used to predetermine sample sizes.

Data exclusions

No data points were excluded from analyses. All individual data points are shown in bar plot graphs.

Replication

Experimental data were consistently reproduced. All data presented are from representative independent experiments that were not pooled with other similar experiments.

Figure 1a: Data are representative of four experiments (n = 5 mice). Histograms show naive and innate memory levels of expression for one representative WT Balb/c mouse.
Figure 1b-d: Data are representative of 2 experiments (n = 4 mice per group). Four WT Balb/c mice were used for tree construction, one representative mouse is shown per group (WT, Il4-/-, Stat6-/-, Irf9-/- Balb/c mice). Figure 1b and Supplementary Figure 1: “Expr” was selected as color definition, and “JET” selected as color scheme. Figure 1c-d: “CellFreq” was selected as color definition, and “Gray scale” selected as color scheme (see http://pengqiu.gatech.edu/software/SPADE/). SPADE trees presented on Figure 1b-d and Supplementary Figure 1 were constructed using K-means as clustering parameter and the following markers: CD3, CD24, CXCR3, CD122, CD62L, Ly6C, and EOMES.
Figure 1e-f, Supplementary Data 1 and 4: RNA-Seq data are in triplicates for naive and innate memory cells (one experiment).
Figure 2a-d, Figure 4d,g, Supplementary Fig. 3, Supplementary Data 2: H3K4me3- H3K4me1, and H3K27Ac-ChIP-seq data were obtained from at least three independent IP and then pooled together.
Figure 2e, Figure 4d,g, Supplementary Figure 3 and 8, Supplementary Data 3: ATAC-seq data were obtained from duplicates.
Figure 3a,b, Supplementary Data 4: ChIP-seq data were obtained from at least three independent IP and then pooled together.
Figure 4a: RIME was performed on 50 million pooled WT Balb/c mice splenocytes after polyclonal expansion. One experiment.
Figure 4b-d, Figure 6f, Supplementary Fig. 3: EOMES- and RUNX3-ChIP-seq data were obtained from at least three independent IP and then pooled together.
Figure 5a: Data are representative of more than three experiments (n = 7 mice per group).
Figure 5b: Data representative of two experiments or more, n = 5 mice per group for WT or EomesTg C57Bl/6 mice, n = 4 or 5 per group for Balb/c mice).
Supplementary Fig. 7: Data representative of two experiments, n = 5 mice per group for WT or EomesWTTg or EomesTgTg C57Bl/6 mice.
Figure 5c, Supplementary Figures 4 and 5: Data are representative of three experiments for WT and EomesTg mice, and of two experiments for WT Balb/c mice.
Figure 5d-f, Supplementary Data 1: RNA-Seq data are in triplicates for WT and EomesTg C57Bl/6 mice (one experiment).
Figure 6a, Supplementary Figure 8, Supplementary Data 3: ATAC-seq data were obtained in duplicates.
Figure 6c, e-f, Supplementary Data 2: H3K4me3- H3K4me1, and H3K27Ac-ChIP-seq data were obtained from at least three reproducible experiments and then pooled together.
Figure 7a: One experiment per co-immunoprecipitation.
Figure 7b: One experiment. Thymi from 7 WT versus 7 EomesTg mice were pooled. One independent IP per antibody.
Figure 7c: Proportions of cells based on CD44+/- and EOMESlo/hi expressions are shown in FACS plots for one mouse representative of each condition (numbers in plots indicate the percentage of cells that are outlined), and the proportions of EOMEShi cells are shown in bar plots (one experiment, n = 5 mice per group).
Figure 7d, Supplementary Fig. 9a: Ten previously irradiated CD3e-/- mice received a 50:50 bone marrow mixture (WT CD45.1 : Smarca4ΔT CD45.2). Four of these mice received an isotype control, and 6 mice received IL-4c. One experiment.
Figure 7e: One experiment, n = 5 mice per group.
Figure 7f, Supplementary Fig. 9b: Representative correlation plots of EOMES and CXCR3 single-cell (sc) fluorescences in CD3+CD8SP thymocytes from Smarca4fl/fl (left, same mouse as the one displayed in the upper right plot from Fig 7c) and Smarca4ΔT (right, same mouse as the one displayed in the lower right plot from Fig 7c) after IL-4c injections. Kendall’s Tau from correlations between scEOMES and scCXCR3, scCD122, scLy6C, scCD27, and scCD124 are shown for these two mice and compared using Fisher’s Z transformation.
Figure7g-h: One experiment, n = 5 mice per group.

Randomization	Mice were grouped according to genotype. No randomization was used to attribute treatments in in vivo studies.
Blinding	No blinding was applied in this study.

Reporting for specific materials, systems and methods

We require information from authors about some types of materials, experimental systems and methods used in many studies. Here, indicate whether each material, system or method listed is relevant to your study. If you are not sure if a list item applies to your research, read the appropriate section before selecting a response.

Materials & experimental systems

n/a	Involved in the study
☐	☒ Antibodies
☐	☒ Eukaryotic cell lines
☒	☐ Palaeontology
☐	☒ Animals and other organisms
☒	☐ Human research participants
☒	☐ Clinical data

Methods

n/a	Involved in the study
☐	☒ ChIP-seq
☐	☒ Flow cytometry
☒	☐ MRI-based neuroimaging

Antibodies

Antibodies used

All FACS antibodies were anti-mouse or anti-human showing a validated anti-mouse reactivity (fluorochrome[s]; clone; dilution[s]; catalog number[s]):

- From BD: BCL2 (PE; 3F11; 1:10; 556537), CD3 (BV711; 145-2C11; 1:100; 563123), CD4 (AF700, APC-Cy7, PE-Cy7; RM4-5; 1:100; 561025, 565650, 561099), CD5 (BV421; 53-7.3; 1:100; 562739), CD8 (PerCP, PB; 53-6.7; 1:50; 561092, 558106), CD11c (APC-Cy7; HL3; 1:100; 561241), CD16/CD32 (none; 2.4G2; 1:100; 553141), CD19 (APC-Cy7; 1D3; 1:100; 561737), CD24 (BV605, PE; M1/69; 1:100; 563060, 553262), CD25 (APC; PC61; 1:50; 561048), CD27 (BV786; LG.3A10; 1:100; 740890), CD44 (APC, FITC, V450; IM7; 1:50; 561862, 561859, 560451), CD49d (BV650, PE; R1-2; 1:200, 1:50; 740458, 553157), CD62L (BV786; MEL-14; 1:100; 564109), CD69 (BV786; H1.2F3; 1:100; 564683), CD103 (BV421; M290; 1:100; 562771), CD122 (FITC; TM-β1; 1:50; 553361), CD124 (BV421, PE; mIL4R-M1; 1:100; 564086, 552509), CD127 (BV786, PE-Cy7; SB/199; 1:100, 1:50; 563748, 560733), CXCR3 (APC; CXCR3-173; 1:50; 562266), GZMB (AF647; GB11; 1:100; 560212), IFNγ (BV786; XMG1.2; 1:200; 563773), Ki67 (AF700; B56; 1:50; 561277), Ly6C (BV605, FITC; AL-21; 1:50; 563011, 553104), Ly6C and Ly6G (APC-Cy7; RB6-8C5; 1:100; 557661), NK1.1 (APC-Cy7; PK136; 1:100; 560618), STAT6 (pY641) (AF488; J71-773.58.11; 2:3; 558243), TCRβ (BV605, FITC; H57-597; 1:100; 562840, 553170)

- From eBioscience: EGR2 (PE; erongr2; 1:50; 12-6691-82), EOMES (eFluor 660, PE, PE-Cy7; Dan11mag; 1:100; 50-4875-82, 12-4875-82, 25-4875-82), T-BET (eFluor 660, PE, PE-Cy7; eBio4B10; 1:100; 50-5825-82, 12-5825-82, 25-5825-82)

- From Biolegend: CD69 (BV605; H1.2F3; 1:50; 104530)

Antibodies used for ChIP, EOMES Rapid Immunoprecipitation Mass spectrometry of Endogenous proteins, and EOMES-BRG1 co-immunoprecipitations and immunoblots were as follows:
Anti-H3K4me3 (1µg, Merck, catalog number 17-614), anti-H3K27ac (1µg, Abcam, catalog number ab4729), anti-H3K4me1 (1µg, Abcam, catalog number ab8895), normal rabbit IgG control (1µg, Merck, catalog number 12-370), anti-EOMES (4µg, Abcam, catalog number ab23345), anti-BRG1 (2-4µg, Abcam, catalog number ab110641), anti-RUNX3 (dilution 1:250, Gently provided by Prof. Yoram Groner).

Validation

Antibodies were validated by the manufacturer. For some FACS antibodies, optimal dilution was determined by titration and based on observed staining indexes.

Eukaryotic cell lines

Policy information about cell lines

Cell line source(s)	293 cells (ATCC Cat#CRL-1573).
Authentication	This cell line was not authenticated.
Mycoplasma contamination	This cell line was tested negative for Mycoplasma contamination.
Commonly misidentified lines (See ICLAC register)	None were used in this study.

Animals and other organisms

Policy information about studies involving animals; ARRIVE guidelines recommended for reporting animal research

Laboratory animals	All experiments were performed on age-matched (from 8 to 12 weeks of age) female mice. Wild-type Balb/c and C57Bl/6 mice were purchased from Envigo. Il4-/-and Stat6-/- mice under Balb/c background were purchased from the Jackson Lab. Irf9-/- mice under Balb/c background were purchased from Riken BioResearch Center. CD3ε-/- and CD45.1 mice under C57Bl/6 background were obtained from the Jackson Lab. Smarca4fl/fl mice under C57BL/6 background were kindly provided by Professor Pierre Chambon, GIE-CERBM (IGBMC) and were crossed to CD4Cre+ mice obtained from the Jackson Lab (Tg(Cd4-cre)1Cwi/BfluJ, Stock 017336) to generate Smarca4ΔT mice. In order to generate Eomes transgenic mice, a 2.1KB full-length cDNA encoding mouse EOMES was inserted into the VA-CD2 cassette containing the upstream gene regulatory region and locus control region of the hCD2 gene. This construct was linearized and injected into BDF1 fertilized eggs to generate EomesTg mice. Mice were backcrossed for 6 generations onto C57Bl/6 strain and then intercrossed in order to obtain homozygote transgenic mice (i.e. the de facto EomesTg mice). All animal work was carried out in compliance with and after approval by the institutional Animal Care and local committee for animal welfare.
Wild animals	None were used in this study.
Field-collected samples	N/A
Ethics oversight	All animal work was carried out in compliance with and after approval by the institutional Animal Care and local committee for animal welfare.

Note that full information on the approval of the study protocol must also be provided in the manuscript.

ChIP-seq

Data deposition

☒ Confirm that both raw and final processed data have been deposited in a public database such as GEO.

☒ Confirm that you have deposited or provided access to graph files (e.g. BED files) for the called peaks.

Data access links
May remain private before publication.

http://www.ncbi.nlm.nih.gov/projects/geo/query/acc.cgi?acc=GSE124914

Files in database submission

GSE124913 EOMES-dependent epigenetic reprogramming during CD8 T cell development leads to acquisition of memory traits (RNA-Seq) Dec 31, 2019 approved CSV XLSX XLSX

GSM3559335 RNA-Seq_CD8_TIM_S1 Dec 31, 2019 approved None
GSM3559336 RNA-Seq_CD8_TIM_S2 Dec 31, 2019 approved None
GSM3559337 RNA-Seq_CD8_TIM_S3 Dec 31, 2019 approved None
GSM3559338 RNA-Seq_CD8_Naive_S1 Dec 31, 2019 approved None
GSM3559339 RNA-Seq_CD8_Naive_S2 Dec 31, 2019 approved None
GSM3559340 RNA-Seq_CD8_Naive_S3 Dec 31, 2019 approved None
GSM3559341 RNA-Seq_CD8SP-WT_S1 Dec 31, 2019 approved None
GSM3559342 RNA-Seq_CD8SP-WT_S2 Dec 31, 2019 approved None
GSM3559343 RNA-Seq_CD8SP-WT_S3 Dec 31, 2019 approved None
GSM3559344 RNA-Seq_CD8SP-EomesTG_S1 Dec 31, 2019 approved None
GSM3559345 RNA-Seq_CD8SP-EomesTG_S2 Dec 31, 2019 approved None
GSM3559346 RNA-Seq_CD8SP-EomesTG_S3 Dec 31, 2019 approved None
----
GSE124912 EOMES-dependent epigenetic reprogramming during CD8 T cell development leads to acquisition of memory traits (ChIP-Seq) Dec 31, 2019 approved None
GSM3559315 H3K4me3_TIM Dec 31, 2019 approved BIGWIG BROADPEAK
GSM3559316 H3K4me3_Naive Dec 31, 2019 approved BIGWIG BROADPEAK
GSM3559317 H3K4me3_CD8SP-WT Dec 31, 2019 approved BROADPEAK
GSM3559318 H3K4me3_CD8SP-EomesTG Dec 31, 2019 approved BROADPEAK
GSM3559319 H3K4me1_TIM Dec 31, 2019 approved BIGWIG BROADPEAK
GSM3559320 H3K4me1_Naive Dec 31, 2019 approved BIGWIG BROADPEAK
GSM3559321 H3K27ac_TIM Dec 31, 2019 approved BIGWIG BROADPEAK
GSM3559322 H3K27ac_Naive Dec 31, 2019 approved BIGWIG BROADPEAK
GSM3559323 H3K4me1_CD8SP-WT Dec 31, 2019 approved BIGWIG BROADPEAK
GSM3559324 H3K4me1_CD8SP-EomesTG Dec 31, 2019 approved BIGWIG BROADPEAK
GSM3559325 H3K27ac_CD8SP-WT Dec 31, 2019 approved BIGWIG BROADPEAK
GSM3559326 H3K27ac_CD8SP-EomesTG Dec 31, 2019 approved BIGWIG BROADPEAK
GSM3559327 Eomes_TIM Dec 31, 2019 approved BIGWIG NARROWPEAK
GSM3559328 Eomes_Naive Dec 31, 2019 approved BIGWIG NARROWPEAK
GSM3559329 Runx3_TIM Dec 31, 2019 approved BIGWIG NARROWPEAK

GSM3559330 Runx3_Naive Dec 31, 2019 approved BIGWIG
NARROWPEAK
GSM3559331 Input_TIM Dec 31, 2019 approved BIGWIG
GSM3559332 Input_Naive Dec 31, 2019 approved BIGWIG
GSM3559333 Input_CD8SP-WT Dec 31, 2019 approved None
GSM3559334 Input_CD8SP-EomesTG Dec 31, 2019 approved None

GSE124911 EOMES-dependent epigenetic reprogramming during CD8 T cell development leads to acquisition of memory traits (ATAC-Seq) Dec 31, 2019 approved None
GSM3559311 ATAC_TIM Dec 31, 2019 approved BIGWIG
NARROWPEAK NARROWPEAK
GSM3559312 ATAC_Naive Dec 31, 2019 approved BIGWIG
NARROWPEAK NARROWPEAK
GSM3559313 ATAC_CD8SP-WT Dec 31, 2019 approved BIGWIG
NARROWPEAK NARROWPEAK
GSM3559314 ATAC_CD8SP-EomesTG Dec 31, 2019 approved BIGWIG
NARROWPEAK NARROWPEAK

Genome browser session
(e.g. UCSC)

not accessible yet

Methodology

Replicates

ChIP-seq data were obtained from at least three independent IPs and then pooled together. ATAC-seq data were obtained in duplicates (for each group). RNA-seq data were obtained in triplicates (from 8-10 pooled thymus).

Sequencing depth

All NGS experiments have a sequencing depth between 25-50 millions mapped reads, except TIM ATAC-seq samples which have around 8 millions reads.

Antibodies

Anti-H3K4me3 (Merck, 17-614), anti-H3K27ac (Abcam, ab4729), anti-H3K4me1 (Abcam, ab8895) or normal rabbit IgG control (Merck, 12-370), anti-EOMES (Abcam, ab23345), anti-BRG1 (Abcam, ab110641), anti-RUNX3 (Gently provided by Prof. Yoram GRONER).

Peak calling parameters

Peaks were then called with callpeak tool from MACS2 with a q-value of 0.01 for H3K4me3, H3K4me1 and H3K27ac ; with a p-value of 0.01 for RUNX3, and with a p-value of 0.001 for EOMES

Data quality

Reads were mapped to mouse genome mm10 with Bowtie2 using default parameters. Reads that mapped several regions, or with insufficient mapping quality, were removed with samtools view. We also removed reads located within the blacklist of the ENCODE project. Duplicate reads were removed with MarkDuplicates tools (Picard suite).

Software

Bowtie2 was used for mapping.
Picard suite was used to remove duplicates.
MACS2 was used for peak calling.

Flow Cytometry

Plots

Confirm that:

☒ The axis labels state the marker and fluorochrome used (e.g. CD4-FITC).

☒ The axis scales are clearly visible. Include numbers along axes only for bottom left plot of group (a 'group' is an analysis of identical markers).

☒ All plots are contour plots with outliers or pseudocolor plots.

☒ A numerical value for number of cells or percentage (with statistics) is provided.

Methodology

Sample preparation

Thymi and spleens were dissected and further processed under sterile conditions. Single-cell suspensions were obtained in RPMI 1640 with 10% (vol/vol) fetal calf serum (FCS), 2 mM l-glutamine, 1 mM sodium pyruvate, 0.1 mM non-essential amino acids, 40 mM β-mercaptoethanol, 100 U ml-1 of penicillin and 100 U ml-1 of streptomycin. Single-cell suspensions were washed in PBS, then stained to exclude dead cells (LIVE/DEAD™ Fixable Aqua Dead Cell Stain Kit, for 405 nm excitation, Life Technologies). Cells were washed with PBS with 2% bovine serum albumine and incubated with Rat anti-mouse CD16/CD32 (BD) and a surface antibody mix prepared in Brilliant Stain Buffer (BD) for 20 minutes at 4°C in the dark (for sorting, washes and staining were performed in PBS with 10% FCS and 2 mM Ethylenediaminetetraacetic acid (EDTA, Sigma)). Except for sorting, cells were fixed and permeabilized for 30 minutes at 4°C in the dark (eBioscience™ Foxp3/Transcription Factor Staining Buffer Set, Life Technologies) before intranuclear/intracytoplasmic staining (30 minutes at 4°C in the dark). For phospho-STAT6 staining, we adapted the harsh alcohol method (BD Phosflow protocol III).

Instrument	Cells were sorted on a BD FACSAria™ III using fluorescence minus one (FMO) controls (see Supplementary Fig. 2 and 5 for gating strategies). Flow cytometry data were acquired on a BD LSRFortessa™ cell analyzer after successfully running Cytometer Setup and Tracking beads (BD) and using the application settings featured on FACSDiva™ software (v 6.2).
Software	The FACSDiva™ software (v 6.2) was used to collect the data. Data were analyzed using FlowJo v10 software (Tree Star) and the spanning-tree analysis tool SPADE V3.0 (http://pengqiu.gatech.edu/software/SPADE/).For correlation between single-cell fluorescence of EOMES and innate memory markers, R and Rkward (https://rkward.kde.org) were used to compute Kendall's tau (single nonlinear correlation) and Fisher Z transformation (comparison of two independent correlations).
Cell population abundance	A minimum of 10.000 target cells (CD3+CD8SP thymocytes or splenocytes) were recorded per replicate.
Gating strategy	Cells were gated on a Time/SSC-A plot to select only the stable part of events acquisition. A large gate was set on a FSC-A/SSC-A plot, and singlets were selected on an FSC-A/FSC-H plot , then on an SSC-A/SSC-H plot. Dead cells were eliminated on a Live Dead/SSC-A plot. Detailed gating strategies (FACS) are shown in Supplementary Figures 10 and 11, and detailed gating strategies for cell sorting are shown in Supplementary Fig. 2 (naive and innate memory cells from Balb/c mice) and 5 (TCRβ+CD8SP thymocytes from C57Bl/6 WT and EomesTg mice). For FACS data, CD3+CD8SP thymocytes were gated using a CD4/CD8 plot and a CD44/CD3 plot. When studying DN and DP thymocytes, a dump gate was added to this gating strategy: after eliminating dead cells, lineage-negative cells were selected based on a Lineage (CD11b, CD11c, Gr1, NK1.1, CD19) / SSC-A plot.

☒ Tick this box to confirm that a figure exemplifying the gating strategy is provided in the Supplementary Information.
