# Supplementary material for: EOMES interacts with RUNX3 and BRG1 to promote innate memory cell formation through epigenetic reprogramming
Source: Nat Commun. 2019 Jul 24;10:3306. doi: 10.1038/s41467-019-11233-6 (PMC6656725; doi:10.1038/s41467-019-11233-6)
Supplement: Supplementary file 4 — Description of Additional Supplementary Files [file 41467_2019_11233_MOESM4_ESM.docx]

Description of Additional Supplementary Files

# File Name: Supplementary data 1: RNA-seq data

Description: List of differentially expressed genes between 1) Naïve and TIM CD8SP cells and 2) WT and *EomesTg* CD8SP cells.

# File Name: Supplementary data 2: ChIP-seq data

Description: List of differentially active enhancer and promoter regions between 1) Naïve and TIM CD8SP cells and 2) WT and *EomesTg* CD8SP cells.

# File Name: Supplementary data 3: ATAC-seq data

Description: List of differentially open enhancer and promoter regions between 1) Naïve and TIM CD8SP cells and 2) WT and *EomesTg* CD8SP cells.

# File Name: Supplementary data 4: Enhancer clusters

Description: List of enhancer clusters as defined in Fig 3a. RNA expression in naïve and TIM CD8SP cells for genes associated to these regions are also provided.

# File Name: Supplementary data 5: List of primers

Description: Sequences of primers used for ChIP-qPCR experiments.
