# Supplementary material for: EOMES interacts with RUNX3 and BRG1 to promote innate memory cell formation through epigenetic reprogramming
Source: Nat Commun. 2019 Jul 24;10:3306. doi: 10.1038/s41467-019-11233-6 (PMC6656725; doi:10.1038/s41467-019-11233-6)
Supplement: Supplementary file 2 — Peer Review [file 41467_2019_11233_MOESM2_ESM.pdf]

Reviewers' comments:

Reviewer #1 (Remarks to the Author):

The manuscript by Istaces et al entitled "EOMES interacts with RUNX3 and BRG1 during CD8 T cell development to promote memory traits through epigenetic reprogramming" describes the authors' attempt to unravel the molecular mechanisms underlying the conversion of naïve CD8+SP T cells in thymus to innate memory (TIM) cells. To this end, the authors established the transcriptome, epigenomic landscape and genomic profiles of Runx3 and Eomes binding in Balb/c naïve CD8+ SP T cells and TIM cells that represent a significant proportion of CD8 SP thymocytes in WT Balb/c mice. The authors also analyzed these parameters in thymic CD8 SP T cells derived from WT and EomesTg C57Bl mice, which have normally a small TIM population. They then claim that Eomes overexpression in thymocytes is sufficient to drive the TIM program in this mouse strain, and proceed to show that in TIM cells Eomes is recruited to genomic regions already occupied by Runx3 in naïve CD8 SP T cells and that Eomes, Runx3 and the chromatin remodeling factor BRG1 interaction brings about the epigenetic reprogramming of enhancer regions. However, the acquired epigenetic changes in TIM cells only partially resemble the active enhancer repertoire of central memory cells.
Although a lot of effort was invested in these experiments and in their analysis, the data is not presented in a clear coherent manner and the conclusions drawn are, in too many occasions, unclear and inaccurate as detailed below:

1. Page 5, line 4 from bottom: The authors show that Stat6 and Il4 deficient Balb/c mice lack TIM cells. However, although the authors emphasize later that Eomes is sufficient for driving TIM development, a previous study using Balb/c mice that lack Eomes in T cells (Eomesf/f :Cd4-Cre) (see ref. 13) showed only a 2-fold decrease in TIM cells, not a complete loss. This indicates that while Eomes is involved in driving TIM development, it is not sufficient, contrary to the authors' conclusions on pages 14 and 17. This discrepancy should be explained

2. Page 6, related to RNA-seq data in Fig. 1e: The authors mention several genes that are up- or down-regulated in sorted TIM (Eomeshigh) vs naïve (Eomeslow) CD8 cells. However, it is unclear how many genes are up- or down-regulated in TIM cells compared to naïve cells. The same holds for RNA-seq conducted on CD8 SP thymocytes of EomesTg and their WT counterparts. The authors should include a supplementary table listing all the differentially expressed genes and the fold-change of expression.

3. Pages 7-11, in relation to analysis of epigenomic landscape. Analysis of the genomic profile of several chromatin modifications (H3K27Ac, H3K4me1) and open chromatin (ATAC-seq) followed by identification of peaks common to more than one of the modifications is usually used to establish the enhancer landscape of a given cell type. While the authors have conducted all these experiments, they use single modifications for deduction of active enhancers. Moreover, it is unclear how many of the deduced enhancers share more than a single modification. The data should also be deposited in a clear manner. An additional enigma; why did the authors select the combined ATAC and H3K27Ac peaks for analysis of enriched TF binding motifs in Fig. 2e. It would have been more informative and scientifically sound to select for the analysis only peaks that are common to both modifications. Likewise, in Fig. 3c, it is unclear why only ATAC-seq peaks were used to determine enriched TF motifs. These faults should be corrected. Regarding the presented Chip-seq tracks, it would be very helpful to the reader if Fig. 4d also includes the tracks of H3k4me1, H3K27ac and ATAC and Fig. 6f includes the track of Runx3.

4. Page 9, in relation to Fig. 3a: In the clustering analysis of H3K27ac regions the authors show that cluster 3 is specifically enriched in TIM cells. Yet, they fail to indicate any interesting genes in this cluster and their potential relation to TIM cell function. The authors should add a supplementary Table

listing the genes in each of the figure six clusters, including their expression level in naïve CD8 SP vs TIM cells.

5. Page 9, in relation to Fig. 3c: the authors should comment/explain the result that a T-box motif is mainly enriched in cluster 2, common to central memory and TIM cells, but not in cluster 3, which is specific to TIM cells.

6. Page 9, the sentence "These results indicate that a major part of the epigenetic reprogramming observed in TIM cells is also encountered in conventional memory cells" is incorrect. Only 34% of the genes harboring H3K27Ac peaks in conventional memory cells are common to TIM cells; hardly a major part. Also, further in the same sentence "However, a large proportion of the events that take place in Ag-specific memory cells does not occur during unconventional memory formation". These faults should be corrected.

7. Page 11 bottom: The authors say "Importantly, almost 43% of these sites were also bound by RUNX3 in naïve CD8SP, suggesting that in his case, EOMES is recruited to regions that were associated beforehand with RUNX3". This is an interesting observation, but the authors should determine whether Runx3 regulates expression of Eomes in the transition from naïve CD8SP to TIM. It was previously reported that Runx3 drives Eomes expression during cytokine-induced differentiation of CD8 T cells (reference 19). In this regard, the question of whether generation of TIM from naïve CD8 cells is dependent on Runx3 remains open and should be discussed in the discussion section.

8. Page 12, lines 1-5. The authors used the BETA package to "infer genes that are directly regulated by EOMES" and presumably also genes directly regulated by Runx3, as shown in Fig. 4e. The authors claim that in contrast to EOMES, the regulatory potential of RUNX3 was rather weak, but they fail to determine the actual effect of Runx3 loss on TIM cell gene expression. Therefore, the authors' conclusion about the "weak regulatory potential" of Runx3 needs to be tested. In fact, it was previously shown that loss of Runx3 markedly affects peripheral memory CD8 cell development (ref 32) and tissue-resident memory CD8 cells (Milner J et al, Nature 552: 253, 2017).

9. Page 12, lines 10-11. The authors state that "chromatin accessibility as assessed by ATAC-seq was not significantly different between the two groups". It is not clear what is the significance of this distinction. Moreover, the sentences in lines 12-15 related to Fig. 4f, g lengthily describe, but without showing the actual data, regarding the percentage of peaks common to H3K4me1, H3K27ac, ATAC, Runx3 and EOMES, as well as what percentage of differentially expressed genes harbor all or part of these chromatin marks.

10. Page 13, in relation to Fig. 5c and supplementary Fig. 4. The authors describe the responsiveness of thymus CD8SP cells from WT and EomesTg mice to stimulation by PMA/ionomycin, IL12/IL18 (IFNg production) and IL-4 (expression of various markers) and indicate that the responses were "slightly but significantly increased" in the EomesTg cells. In fact, the increases are not slight. However, it seems strange that stimulation of WT CD8 SP thymocytes by IL12/IL18 but not by PMA/ionomycin induced IFNg production, while both stimuli induced IFNg in EomesTg CD8 SP cells. The more informative experiment to perform would be the measurement of PMA/ionomycin and IL-4 effects in naïve vs TIM cells from Balb/c mice, rather than on the whole population of thymic CD8SP cells.

11. Page 13 in relation to Fig. 5d-f: Please clarify the following: a) What is meant by "important proportion"? b) For some genes mentioned in Fig. 5d, f as "signature genes not differentially expressed in EomesTg vs WT" the claim that this could be due to "the fact that EOMES expression in EomesTg mice did not reach the levels observed in TIM cells from Balb/c mice (Fig. 5b)". This

explanation appears to be incorrect. For example, expression of Ccr4, Ccr9, Ifngr2, Sox4, Ikzf2, Egr1 and Egr2 is much higher in EomesTg vs WT than in TIM vs naïve (Fig. 5f). In addition, while up-regulated in both EomesTg vs WT and TIM vs naïve, Cxcr3 is expressed at much higher levels in EomesTg vs WT than in TIM vs naïve (Fig. 5b).

12. Page 15 line 6: The authors determined co-immunoprecipitation of EOMES and BRG1 in 293 cells transfected with an EOMES expression vector. It would have been physiologically more meaningful to determine this co-immunoprecipitation in TIM cells from Balb/c mice, which express high level of Eomes.

13. Page 16 in relation to Fig. 6a, c: Regarding ATAC and H3K27Ac peaks, a comparison between the peaks in TIM and EomesTg cells is missing and should be included.

14. Page 17, lines 8-10: The authors conclude: "Herein, we show that a single transcription factor, EOMES, is sufficient to drive acquisition of a memory associated phenotype and of transcriptional and epigenetic profiles in developing CD8SP thymocytes". This conclusion is inaccurate. The fact that Eomes is recruited in TIM to many genomic regions already occupied by Runx3 in naïve cells, supports the conclusion that Eomes is not sufficient. It could very well be that cooperation between both Runx3 and Eomes is required. The authors should address these issues.

15. Page 18, second paragraph: The authors mention the importance of T-box TFs in cell fate decisions and in many organogenesis aspects. In the context of memory CD8 development it was shown that both Eomes and T-bet are essential for generation of peripheral memory CD8 cells (ref. 20). Moreover, absence of both these T-box TF, but not each one alone, changes the fate of CD8 cells following viral infection, leading to development of a lethal inflammation (Intlekofer et al, Science 321: 408, 2008). In the present experiments, the authors show that T-bet expression is increased in TIM vs naive CD8 SP cells as well as in EOMESTg vs WT cells. It is therefore possible that T-bet occupies many of the sites occupied by Eomes in TIM cells. Hence, T-bet may also participate with Eomes and Runx3 in promoting the development of TIM cells from naive CD8 cells. This possibility should be discussed.

Minor:

1. Experiments were done with mouse thymocytes. Therefore, all nomenclature of proteins should be according to mouse, not human. For example, it should be written "Runx3-bound" and not "RUNX3-bound" enhancers. Same holds for Ikzf1, Ikzf3 and not IKZF1, IKZF3 etc, throughout the paper.
2. Page 5, please clarify the sentence "Cell heterogeneity within EOMESlo showed more complex patterns: subsets were mainly defined by CD62L, CD49d and CD103 expression".
3. Page 7, a reference should be added after the sentence "Differentiation of CD8+ T lymphocytes into memory cells is accompanied by dynamic changes in the chromatin landscape of promoters and enhancers".
4. Page 8, bottom, please clarify the sentence "We identified differentially active enhancers that were common to both models. This allowed us to define consensus sets of enhancer regions that are more (1105 regions) or less (1241 regions) active in conventional memory cells as compared to their naïve counterparts". What is the meaning of more or less active?
5. Page 10, why was RIME conducted in activated primary CD8 T cells and not in TIM cells?
6. It is unclear what was done in Fig. 7d.
7. Figure 6a, the number of ATAC regions is the same as in Figure 2e, is it a mistake?

Reviewer #2 (Remarks to the Author):

Istaces et.al. use RNA-seq and ATAC-seq techniques to investigate the epigenetic landscape of innate-like memory T cells that form in response to cytokines. They perform comparisons with naïve T cells and 'true memory' T cells, showing that although innate-like memory cells possess many of the characteristics of antigen-experienced memory, they do not fully replicate the programming induced in conventional memory cells. Furthermore, they identify Eomes- previously shown by many investigators as strongly upregulated in innate-like memory cells- as a critical factor in programming innate-like memory T cells. RUNX3 and BRG1 were also found to play direct roles in remodeling. The manuscript is well written, the data interesting and compelling, and the methods and statistical analysis are well described.

Although the involvement of Eomes is not surprising, the manuscript establishes that it's sufficient to produce many features of innate-like memory and describes how Eomes enforces it influence. The authors also further develop the global programming network involved in establishment of functional memory in the absence of antigen exposure and highlight new players in this process. These are important steps forward in our understanding of memory T cell formation and the work will be of interest to the field. I have only minor comments to improve the manuscript.

In Figure 3, it seems like clusters 1,3, and 4 (which differ between TIM and conventional memory) should be highlighted more. What do the authors think is the significance of cluster 3?

In Figure 5c and Supplemental Fig.4, it would be helpful to include functional responses in BALB/c mice for comparison as was done in Figure 5b for phenotype. If responses are weak in Eomes^Tg by comparison, this may point to the need for additional factors to achieve the enhanced function observed in TIM.

As related to Figure 6, what would comparisons between Eomes^Tg and the sorted TIM look like?

What is the significance of BRG1 based on prior work? More background leading into Figure 7 would be helpful and summary discussion after the data is presented.

Reviewer #3 (Remarks to the Author):

This research focuses on the molecular regulation of thymic innate memory T cell (TIM) development. The authors define potential lineage relationships based on thymocyte phenotypes that occur within Eomeshi and Eomeslo cells, demonstrate the presence of TIM in WT mice and infer their potential developmental origin. In addition, they use multiple genome-wide approaches and demonstrate that TIM cells and conventional central memory CD8 T cells (TM) exhibit extensive similarities and differences in gene expression, which correlate with chromatin accessibility, histone modifications and transcription factor (TF) utilization within cis-regulatory regions. Specifically, the authors identify strong enrichment of TF motifs encoding T-box protein binding sites within TIM-active regions, and conclude that the TF Eomes directs this transcriptional program by promoting transcription and chromatin structure modifications at these sites in TIM cells. Using pull-down strategies, they provide evidence for association of Eomes with Runx3 and Brg1/Smarca4 proteins, which is consistent with both the enrichment of Runx-TF motifs in cis-regulatory regions that are accessible/active within TIM cells relative to naïve cells, and that binding of Runx3 and Eomes overlaps extensively within genes that are expressed in TIM cells. Therefore, the authors conclude that Eomes is recruited mainly to

distal cis-regulatory regions that are pre-established with Runx3, regulates their activity and governs transcriptional output of their linked genes. Using a CD4-Cre driven disruption of the Smarca4 allele, the authors provide evidence that Brg1-deficiency impairs multiple parameters of TIM development.

The manuscript contains a wealth of genetic and genomic data that clarifies the development of TIM cells, and most of the conclusions are supported well by the data and their analyses. Overall, the text is written clearly, although some improvements will make it more accessible to readers. The strongest part of the study are the analyses of Eomes and Runx3 in the contexts of chromatin structure, gene expression and cell development. The conclusions regarding the exact role(s) of Smarca4 and its biochemical interactions with Eomes are less well developed, and the conclusions/interpretations relating to these experiments should de-emphasized.

Major points:
1. There really is not enough evidence to indicate that Eomes is part of the SWI/SNF complex as claimed in the discussion: "We showed that EOMES can be found in complexes containing members of the SWI/SNF machinery, histone deacetylases and their associated DNA-binding ATPase CHD4." In fact, what the authors have shown is that in fixed chromatin, co-immunoprecipitates that are recovered with Eomes-specific antibodies include some (but apparently not all) members of a canonical BAF (Brg1-associated factors) complex. This analysis did not exclude that some of the co-associated proteins were associated by DNA bridging (which might also explain the association of Runx3 with Eomes in these experiments). Although the authors also demonstrate a co-association of Eomes with Brg1 in co-transfection assays, the observed association appears to be weak despite the extreme overexpression conditions, and there were not other positive control blots showing pull-down of other BAF-complex subunits. To conclusively confirm the hypothesis that Eomes is part of a BAF-complex in T cells would require additional extensive biochemical characterization, including isolation of native BAF complexes and demonstrating that Eomes is a part of it. The present findings are interesting, but are still underdeveloped and the conclusions should be adjusted accordingly.

2. The authors postulate (in the discussion) that their results "strongly suggest that EOMES allows the recruitment of the SWI/SNF machinery", but their results show that Eomes expression is reduced in TIM in the thymus of Smarca4-deficient mice (and later in the spleen as well), which suggests that Brg1/Smarca4 acts before/upstream of Eomes. This result does not exclude that Eomes also promotes recruitment of Brg1/Smarca4, but without the proper epistatic experiments, this cannot be known for sure. It might be prudent to leave open both possibilities when discussing the results.

3. Although the transgenic Eomes gain-of-function experiments are very convincing, the strength of the conclusion that Eomes promotes the transcriptional activity of its bound cis-regulatory regions is diminished in the absence of the Eomes loss-of-function experiments. An important set of experiments that was not presented but that would be of interest is how Eomes-deficiency affects the transcription and at least some aspects of the chromatin landscape of TIM cells.

4. Although it is logical by extension, the strength of the conclusion that Runx3 is involved in the recruitment of Eomes to specific cis-regulatory regions is diminished without examining whether Runx3 is required for this process, and whether development of TIM cells is impaired in the absence of Runx3. These are challenging experiments because thymic Runx3-disruption will be difficult to achieve without dramatically impairing overall CD8 T cell development, although haploinsufficiency of Runx3 could be telling.

Specific comments/suggestions:
1. There are some places in this paper that describe TIM phenotype with ambiguous descriptions that may lead to misunderstanding of readers to believe it is an earlier stage of typical memory phenotype. For instance, in abstract, "naïve CD8 single positive (SP) thymocytes may already acquire phenotypic and functional characteristics of memory…". In this sentence, "already" suggests that in the future

those cells can become typical memory cells. Moreover, in the introduction, it was described: "… naïve CD8 T cells in a lymphopenic environment undergo conversion to memory phenotype CD8 T cells". Those cells could be better described as "memory-like" or "virtual memory", to avoid the confusion of readers. I suggest use unified description for TIM phenotype and avoid ambiguity of typical memory and other types of memory like phenotype throughout the paper.

2. It would be helpful introduce the potential role of TIM cells in the introduction (which is currently in the discussion).

3. For the spanning-tree analysis for flow cytometry data, the multi-colors are too diverse, making it a bit difficult to discern which population(s) has higher/lower expression of genes. In addition, there are some more up-to-date unsupervised flow cytometry clustering methods, as described and compared in this review paper:https://www.ncbi.nlm.nih.gov/pubmed/27992111. It seems that FlowSOM / X-shift might have better performance than SPADE especially for rare populations such as TIM in the setting of B6 mice.

4. Some of the conclusions regarding figure 4c are unclear: (1) "Up to 65% of these EOMES peaks overlapped with RUNX3 (Fig 4c)." When we did the math based on the presented Venn diagrams, we get slightly lower values. Can you please clarify how your numbers were calculated? (2) The authors demonstrate a significant (albeit less strong) probability of Runx-motifs occurring within proximity to Eomes binding sites that do not appear to have overlap with Runx3 ChIP-seq peaks (Fig 4C, the 751 Eomes binding sites), but this is not explained carefully, which could confuse some readers. Does this arise because of potential Runx3 binding sites that were not called as peaks, or because of how the binding site intersections between Runx3 and Eomes regions were computed? If the latter, the current analysis might underestimate the actual co-localization of Runx3/Eomes binding.

Istaces *et al* (NCOMMS-19-01199)

"EOMES interacts with RUNX3 and BRG1 during CD8 T cell development to promote memory traits through epigenetic reprogramming"

Point-by-point response to the referees' comments

Throughout the document, reviewer's comments are in italic and between quotation marks, followed by our responses in bold font. Modifications within the manuscript are highlighted in green. The order of several figures was modified according to these changes: we use this new figure numbering in our response. Some figures provided only in this point-by-point response (Fig. R) are not primarily meant to figure in a final published version but are there to better address reviewers' questions.

We want to thank the reviewers for their attentive reading of the manuscript, and for their constructive input. When similar points were raised by more than one reviewer, we refer you to the first time we addressed it in order to avoid redundancy. If you still feel your remarks have not been addressed specifically enough, please let us know in order to further discuss any concern you might have.

<u>Reviewer #1:</u>

Point 1: Page 5, line 4 from bottom: The authors show that Stat6 and Il4 deficient Balb/c mice lack TIM cells. However, although the authors emphasize later that Eomes is sufficient for driving TIM development, a previous study using Balb/c mice that lack Eomes in T cells (Eomesf/f :Cd4-Cre) (see ref. 13) showed only a 2-fold decrease in TIM cells, not a complete loss. This indicates that while Eomes is involved in driving TIM development, it is not sufficient, contrary to the authors' conclusions on pages 14 and 17. This discrepancy should be explained"

To the best of our knowledge, Ref13 (*Gordon et al. Requirements for eomesodermin and promyelocytic leukemia zinc finger in the development of innate-like CD8+ T cells. J. Immunol 2011*) did not

involve WT and *Eomesf/f:Cd4-Cre* Balb/c mice and this has not been reported by others. Gordon *et al* mainly studied WT and Y145F C57BL/6 mice that lack or not *Eomes* expression in T cells. They clearly show that in this model, the increase in CD44^{hi}CD122^{hi} CD8SP thymocytes was strongly Eomes-dependent (from 66% to 5% among CD8SP thymocytes, Fig. 5). We don't argue with the fact that the CD44^{hi}CD122^{hi} CD8SP cells do not completely disappear in C57BL/6 *Eomesf/f:Cd4-Cre* mice (from 3% to 1.5% among CD8SP thymocytes, Fig 6) but in our opinion, the very low frequency of residual memory phenotype cells could be due to circulating conventional memory cells. Furthermore, the dependency of T$_{IM}$ cells on Eomes is difficult to assess in these conditions.

However, we agree that our statement that Eomes is sufficient to drive T$_{IM}$ development is inaccurate and modified the manuscript accordingly (see response to comment 11).

2. *"Page 6, related to RNA-seq data in Fig. 1e: The authors mention several genes that are up- or down-regulated in sorted TIM (Eomeshigh) vs naïve (Eomeslow) CD8 cells. However, it is unclear how many genes are up- or down-regulated in TIM cells compared to naïve cells. The same holds for RNA-seq conducted on CD8 SP thymocytes of EomesTg and their WT counterparts. The authors should include a supplementary table listing all the differentially expressed genes and the fold-change of expression."*

The numbers of up- or downregulated genes in sorted T$_{IM}$ and naïve (Fig. 1e), as well as in CD8SP thymocytes of *Eomes^{Tg}* and their WT counterparts (Fig. 5d) have been added on the respective volcano plots. The lists of differentially expressed genes with the fold-change expression and FDR have been added as supplementary data (Supplementary Data 1).

3."Pages 7-11, in relation to analysis of epigenomic landscape. Analysis of the genomic profile of several chromatin modifications (H3K27Ac, H3K4me1) and open chromatin (ATAC-seq) followed by identification of peaks common to more than one of the modifications is usually used to establish the enhancer

landscape of a given cell type. While the authors have conducted all these experiments, they use single modifications for deduction of active enhancers. Moreover, it is unclear how many of the deduced enhancers share more than a single modification. The data should also be deposited in a clear manner. An additional enigma; why did the authors select the combined ATAC and H3K27Ac peaks for analysis of enriched TF binding motifs in Fig. 2e. It would have been more informative and scientifically sound to select for the analysis only peaks that are common to both modifications. Likewise, in Fig. 3c, it is unclear why only ATAC-seq peaks were used to determine enriched TF motifs.

These faults should be corrected. Regarding the presented Chip-seq tracks, it would be very helpful to the reader if Fig. 4d also includes the tracks of H3k4me1, H3K27ac and ATAC and Fig. 6f includes the track of Runx3."

Regarding the enhancer landscape, we used H3K4me1-ChIP-seq peaks to map enhancers but, as reported by others (eg. Fu et al, Nucleic Acid Research 2018, https://doi.org/10.1093/nar/gky753). We found that differential analysis for this modification was not sufficiently reliable due to the low intensity and broadness of the peaks. Hence, analysis of H3K27Ac-ChIP-seq peak intensities within these H3K4me1+ regions was used to assess enhancer activity. This reference (ref 21) has now been added to justify the approach.

As suggested, we have now added the lists of differentially active promoters and enhancers as supplementary data (Supplementary Data 2).

For the enrichment analysis of TF binding motifs we used two complementary approaches. Most articles now solely rely on differential ATAC peaks in enhancers for this purpose (eg Scott-Browne et al, Immunity 2016, Yu B et al, Nat immunol 2017). This was the approach used in Fig. 2e-f. Other articles rely on open regions (whether differentially open or not) within differentially active enhancers (see Lavin et al Cell 2014: *"Because H3K4me1- marked enhancers may span*

***several kilobases, but TF motifs usually occupy no more than a dozen bases, we generated ATAC-seq peaks corresponding to the same tissue-resident macrophages to narrow our search regions to the likely site of TF binding"*). This approach was used in Fig. 3d as He *et al* (one of the two public data set used) did not perform ATAC-Seq on conventional memory CD8 T cells.**

To clarify this point, this has now been rephrased in the result section as follows:

"To narrow our search for binding motifs, we focused our analysis on the centre of ATAC peaks located in these sets of enhancer regions (Fig. 3d)"

Both approaches were found to yield very similar results for motif analysis when naïve and T_{IM} cells were compared. There was a good correlation between ATAC and H3K27Ac activity in enhancer regions (see Fig. R1) and when we combined both criteria (differential H3K27Ac activity AND ATAC intensities) as suggested, we confirmed our results (Fig. R2). However, it substantially decreases the number of regions for motif analysis (125 and 130 UP and DOWN regions) and we thought it would be more informative to keep all DOR in Fig. 2f.

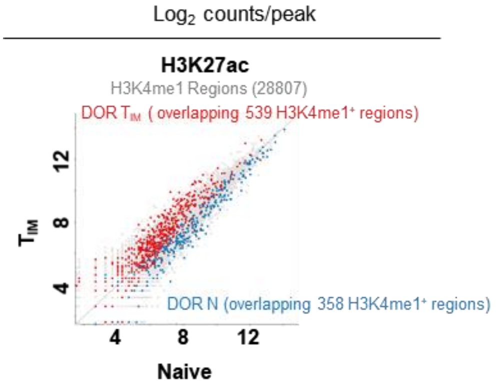

Fig. R1: Quantification of H3K27Ac intensities within enhancers (defined as H3K4me1+ regions) from naïve and T_{IM} cells. Differentially open regions (based on ATAC peaks within these enhancers) are highlighted in Red (T_{IM}-specific) and blue (Naïve-specific).

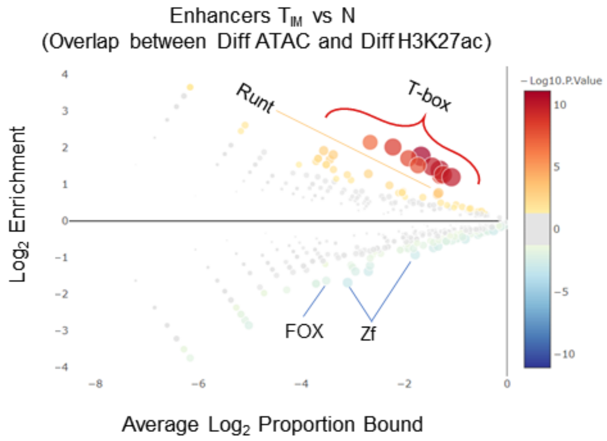

Fig. R2: CiiiDER analysis of putative transcription factors motifs in enhancer-bound DOR that are also differentially active for H3K27Ac. Transcription factors are colored according to the p-value of their gene coverage and whether they are over (red) or under (blue) represented in T_{IM} cells. The size of each point is also proportional to Log10 p-value.

Fig. 4d: we agree that this adds to the clarity of the message/figure: the tracks of H3k4me1-ChIP-, H3K27ac-ChIP- and ATAC-seq have now been added (for naïve and T_{IM}).

Fig. 6f: idem: the tracks for Runx3 have also been added (for naïve and T_{IM}).

4. *"Page 9, in relation to Fig. 3a: In the clustering analysis of H3K27ac regions the authors show that cluster 3 is specifically enriched in TIM cells. Yet, they fail to indicate any interesting genes in this cluster and their potential relation to TIM cell function. The authors should add a supplementary Table listing the genes in each of the figure six clusters, including their expression level in naïve CD8 SP vs TIM cells."*

- **A supplementary file (Supplementary Data 4) listing all genes corresponding to each of the six clusters and their expression levels in naïve/T_{IM} cells has been added. We also added a figure with the FC in conventional memory and T_{IM} cells of genes associated with each cluster (Fig. 3b).**
- **Regarding cluster 3, unfortunately, we did not identify obvious genes involved in biologically-relevant pathways (Fig. 3c). We highlight a few genes (*Otulin, Tmem2, Gpr155*) that were differentially expressed in T_{IM} cells and for which absolute expression >5 FPKM. We show**

gene-ontology analysis for clusters 3 and 6 (Fig. 3c) and wrote in the result section: "This analysis did not reveal biologically relevant pathways for the limited numbers of genes associated with T_{IM}-specific clusters 3 and 6".

5. *"Page 9, in relation to Fig. 3c: the authors should comment/explain the result that a T-box motif is mainly enriched in cluster 2, common to central memory and TIM cells, but not in cluster 3, which is specific to TIM cells."*

   The observation that the p value for T-box motif enrichment is less significant in cluster 3 is probably related to the fact that the number of regions (111) in this cluster is limited. Indeed the overlap with EOMES ChIP-Seq peaks (Fig. 3e) was comparable for both clusters. We now point this out in the manuscript, in the result section: "The smaller set of unconventional memory-specific enhancers (cluster 3) also harboured T-box motifs, although to a lesser extent than in cluster 2"**..**

6. *"Page 9, the sentence "These results indicate that a major part of the epigenetic reprogramming observed in TIM cells is also encountered in conventional memory cells" is incorrect. Only 34% of the genes harboring H3K27Ac peaks in conventional memory cells are common to TIM cells; hardly a major part. Also, further in the same sentence "However, a large proportion of the events that take place in Ag-specific memory cells does not occur during unconventional memory formation". These faults should be corrected."*

We agree that only 34% of the genes harboring H3K27Ac peaks in conventional memory cells are common to T_{IM} cells, which is indeed explained in the sentence "However, a large proportion of the events that take place in Ag-specific memory cells does not occur during unconventional memory formation". What we meant by "These results indicate that a major part of the epigenetic reprogramming observed in T_{IM} cells is also encountered in conventional memory cells" is that among the regions that are more active in T_{IM} cells as compared to naïve CD8SP (clusters 2+3 :453 regions), 75% (cluster 2 only : 342 regions) are also more active in conventional memory compared to naïve cells.

Likewise, 84% (297 out of 351) of enhancer regions that are less active in T_{IM} cells are also less active in conventional memory compared to naïve cells. We rephrased this in the manuscript :

> "These results suggest that a major part of the epigenetic reprogramming observed in T_{IM} cells is also encountered in conventional memory cells (i.e. 75% of enhancer regions that are more active and 84% of enhancer regions that are less active in T_{IM} compared to naïve CD8SP thymocytes follow the same differential pattern between T_M and their naïve counterparts). However, a large proportion of the events that take place in Ag-specific memory cells (clusters 1 and 4) does not occur during unconventional memory formation."

7. *"Page 11 bottom: The authors say "Importantly, almost 43% of these sites were also bound by RUNX3 in naïve CD8SP, suggesting that in his case, EOMES is recruited to regions that were associated beforehand with RUNX3". This is an interesting observation, but the authors should determine whether Runx3 regulates expression of Eomes in the transition from naïve CD8SP to TIM. It was previously reported that Runx3 drives Eomes expression during cytokine-induced differentiation of CD8 T cells (reference 19). In this regard, the question of whether generation of TIM from naïve CD8 cells is dependent on Runx3 remains open and should be discussed in the discussion section."*

This is an interesting point as we observed T_{IM}-specific RUNX3 recruitment close to the *Eomes* locus. We now provide this data as Supplementary Fig. 3 and added the following paragraph in the discussion section:

"In addition, we observed T_{IM}-specific recruitment of RUNX3 in several locations around the *Eomes* locus (Supplementary Fig. 3). It suggests that RUNX3 could participate to EOMES upregulation in this setting as described in polyclonally activated CD8 T cells[19]".

8. *"Page 12, lines 1-5. The authors used the BETA package to "infer genes that are directly regulated by EOMES" and presumably also genes directly*

regulated by Runx3, as shown in Fig. 4e. The authors claim that in contrast to EOMES, the regulatory potential of RUNX3 was rather weak, but they fail to determine the actual effect of Runx3 loss on TIM cell gene expression. Therefore, the authors' conclusion about the "weak regulatory potential" of Runx3 needs to be tested. In fact, it was previously shown that loss of Runx3 markedly affects peripheral memory CD8 cell development (ref 32) and tissue-resident memory CD8 cells (Milner J et al, Nature 552: 253, 2017)."

We agree with this comment and de-emphasized the message as follows: "Of note, we also observed a significant regulatory potential for RUNX3 on T_{IM}-specific genes".

We should also stress that our data in no way contradict the 2016 Immunity paper (Wang et al.) and on the contrary illustrates that *Runx3* plays a role (although indirect) in the formation of CD8 memory cells both after antigen encounter or independently of the antigen under the sole influence of cytokines. We also already emphasize the important regulatory role of *Runx3* in the acquisition of memory features in that CD44, Ly6C, CD124, T-BET and EGR2 expressions are unchanged in CD4SP thymocytes upon *Eomes* ectopic expression, and suggest that this might be related to the absence/low levels of RUNX3 in these cells.

9. *"Page 12, lines 10-11. The authors state that "chromatin accessibility as assessed by ATAC-seq was not significantly different between the two groups". It is not clear what is the significance of this distinction. Moreover, the sentences in lines 12-15 related to Fig. 4f, g lengthily describe, but without showing the actual data, regarding the percentage of peaks common to H3K4me1, H3K27ac, ATAC, Runx3 and EOMES, as well as what percentage of differentially expressed genes harbor all or part of these chromatin marks."*

To improve clarity, we simplified the text related to Fig. 4g as follows:

"In enhancers and to a lesser extent in promoter regions of upregulated genes, we observed an increase in histone marks and ATAC signals around EOMES binding sites in T_{IM} cells as compared to their naïve counterparts (Fig. 4g). In sharp contrast,

in regulatory regions associated with downregulated EOMES target genes, the same parameters were not modulated except for a minor decrease in H3K27ac levels around EOMES peaks in enhancer regions".

10. *"Page 13, in relation to Fig. 5c and supplementary Fig. 4. The authors describe the responsiveness of thymus CD8SP cells from WT and EomesTg mice to stimulation by PMA/ionomycin, IL12/IL18 (IFNg production) and IL-4 (expression of various markers) and indicate that the responses were "slightly but significantly increased" in the EomesTg cells. In fact, the increases are not slight. However, it seems strange that stimulation of WT CD8 SP thymocytes by IL12/IL18 but not by PMA/ionomycin induced IFNg production, while both stimuli induced IFNg in EomesTg CD8 SP cells. The more informative experiment to perform would be the measurement of PMA/ionomycin and IL-4 effects in naïve vs TIM cells from Balb/c mice, rather than on the whole population of thymic CD8SP cells."*

As suggested, we performed additional experiments to provide PMA/Iono-stimulated Balb/c thymocytes from Balb/c mice (Fig. 5c). We now show IFNγ production data side by side for $CD3^+$CD8SP thymocytes from C57BL/6 WT and *$Eomes^{Tg}$* mice, along with $EOMES^{lo}$ and $EOMES^{hi}$ CD8SP thymocytes from WT Balb/c mice. It indicates that production in $Eomes^{Tg}$ cells remains limited as compared to T_{IM} cells, justifying the "*slightly but significantly increased*".

Regarding the Supplementary Fig. 5 (*ex vivo* stimulation with rIL-4), we also submit to you the same read-outs presented for $CD3^+$CD8SP thymocytes from C57BL/6 WT and *$Eomes^{Tg}$* mice, along with $EOMES^{lo}$ and $EOMES^{hi}$ CD8SP thymocytes from WT Balb/c mice (Fig. R3). They show that changes observed in T_{IM} after rIL-4 *ex vivo* stimulation are qualitatively recapitulated in *$Eomes^{Tg}$* mice. However, we are not sure that these data should be added in the Supplementary Fig. 5 as we observed a general shift of EOMES both in naïve and T_{IM} even if both populations remained discernable after stimulation in a CD44-EOMES plot.

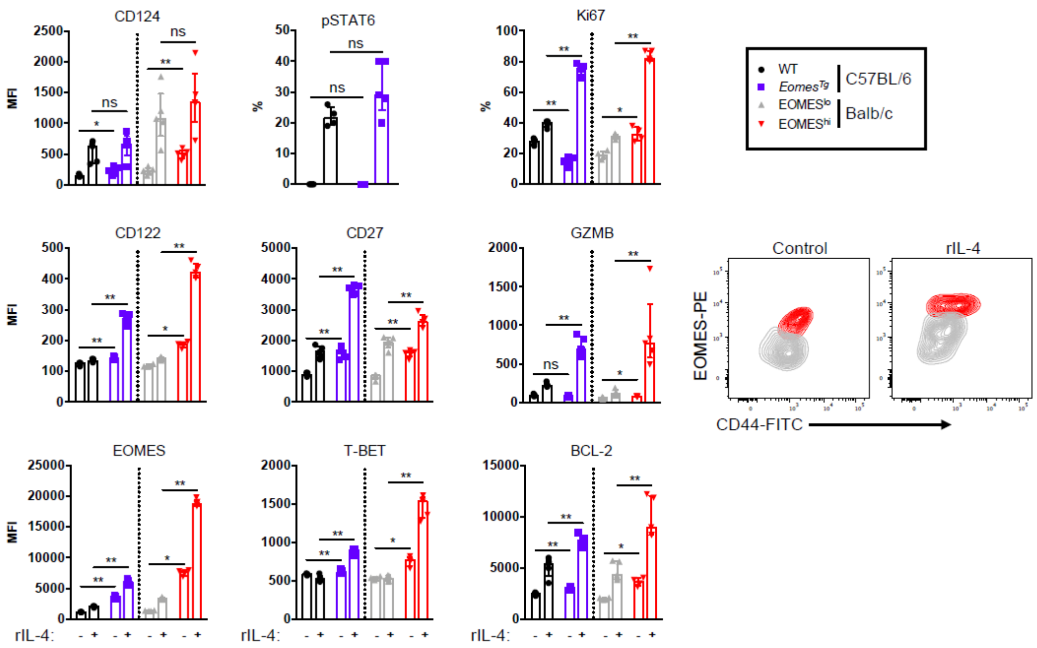

Fig. R3: rIL4 Ex vivo stimulation of thymocytes from C57BL/6, EomesTg and Balb/c mice (related to Fig S4).

11. *"Page 13 in relation to Fig. 5d-f: Please clarify the following: a) What is meant by "important proportion"? b) For some genes mentioned in Fig. 5d, f as "signature genes not differentially expressed in EomesTg vs WT" the claim that this could be due to "the fact that EOMES expression in EomesTg mice did not reach the levels observed in TIM cells from Balb/c mice (Fig. 5b)". This explanation appears to be incorrect. For example, expression of Ccr4, Ccr9, Ifngr2, Sox4, Ikzf2, Egr1 and Egr2 is much higher in EomesTg vs WT than in TIM vs naïve (Fig. 5f). In addition, while up-regulated in both EomesTg vs WT and TIM vs naïve, Cxcr3 is expressed at much higher levels in EomesTg vs WT than in TIM vs naïve (Fig. 5b)."*

   a) **We modified this paragraph for clarity:**

      "We identified 254 up- and 491 down-regulated genes upon ectopic expression of Eomes (FC>2, FdR<0.05, Fig. 5d and Supplementary Data 1). Globally, naïve- and T_{IM}–specific genesets were significantly enriched in WT and *Eomes^{Tg}* CD8SP thymocytes, respectively (Fig. 5e)".

b) **We have observed dose-dependent effects of EOMES on several markers and functional responses when comparing WT, *Eomes*$^{WT/Tg}$, and *Eomes*$^{Tg/Tg}$ mice (now provided as Supplementary Fig. 7). We cannot exclude that this might explain the absence of upregulation of some genes in *Eomes*^{Tg} compared to T_{IM} (i.e. we could have seen eventually the upregulation or downregulation of some markers in *Eomes*^{Tg} comparable to what is observed in T_{IM} if EOMES levels were the same in both populations). A good example is CD122 which is not upregulated in *Eomes*$^{WT/Tg}$ but slightly upregulated in *Eomes*$^{Tg/Tg}$ mice (the latter being shown in the paper), and way more upregulated in T_{IM}. Nevertheless we agree with the comment and added alternative explanations in the result section:**

"These discrepancies could be related to the fact that EOMES expression in *Eomes*^{Tg} mice did not reach the levels observed in T_{IM} cells from Balb/c mice (Fig. 5b). We indeed observed dose-dependent effects of EOMES on the expression of several markers when comparing WT, *Eomes*$^{WT/Tg}$, and *Eomes*$^{Tg/Tg}$ C57BL/6 mice (Supplementary Fig. 7). In addition, STAT6 activation in T_{IM} cells from Balb/c could play a role independently of its effect on *Eomes* induction. Conversely, the observation that CXCR3 increases in a dose-dependent fashion while its expression level is lower in T_{IM} compared to *Eomes*^{Tg} CD8SP thymocytes (Fig. 5b) suggests that factors opposing EOMES activating effects could be present in T_{IM} and not in *Eomes*^{Tg} CD8SP thymocytes".

12. *"Page 15 line 6: The authors determined co-immunoprecipitation of EOMES and BRG1 in 293 cells transfected with an EOMES expression vector. It would have been physiologically more meaningful to determine this co-immunoprecipitation in TIM cells from Balb/c mice, which express high level of Eomes."*

We are limited in the total number of T_{IM} cells that we can obtain for this kind of experiment. As an alternative, we performed ChIP-qPCR for EOMES and BRG1 in *Eomes*^{Tg} cells that support the notion that these 2

proteins are recruited to the same genomic loci. These data are now shown in Fig. 7b. We also modified our conclusions accordingly:

"As shown in Fig 7a, BRG1 and EOMES co-precipitated, suggesting that these proteins can be found in the same molecular complexes. We further show that EOMES binding to regulatory regions associated with *Il2rb, Cxcr3, Kdm5b, Samd3* and *Stat4* loci in $Eomes^{Tg}$ CD8SP thymocytes was accompanied by increased BRG1 recruitment (Fig. 7b). This result suggests that recruitment of BRG1-containing complexes along with EOMES could contribute to CD8SP thymocyte differentiation into T_{IM} cells."

13. *"Page 16 in relation to Fig. 6a, c: Regarding ATAC and H3K27Ac peaks, a comparison between the peaks in TIM and EomesTg cells is missing and should be included."*

This comparison is now available for ATAC in Supplementary Fig. 8 and presented in the result section:

"We compared the changes in chromatin accessibility that occur in enhancers of T_{IM} and $Eomes^{Tg}$ CD8SP cells (Supplementary Fig. 8). We observed that about half of the DOR of T_{IM} cells displayed the same behavior in $Eomes^{Tg}$ cells. Motif analysis in T_{IM}-specific cluster 1 did not reveal enrichment for unique TF that could contribute to T_{IM} development independently of EOMES. Furthermore, the overlap of clusters 1 and 2 regions with EOMES and RUNX3 ChIP-Seq data was found to be comparable.".

We performed the same analysis for H3K27Ac peaks (Fig. R4) but did not include this data in the manuscript as the message was globally similar.

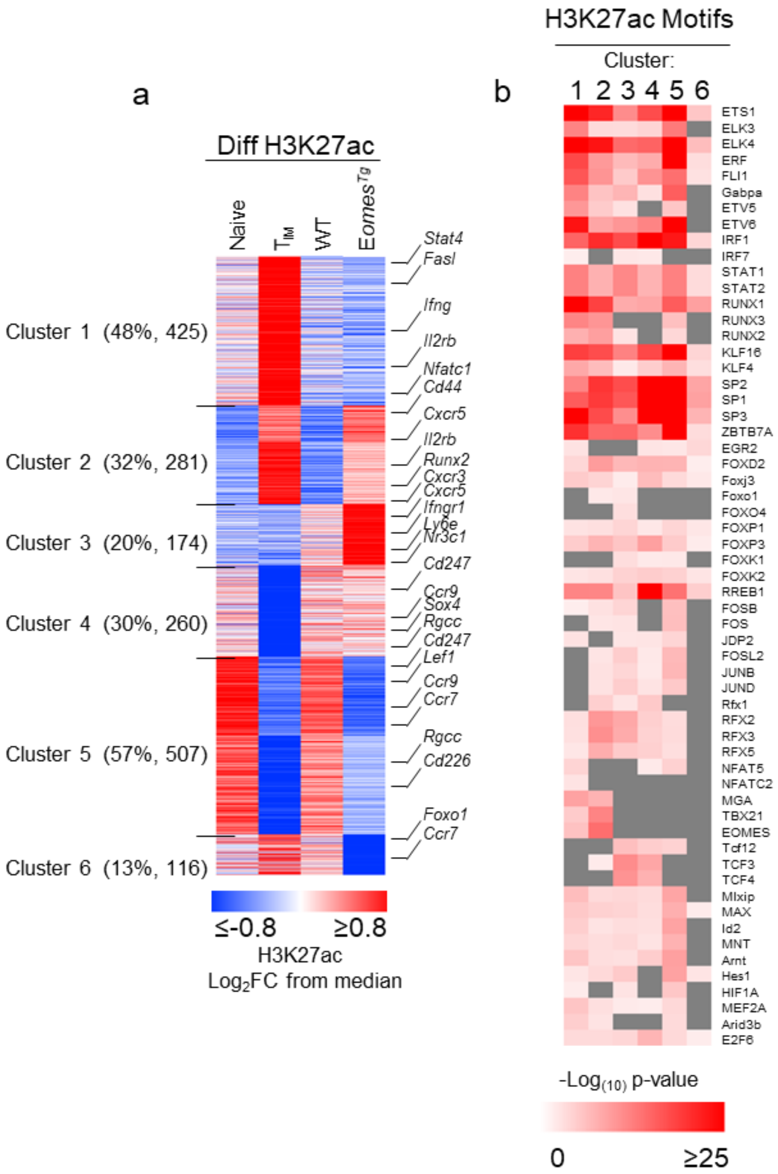

Fig R4: a, Clusters of differentially active enhancers in CD8SP (Naive,TIM, WT and *Eomes*^{Tg}) based on their H3K27ac intensity. b, Motif enrichment analysis of clusters shown in a.

14. *"Page 17, lines 8-10: The authors conclude: "Herein, we show that a single transcription factor, EOMES, is sufficient to drive acquisition of a memory associated phenotype and of transcriptional and epigenetic profiles in developing CD8SP thymocytes". This conclusion is inaccurate. The fact that Eomes is recruited in TIM to many genomic regions already occupied by Runx3 in naïve cells, supports the conclusion that Eomes is not*

sufficient. It could very well be that cooperation between both Runx3 and Eomes is required. The authors should address these issues."

We agree and rephrased to correct this:

"Herein, we show that **the ectopic expression** of a single transcription factor, EOMES, is sufficient to drive the acquisition of a memory-associated phenotype and of transcriptional and epigenetic profiles in developing CD8SP thymocytes"**…**

And in the final conclusion:

"Ectopic expression of EOMES in developing CD8SP thymocytes is sufficient to drive this program and acts in a BRG1-dependent fashion".

As stated above, it is not our intention to downplay *Runx3* regulatory role, quite the contrary: we suggest that binding of RUNX3 could be critical for EOMES recruitment to important regulatory elements in the context of T_{IM} development. Parts of the manuscript have been rephrased accordingly.

15. *"Page 18, second paragraph: The authors mention the importance of T-box TFs in cell fate decisions and in many organogenesis aspects. In the context of memory CD8 development it was shown that both Eomes and T-bet are essential for generation of peripheral memory CD8 cells (ref. 20). Moreover, absence of both these T-box TF, but not each one alone, changes the fate of CD8 cells following viral infection, leading to development of a lethal inflammation (Intlekofer et al, Science 321: 408, 2008). In the present experiments, the authors show that T-bet expression is increased in TIM vs naive CD8 SP cells as well as in EOMESTg vs WT cells. It is therefore possible that T-bet occupies many of the sites occupied by Eomes in TIM cells. Hence, T-bet may also participate with Eomes and Runx3 in promoting the development of TIM cells from naive CD8 cells. This possibility should be discussed."*

We added the following paragraph in the discussion section:

"Of note, expression of T-BET was found to be upregulated in T_{IM} cells. While C57Bl/6 *Tbx21*$^{-/-}$ mice dispay increased proportion of T_{IM} cells through enhancement of NKT2-derived IL-4[35], it is possible that this TF contributes to their development and function along with EOMES".

“Minor:

1. Experiments were done with mouse thymocytes. Therefore, all nomenclature of proteins should be according to mouse, not human. For example, it should be written "Runx3-bound" and not "RUNX3-bound" enhancers. Same holds for Ikzf1, Ikzf3 and not IKZF1, IKZF3 etc, throughout the paper.”

We are happy to change if required. However, to the best of our knowledge, protein names should be in upper-case for mouse proteins as well: https://www.uniprot.org/uniprot/Q64131 https://www.uniprot.org/uniprot/?query=ikzf&sort=score

Also see: “*Mice and rats:* Gene symbols are italicized, with only the first letter in upper-case (e.g., *Gfap*). Protein symbols are not italicized, and all letters are in upper-case (e.g., GFAP).” From http://www.biosciencewriters.com/Guidelines-for-Formatting-Gene-and-Protein-Names.aspx

2. “Page 5, please clarify the sentence “Cell heterogeneity within EOMESlo showed more complex patterns: subsets were mainly defined by CD62L, CD49d and CD103 expression”.”

Based on markers used from SPADE analysis, more subpopulations were observed in naïve than T_{IM} cells. To illustrate this, we now provide a modified Supplementary Fig. 1 showing in greater detail these subpopulations and modified the text in the result section:

“Cell heterogeneity within $EOMES^{lo}$ cells showed **more complex bimodal expression** patterns: subsets were mainly defined by CD62L, CD49d and CD103 expression”.

3. “Page 7, a reference should be added after the sentence “Differentiation of CD8+ T lymphocytes into memory cells is accompanied by dynamic changes in the chromatin landscape of promoters and enhancers”.”

We added the references 2 and 3 to this sentence. (He et al Immunity 2016, Scott-Browne et al Immunity 2016)

4. "Page 8, bottom, please clarify the sentence "We identified differentially active enhancers that were common to both models. This allowed us to define consensus sets of enhancer regions that are more (1105 regions) or less (1241 regions) active in conventional memory cells as compared to their naïve counterparts". What is the meaning of more or less active?"

We clarified the text as follows:

"In order to determine whether this was also the case at the epigenetic level, we analysed H3K27ac ChIP-seq data from naïve/T_{CM} P14 cells (LCMV model) and naïve/T_M OT1 cells (Listeria-OVA model)[2,4]. For both models, we performed differential analysis for H3K27Ac peaks that were located in enhancer regions. This allowed us to define consensus sets of enhancers that are more (1105 regions) or less (1241 regions) active in conventional memory cells as compared to their naïve counterparts".

5. "Page 10, why was RIME conducted in activated primary CD8 T cells and not in TIM cells?"

This is purely technical. We obtain 1-2 million T_{IM} from 8-10 Balb/c mice, while the RIME was performed on 50 million cells. We would have needed somewhere between 250-500 Balb/c mice for this single biological question, and opted therefore for an *ex vivo* polyclonal expansion approach for obvious technical and ethical reasons.

6. "It is unclear what was done in Fig. 7d."

- Single-cell fluorescences were retrieved for EOMES and several innate memory cell markers. Given the fact that the bulk of EOMES^{hi} cells arising after IL4c administration in *Smarca4*$^{\Delta T}$ mice show lower EOMES levels than the bulk of *Smarca4*$^{fl/fl}$ EOMES^{hi} cells, we wanted to show that the positive correlations between scEOMES levels and sc levels of these EOMES-induced innate memory markers were negatively impacted by the loss of BRG1. Kendall's Tau is a coefficient of

correlation measuring the association between two variables. Fisher's Z transformation allows the comparison of two coefficients of correlation.

The table from the previous Fig. 7d summarizing the correlation between single-cell fluorescences from *Smarca4^fl/fl^* and *Smarca4^ΔT^* mice has now been moved to supplemental data (Supplementary Fig. 9b).

7. Figure 6a, the number of ATAC regions is the same as in Figure 2e, is it a mistake?

It is not a mistake: as described in the M&M section, "First, we created an atlas containing all obtained peaks for all the populations using bedtools[48] with a minimum overlapping of 1bp". **This includes both T_{IM}/naïve and *Eomes^Tg^*/WT samples.**

Reviewer #2 (Remarks to the Author):

In Figure 3, it seems like clusters 1,3, and 4 (which differ between TIM and conventional memory) should be highlighted more. What do the authors think is the significance of cluster 3?"

This point was raised by reviewer #1. Please refer to detailed responses to points 4, 5 and 6.

"In Figure 5c and Supplemental Fig.4, it would be helpful to include functional responses in BALB/c mice for comparison as was done in Figure 5b for phenotype. If responses are weak in Eomes^Tg by comparison, this may point to the need for additional factors to achieve the enhanced function observed in TIM."

We also agree. We refer you to our detailed responses to the major point 10, related to changes to Fig. 5 and Fig. R3) raised by the Reviewer #1 and that cover this subject.

"As related to Figure 6, what would comparisons between Eomes^Tg and the sorted TIM look like?"

These comparisons are now available as Supplementary Fig. 8. See response to point 13 raised by the Reviewer #1.

"What is the significance of BRG1 based on prior work? More background leading into Figure 7 would be helpful and summary discussion after the data is presented."

We added the following paragraph as background for Fig. 7:

"Previous work indicated that T-BET is able to interact with several epigenetic regulators, including JMJD3, UTX and BRG1[28]. Furthermore, BRG1 was found to be required for optimal T-BET and EOMES-induced *Ifng* expression in transient transfection experiments[28]. We therefore hypothesized that the SWI/SNF machinery could be involved in EOMES-induced T_{IM} development."

Furthermore, as BRG1 also participates to Treg homeostasis, we now provide additional data with mixed bone marrow experiments to demonstrate its intrinsic role in CD8 T cells (Fig. 7d and Supplementary Fig. 9a).

Reviewer #3 (Remarks to the Author):

Major points:

1. There really is not enough evidence to indicate that Eomes is part of the SWI/SNF complex as claimed in the discussion: "We showed that EOMES can be found in complexes containing members of the SWI/SNF machinery, histone deacetylases and their associated DNA-binding ATPase CHD4." In fact, what the authors have shown is that in fixed chromatin, co-immunoprecipitates that are recovered with Eomes-specific antibodies include some (but apparently not all) members of a canonical BAF (Brg1-associated factors) complex. This analysis did not exclude that some of the co-associated proteins were associated by DNA bridging (which might also explain the association of Runx3 with Eomes in these experiments). Although the authors also demonstrate a co-association of Eomes with Brg1 in co-transfection assays, the observed association appears to be weak despite the extreme overexpression conditions, and there were not other positive control blots showing pull-down of other BAF-complex subunits. To conclusively confirm the hypothesis that Eomes is part of a BAF-complex in T cells would require additional extensive biochemical characterization, including isolation of native BAF complexes and demonstrating that Eomes is a part of it. The present findings are interesting, but are still underdeveloped and the conclusions should be adjusted accordingly."

These are all valid points. We modified the text accordingly:

In the abstract: "Furthermore, we showed that EOMES and BRG1 closely interact within chromatin-associated complexes and that the *in vivo* acquisition of EOMES-dependent program by CD8SP thymocytes was dependent on this chromatin remodeling factor

In the result section: "As shown in Fig. 7a, BRG1 and EOMES co-precipitated, suggesting that these proteins can be found in the same molecular complexes".

In the discussion section: "We showed that EOMES in fixed chromatin co-immunoprecipitates with members of the SWI/SNF machinery, histone deacetylases and their associated DNA-binding ATPase CHD4"

> *"2. The authors postulate (in the discussion) that their results "strongly suggest that EOMES allows the recruitment of the SWI/SNF machinery", but their results show that Eomes expression is reduced in TIM in the thymus of Smarca4-deficient mice (and later in the spleen as well), which suggests that Brg1/Smarca4 acts before/upstream of Eomes. This result does not exclude that Eomes also promotes recruitment of Brg1/Smarca4, but without the proper epistatic experiments, this cannot be known for sure. It might be prudent to leave open both possibilities when discussing the results."*

We agree with this comment. To define whether EOMES promotes BRG1 recruitment, we performed ChIP-qPCR experiments in WT or *Eomes^{Tg}* CD8SP cells. We observed increased BRG1 recruitment on several previously identified EOMES binding sites, supporting this notion. These data are now presented in Fig. 7b and described in the result section:

"We further show that EOMES binding to regulatory regions associated with *Il2rb*, *Cxcr3*, *Kdm5b*, *Samd3* and *Stat4* loci in *Eomes^{Tg}* CD8SP thymocytes was accompanied by increased BRG1 recruitment (Fig. 7b). This result suggests that recruitment of BRG1-containing complexes along with EOMES could contribute to CD8SP thymocyte differentiation into T_{IM} cells".

We also modified the conclusion as suggested:

"This chromatin remodeling factor is essential for modulating H3K27ac levels at distal enhancers[39,40]. BRG1 could act upstream of EOMES, through regulation of its expression and/or its recruitment. Our results also strongly suggest that EOMES

facilitates the recruitment of the SWI/SNF machinery to specific cis-regulatory elements that control long-term commitment towards T_{IM} cellular identity.

3. "Although the transgenic Eomes gain-of-function experiments are very convincing, the strength of the conclusion that Eomes promotes the transcriptional activity of its bound cis-regulatory regions is diminished in the absence of the Eomes loss-of-function experiments. An important set of experiments that was not presented but that would be of interest is how Eomes-deficiency affects the transcription and at least some aspects of the chromatin landscape of TIM cells."

We agree that this would be of great interest. We added this point in the discussion section:

"Herein, we show that the ectopic expression of a single transcription factor, EOMES, is sufficient to drive the acquisition of a memory-associated phenotype and of transcriptional and epigenetic profiles in developing CD8SP thymocytes. However not every aspects of the process that takes place during the physiological development of cytokine-driven innate memory (T_{IM}) cells independently of any contact with their foreign cognate antigen were recapitulated in *Eomes*^{Tg} cells. Nevertheless, the increase of this sole transcription factor was able to induce a substantial part of T_{IM} functional, transcriptional and epigenomic features, in a context that is very different from mouse strains that express large amounts of IL-4 in the thymus. We further show that the overexpression of EOMES increases the responsiveness to IL-4, thereby initiating a feed-forward loop. It would be important to formally examine the impact of Eomes deficiency on these different features".

3. "Although it is logical by extension, the strength of the conclusion that Runx3 is involved in the recruitment of Eomes to specific cis-regulatory regions is diminished without examining whether Runx3 is required for this

process, and whether development of TIM cells is impaired in the absence of Runx3. These are challenging experiments because thymic Runx3-disruption will be difficult to achieve without dramatically impairing overall CD8 T cell development, although haploinsufficiency of Runx3 could be telling.”

These experiments are difficult to perform on Balb/c background. Also see our response to reviewer#1 point 7. We rephrased our conclusions as follows:

“ A recent report indicates that RUNX3 might act as a pioneer factor very early during memory commitment[33]. Our results indicate that this could be the case even in the absence of TCR stimulation. RUNX3 deposition in naïve cells COULD establish a favourable chromatin environment for the subsequent recruitment of EOMES”.

“Specific comments/suggestions:

1. There are some places in this paper that describe TIM phenotype with ambiguous descriptions that may lead to misunderstanding of readers to believe it is an earlier stage of typical memory phenotype. For instance, in abstract, “naïve CD8 single positive (SP) thymocytes may already acquire phenotypic and functional characteristics of memory…”. In this sentence, “already” suggests that in the future those cells can become typical memory cells. Moreover, in the introduction, it was described: “… naïve CD8 T cells in a lymphopenic environment undergo conversion to memory phenotype CD8 T cells”. Those cells could be better described as “memory-like” or “virtual memory”, to avoid the confusion of readers. I suggest use unified description for TIM phenotype and avoid ambiguity of typical memory and other types of memory like phenotype throughout the paper.”

We modified the text accordingly to avoid any confusion about terminologies and abbreviations (e.g. “already” has been removed from the abstract sentence) and:

“It has long been known that naïve CD8 T cells in a lymphopenic environment undergo conversion to **memory-like** phenotype CD8 T cells independently of foreign Ag exposure and in response to homeostatic cytokines”.

“2. It would be helpful introduce the potential role of TIM cells in the introduction (which is currently in the discussion).”

We followed this recommendation by moving the paragraph on the potential role of T_{IM} from the discussion to the introduction section.

“3. For the spanning-tree analysis for flow cytometry data, the multi-colors are too diverse, making it a bit difficult to discern which population(s) has higher/lower expression of genes. In addition, there are some more up-to-date unsupervised flow cytometry clustering methods, as described and compared in this review paper:https://www.ncbi.nlm.nih.gov/pubmed/27992111. It seems that FlowSOM / X-shift might have better performance than SPADE especially for rare populations such as TIM in the setting of B6 mice.”

We now provide a modified Supplementary Fig. 1 showing in greater detail these subpopulations. We agree that other efficient unsupervised analysis tools are now available and offer additional interesting features, yet SPADE allowed us to validate properly the populations to sort and get a general sense of cell heterogeneity in the context of unconventional memory formation. We also used a tSNE approach but found SPADE to be more valuable for the questions we were asking.

“4. Some of the conclusions regarding figure 4c are unclear: (1) “Up to 65% of these EOMES peaks overlapped with RUNX3 (Fig 4c).” When we did the math based on the presented Venn diagrams, we get slightly lower values. Can you please clarify how your numbers were calculated? (2) The authors demonstrate a significant (albeit less strong) probability of Runx-motifs occurring within proximity to Eomes binding sites that do not appear to have overlap with Runx3 ChIP-seq peaks (Fig 4C, the 751 Eomes binding sites), but this is not explained carefully, which could confuse

some readers. Does this arise because of potential Runx3 binding sites that were not called as peaks, or because of how the binding site intersections between Runx3 and Eomes regions were computed? If the latter, the current analysis might underestimate the actual co-localization of Runx3/Eomes binding."

- **We obtain 65% when summing up all EOMES peaks specific to T_{IM} cells (promoters and enhancers, thereby summing these data from the two Venn diagrams) and looking at the proportion of these sites that are bound by RUNX3 in naïve and/or T_{IM}.**
- **Regarding the second point, this is in part due to the fact that some Runx3 peaks in these regions were below the threshold of significance that was selected during the peak calling. Alternatively, other Runx factors could bind these regions.**

- **We clarified these points in the result section:**

  "Up to 65% of these EOMES peaks overlapped with RUNX3 peaks obtained from naïve and/or T_{IM} cells"

  "Of note, EOMES-binding regions that were not found to overlap with RUNX3 ChIP-Seq peaks displayed significant enrichment for Runt motifs. This suggests that we probably underestimate the proportion of these regions that are also bound by RUNX3 or other members of the RUNX family".

REVIEWERS' COMMENTS:

Reviewer #2 (Remarks to the Author):

The authors have convincingly addressed my comments and I do not have additional concerns about the manuscript.

Reviewer #3 (Remarks to the Author):

The authors have submitted a substantially improved manuscript, and have sufficiently addressed all of the points we raised. Nice job. We suggest that the authors consider including the following recently published reference, which appears complementary and potentially useful in discussion of their work: van der Veeken J et. al., Immunity. 2019 Apr 16. pii: S1074-7613(19)30148-7.
Matthew E. Pipkin

REVIEWERS' COMMENTS

"Reviewer #2 (Remarks to the Author): The authors have convincingly addressed my comments and I do not have additional concerns about the manuscript.

Reviewer #3 (Remarks to the Author): The authors have submitted a substantially improved manuscript, and have sufficiently addressed all of the points we raised. Nice job. We suggest that the authors consider including the following recently published reference, which appears complementary and potentially useful in discussion of their work: van der Veeken J et. al., Immunity. 2019 Apr 16. pii: S1074- 7613(19)30148-7. Matthew E. Pipkin"

We want to thank again the reviewers for their time and constructive input. The reference Veeken J et al. has been added to the Discussion.
